# Sex and tissue‐specific evolution of developmental plasticity in *Drosophila melanogaster*

**DOI:** 10.1002/ece3.7136

**Published:** 2020-12-17

**Authors:** Didem P. Sarikaya, Katherine Rickelton, Julie M. Cridland, Ryan Hatmaker, Hayley K. Sheehy, Sophia Davis, Nossin Khan, Ashley Kochummen, David J. Begun

**Affiliations:** ^1^ Evolution and Ecology University of California Davis Davis CA USA; ^2^ Molecular and Cellular Biology University of California Davis Davis CA USA

**Keywords:** body size, developmental plasticity, drosophila, evolution, plasticity

## Abstract

Developmental plasticity influences the size of adult tissues in insects. Tissues can have unique responses to environmental perturbation during development; however, the prevalence of within species evolution of tissue‐specific developmental plasticity remains unclear. To address this, we studied the effects of temperature and nutrition on wing and femur size in *D. melanogaster* populations from a temperate and tropical region. Wings were more sensitive to temperature, while wings and femurs were equally responsive to nutrition in both populations and sexes. The temperate population was larger under all conditions, except for femurs of starved females. In line with this, we observed greater femur size plasticity in response to starvation in temperate females, leading to differences in sexual dimorphism between populations such that the slope of the reaction norm of sexual dimorphism in the tropical population was double that of the temperate population. Lastly, we observed a significant trend for steeper slopes of reaction norms in temperate than in tropical females, but not in males. These findings highlight that plasticity divergence between populations can evolve heterogeneously across sexes and tissues and that nutritional plasticity can alter sexual dimorphism in *D. melanogaster*.

## Graphical abstract

Developmental plasticity allows animals to grow under fluctuating environments. Here, we show that the plasticity of one organ (femur) in females has evolved greater plasticity in a temperate population compared to a tropical population, highlighting that plasticity can evolve in a sex‐ and tissue‐specific manner.

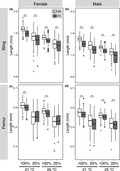

## 1 INTRODUCTION

Developmental plasticity allows for the same genotype to give rise to different phenotypes when juveniles are exposed to varying environmental conditions (Bradshaw, 1965; Stearns, 1989). Plasticity is genetically regulated (Lafuente et al., 2018; Scheiner et al., 1991; Scheiner & Lyman, 1989; Stanley et al., 2017), and theoretical and empirical studies have shown that natural selection may favor genetic variants that increase plasticity in organisms adapting to heterogeneous environments across generations (Gilchrist, 1995; Price et al., 2003).

Morphological traits are particularly sensitive to developmental conditions in insects that undergo metamorphosis because in such insects the size of adult morphological traits is established during development through the proliferation and growth of cells in the imaginal disks (Mirth & Shingleton, 2019). For example, colder developmental temperatures often lead to larger adults, while poorer nutritional conditions often lead to smaller adults in insects (reviewed in (Koyama et al., 2013; Stillwell et al., 2010). Moreover, growth trajectories vary across tissues and thus plasticity response to various environmental parameters can be tissue‐specific (Shingleton et al., 2009; Stern & Emlen, 1999). For example, *D. melanogaster* wings are more sensitive to temperature, femur and thorax are more sensitive to nutrition (Shingleton et al., 2009), and genitalia are the least sensitive to environmental perturbations (Shingleton et al., 2009; Tang et al., 2011). Similarly, horn development is highly sensitive to developmental nutrition in beetles, while genitalia are insensitive (Emlen, 1997). These differences likely arise from changes in the number of cells that seed the imaginal disks, period of time cells is proliferative, and the molecular pathways involved in growth of each disk (Casasa & Moczek, 2018; Emlen et al., 2012; Green & Extavour, 2014; McDonald et al., 2018; Tang et al., 2011). Interspecies comparative studies have found that thermal and nutritional plasticity of one or two tissues can diverge between closely related species (David et al., 1997; Green & Extavour, 2014; Morin et al., 1999; Rohner et al., 2019), which necessarily implies the existence of population genetic variation for plasticity. However, most studies that investigate intraspecific variation in plasticity rely on a single morphological measurement (Blanckenhorn et al., 2018; Clemson et al., 2016; Trotta et al., 2006), which is often used as a proxy for body size, and the prevalence of divergence of tissue‐specific changes in morphological developmental plasticity between populations of the same species remains an open question.

*Drosophila* populations are an attractive model for investigating the evolution of morphological plasticity within species, as many species inhabit a range of habitats with high to low environmental heterogeneity along a latitudinal cline (Adrion et al., 2015). Within species change in plasticity has received the most attention in the context of temperature plasticity in *Drosophila*. Because high latitude populations experience a wide range of temperatures compared to low latitude populations, the expectation is that selection should favor greater developmental plasticity in higher latitude populations. Indeed, this pattern was observed in Australian populations of *D. serrata* and European populations of *D. subobscura*, where temperate populations have higher body size plasticity in response to temperature (Gilchrist & Huey, 2004; Liefting et al., 2009). While it is well established that high latitude populations of *D. melanogaster* from North America, South America, and Australia are larger than low latitude populations (Azevedo et al., 1998; James et al., 1997; Robinson et al., 2000), to date, there is no evidence that developmental plasticity in response to temperature variation differs across populations. In addition to temperature, nutrition quality has a major influence on size across insects (Stillwell et al., 2010). While shifts in nutritional plasticity have been observed across different *Drosophila* species (Green & Extavour, 2014), whether plasticity response to developmental nutritional changes evolves within species in *Drosophila* has not been investigated.

Here, we aim to investigate whether developmental plasticity of morphological traits evolves within species, and if so, whether this process varies across tissues. Specifically, we use temperate and tropical population of *D. melanogaster* to test the hypothesis that (a) developmental plasticity to different environmental factors is tissue‐specific (Shingleton et al., 2009), and (b) plasticity response can evolve in a tissue‐specific manner within species (Gilchrist, 1995; Liefting et al., 2009; Price et al., 2003). To do so, we compared wing and femur size under two temperature and nutritional regimes based on previous work demonstrating the difference in their response to temperature and starvation. We find that wings are more sensitive to temperature changes, but wings and femurs of natural populations are equally sensitive to nutrition. In addition, we identify an increase in plasticity of femur length under starvation in temperate females, suggesting that plasticity responses can evolve in a sex and tissue‐specific manner.

## 2 MATERIAL AND METHODS

### 2.1 Fly stocks and experimental conditions

Ten population lines each from Maine and Panama City were maintained at 21°C or 25°C on standard laboratory media and a 12‐hr light/dark cycle. For rearing experimental subjects, 15–20 females and 10–15 males were placed on 100% or 25% standard *Drosophila* media for 12–24 hr to collect eggs, and larvae were reared in the same vial at 21 or 25°C. Density can strongly influence body size; therefore, vials containing 30–75 pupae were used for measurements. Adults were collected within a day of eclosion, with males and females maintained together. Flies were then placed in 100% EtOH after 5–10 days. Each random sample of wings and femurs for a genotype was sampled from flies deriving from between 3–5 vials to reduce random effects from variation in food or other uncontrolled vial variation. ME and PC flies were subjected to experimental conditions at the same time.

Starvation media was generated by mixing heated standard *Drosophila* media with 1% agar at 1:3 ratio to obtain food diluted by 25%. Food was mixed thoroughly until the media was ~37°C, and 10ml of media was poured into vials. Eclosed adults from starvation media were placed on standard *Drosophila* media until flies were collected for body size measurements.

### 2.2 Body size measurements

Samples stored in 100% EtOH were washed with 50% glycerol twice, then 80% glycerol until samples were coated in glycerol. Wings and first thoracic (T1) legs were removed and mounted on a slide with glycerol as mounting medium and imaged under a Leica microscope. Wing length and width were measured following previously published methods (Lack et al., 2016), and length of the T1 femur was measured using ImageJ (Figure S1).

### 2.3 Statistical analysis

#### 2.3.1 Identifying wing parameter for analysis

Regression analysis on paired wing length and width measurements resulted in a *R* ^2^ value of 0.88, indicating, as expected, that these traits are highly correlated. We present here our analysis of wing length, though all conclusions were highly similar for wing width.

#### 2.3.2 Plasticity analyses

All measurements were log‐transformed for subsequent analyses. We calculated norms of reaction for both wing and femur lengths for each of the sex, temperature, and nutrition variables, holding constant the other two nonfocal variables. Following previous work on plasticity (Gianoli & González‐Teuber, 2005; Gutteling et al., 2007; Lafuente et al., 2018; Ungerer et al., 2003), we calculated the slope of the reaction norm using a linear model in R of the form Model = log (Measurement) ~ Variable, where variable is sex, temperature, or nutrition and measurement is either wing length or femur length for each genotype. The slope estimates the amount of phenotypic difference observed for each genotype under two different treatments, for example, low versus high temperature, while keeping the other variables constant, in this example, sex and nutritional status. The slope for each genotype in a population was used to estimate the mean slope of the population and the null hypothesis that the mean slopes (Table 2) are the same in ME versus PC was tested using a Mann–Whitney *U*/Wilcoxon rank‐sum test.

#### 2.3.3 ANOVA

We performed an analysis of variance (ANOVA) in R. These models estimated the effects of population (with genotype as a nested random effect), sex, temperature, and nutrition on wing and femur length; interaction effects were also estimated. The model for the wing was stated as Wing Model <‐lm(log(Wing Length$Measurement) ~ Wing Length$Population * Wing Length$Population: Wing Length$Genotype * Wing Length$Sex * Wing Length$Temp * Wing Length$Food). The model for femur length was stated as Femur Model <‐ lm(log(Femur Length$Length ~ Femur Length$Population * Femur Length$Population: Femur Length$Genotype * Femur Length$Sex * Femur Length$Temp * Femur Length$Food).

## 3 RESULTS AND DISCUSSION

To investigate the evolution of population differences in developmental plasticity in male and female *D. melanogaster,* we measured wing and femur length in flies from Maine (ME) and Panama City (PC). We selected wing and femur based on Shingleton et al., (2009), who observed higher plasticity response to temperature variation for wing and nutritional variation for femur. Flies were reared at 21 and 25°C on standard *Drosophila* media or media diluted to 25% to induce starvation.

**FIGURE 1:**
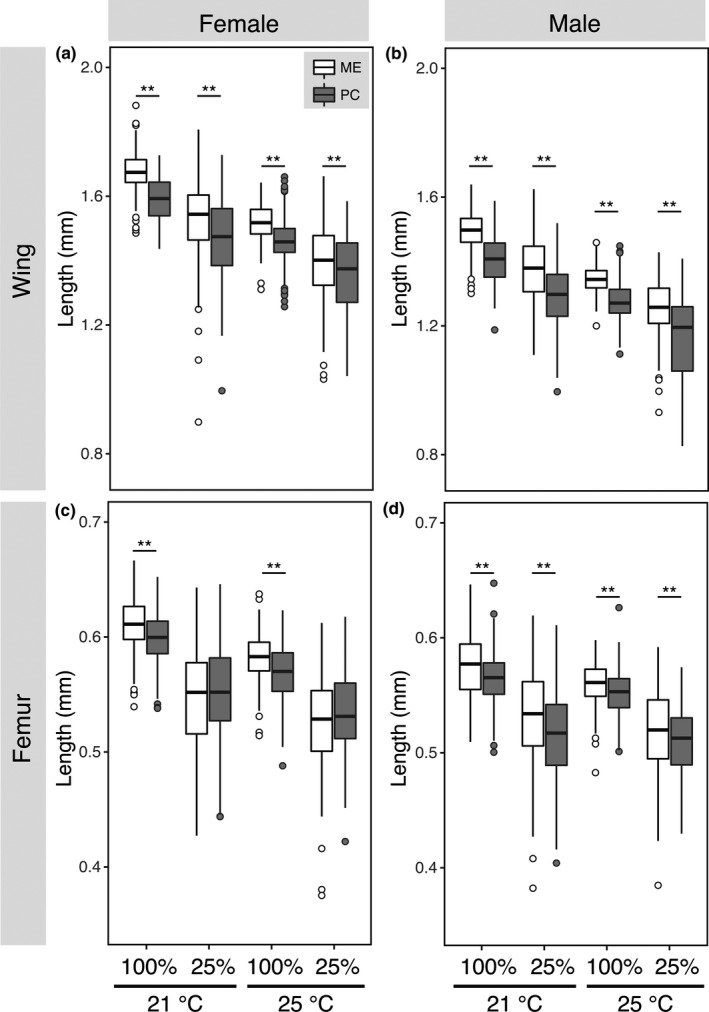
Wing and femur length of ME and PC flies reared under different temperature and nutritional conditions. Boxplots of femur length of (a) females and (b) males, and wing length of (c) females and (d) males. Dietary conditions are listed as 100% for well‐fed and 25% for larvae reared on diluted starvation media. Developmental temperature (21 or 25°C) is listed below the dietary condition. Asterisks indicate two‐way comparisons were statistically significant at *p* < .05

Rearing larvae at 21°C led to a significant increase in size for both wings and femurs (Figure 1). Flies reared at 21°C exhibited a roughly 10% increase in wing size and 5% increase in femur size compared to flies reared at 25°C (Figure 1; Table 1), and rearing larvae under starvation led to ~7%–8% decrease in wing and femur length across populations and sexes compared to well‐fed flies (Figure 1, Table 1). These findings were consistent with previous work on temperature (Azevedo et al., 2002; Robinson & Partridge, 2001; Shingleton et al., 2009) and nutritional plasticity (Beadle et al., 1938; Robertson, 1963; Shingleton et al., 2009) where higher temperature and poorer nutrition reduce size in *D. melanogaster*.

**TABLE 1:** Percent change in wing and femur size under different nutrition and temperature treatments during development

	Female				Male			
	ME		PC		ME		PC	
Wing								
Nutrition								
25°C	8.71%	±1.23	7.29%	±1.97	6.71%	±0.93	6.48%	±0.93
21°C	8.41%	±1.03	7.59%	±1.09	7.87%	±1.38	7.12%	±1.14
Temperature								
Fed	10.33%	±0.93	8.24%	±1.18	11.21%	±0.83	9.24%	±0.78
Starved	10.86%	±3.38	8.66%	±3.72	9.85%	±1.59	9.18%	±3.21
Femur								
Nutrition								
25°C	9.89%	±1.24	6.05%	±1.41	8.88%	±0.92	6.69%	±1.22
21°C	10.57%	±1.58	7.37%	±0.93	7.65%	±2.26	7.02%	±0.93
Temperature								
Fed	5.39%	±5.34	4.16%	±0.51	2.75%	±0.79	3.07%	±0.95
Starved	6.28%	±6.28	3.45%	±1.98	5.48%	±2.17	4.09%	±2.56

Note

Average percent change in size and standard error calculated by comparing 6–10 line averages for each developmental condition showing increase in size of well‐fed individuals compared to starved for the Nutrition column, and increase in size of individuals reared at 21C compared to 25C for the Temperature column. Top four rows contain data for percent change in wing size, and bottom four rows contain data for percent change in femur size.

### 3.1 Wing is more sensitive to temperature, but both wing and femur show similar sensitivity to nutrition

Next, we tested whether wing and femur size had different plasticity responses to temperature and nutrition by comparing the percent change in size and the slope of the reaction norm, which is commonly used for studying within species plasticity response to limited number of environmental parameters (Gianoli & González‐Teuber, 2005; Gutteling et al., 2007; Lafuente et al., 2018; Ungerer et al., 2003).

As described above, rearing flies at 21°C led to ~10% increase in wing length, while femur length increased by 5% (Table 1). The slopes of the reaction norm for temperature were around 0.025 for wing and 0.01 for femur (Table 2). The higher percent change and slope of the reaction norm support the hypothesis that wings are more sensitive than femurs to temperature changes in both ME and PC flies.

**TABLE 2:** Slope of the reaction norm of nutrition and temperature treatments for wing and femur length of ME and PC flies

	Female		Male	
	ME	PC	ME	PC
Wing				
Nutrition				
25°C	0.117	0.108	0.095	0.130
21°C	0.130	0.114	0.110	0.104
Temperature				
Fed	0.025	0.020	0.026	0.023
Starved	0.023	0.019	0.023	0.028
Femur				
Nutrition				
25°C	0.134	0.095	0.112	0.102
21°C	0.152	0.097	0.108	0.130
Temperature				
Fed	0.012	0.009	0.007	0.006
Starved	0.008	0.010	0.010	0.004

Note

Slope of the reaction norms was calculated as the slope of the linear model and average population values were determined by taking an average of 6–10 lines per population. Columns are organized the same as Table 1.

In contrast, plasticity responses to poor nutrition were similar for wing and femur length. Starvation led to a 6%–8% decrease in wing and femur size (Table 1). The slopes of the reaction norm were similar for the two tissues across both populations (Table 2), suggesting that nutrition influences size of femur and wings similarly in both populations. Our findings do not support the hypothesis that femur is more sensitive to developmental nutrition than wing.

The only previous study that found differences in nutritional plasticity between the wing and femur used three lines: one laboratory wild‐type line and two isogenic lines from Maine (Shingleton et al., 2009). Our dataset includes 20 wild lines from two distinct populations. Developmental plasticity is genetically regulated (Debat et al., 2009; Lafuente et al., 2018; Stanley et al., 2017), and the differences in nutritional plasticity observed in our study may be due to the genetic variants found in the ME and PC populations compared to the three strains that were analyzed previously.

Lastly, we used ANOVA to investigate the effects of sex, temperature, nutrition, and population on wing or femur size. Sex was the largest effect variable for wing, though temperature and nutrition effects were also substantial (Table 3). Interestingly, and in contrast to the wing, nutrition had the largest effect on femur, followed by sex and temperature (Table 3). We observed that both Sex * Food and Temperature * Food interaction terms were significant for femur, but not for wing. The ANOVA results show that the two tissues are affected to different degrees by biological and environmental factors.

**TABLE 3:** Linear model estimating the effects of biological (sex, population) and environmental (temperature, food) parameters on wing and femur size

	Wing			Femur		
	Def	*F* value	Pr (>F)	Def	*F* value	Pr (>F)
Population	1	1,195.74	<0.0001*	1	35.9	<0.0001*
Sex	1	6,437.71	<0.0001*	1	744.62	<0.0001*
Temperature	1	3,956.45	<0.0001*	1	397.43	<0.0001*
Food	1	3,238.69	<0.0001*	1	2,611.05	<0.0001*
Population: Genotype	18	90.49	<0.0001*	18	20.82	<0.0001*
Population × Sex	1	32.8	<0.0001*	1	8.92	0.002*
Population × Temperature	1	4.21	0.04*	1	0.71	0.4
Sex × Temperature	1	13.35	0.002*	1	20.74	<0.0001*
Population × Food	1	0.14	0.711	1	21.48	<0.0001*
Sex × Food	1	2.42	0.119	1	7.99	0.005*
Temperature × Food	1	0.23	0.628	1	8.13	0.004*
Population: Genotype × Sex	18	3.55	<0.0001*	17	3.99	<0.0001*
Population: Genotype × Temperature	18	49.95	<0.0001*	17	16.58	<0.0001*
Population × Sex × Temperature	1	1.27	0.26	1	0.04	0.837
Population: Genotype × Food	18	25.5	<0.0001*	18	8.17	<0.0001*
Population × Sex × Food	1	6.33	0.012*	1	17.78	<0.0001*
Population × Temperature × Food	1	11.35	0.007*	1	0.75	0.386
Sex × Temperature × Food	1	0.69	0.407	1	0.11	0.743
Population: Genotype × Sex × Temperature	17	3.18	<0.0001*	15	1.61	0.064
Population: Genotype × Sex × Food	18	3.31	<0.0001*	16	5.4	<0.0001*
Population: Genotype × Temperature × Food	18	22.97	<0.0001*	15	11.02	<0.0001*
Population × Sex × Temperature × Food	1	0.07	0.792	1	2.92	0.087
Population: Genotype × Sex × Temperature × Food	17	1.48	0.092	7	0.84	0.557

Note

For the linear model of the wing length, the adjusted *R*‐squared value was 0.81 and *F*‐statistic was 117.9 on 157 and 4,150 DF. For the linear model of femur length, the adjusted *R*‐squared value was 0.58 and the *F*‐statistic was 36.46 on 138 and 3,464 DF.

* Statistical significance with *p*‐value cut‐off of .05.

### 3.2 Temperate population exhibits higher nutritional plasticity in female femurs

It is well established that higher latitude populations of *Drosophila* are genetically larger than lower latitude populations (Azevedo et al., 1998; James et al., 1997). However, it is unclear whether population differences in body size are also exhibited when flies are nutritionally stressed during development. We compared sizes of ME and PC flies under the various environmental treatment conditions and found that wings and femurs of ME flies were significantly larger than PC flies, except for femurs of females under starvation (Figure 1). Femurs of ME females were significantly larger than femurs of PC females when well‐fed. However, upon starvation, femur size of ME and PC flies was more similar. Stressful developmental conditions, such as high temperature treatments, can reduce size differences across *D. melanogaster* populations (Morin et al., 1999; Trotta et al., 2006). Unlike previous studies, however, we find that nutrition can affect the size of one tissue more drastically than another.

To determine whether plasticity is different across the two populations, we compared their reaction norms. Slopes of reaction norms were generally similar in the two populations, with the exception of female femurs from ME (Table 2), where the slopes of the reaction norm for the ME population were about 50% higher than the PC population. Mann–Whitney *U* test of the slopes of the reaction norm for female femurs showed *p* = .02 for 21°C, but this was not significant after Bonferroni correction. Interestingly, the slopes of the reaction norm of male:female ratio of femur length were almost half the value for ME compared to PC (0.023 for ME and 0.054 for PC at 21°C and 0.018 for ME and 0.042 for PC at 25°C). Mann–Whitney *U* test resulted in *p* = .03 for 25°C and *p* = .02 for 21°C, but these differences were also not significant after Bonferroni correction. However, the magnitude of the difference suggests that the effects of nutrition on female femur size likely lead to population differences in sexual dimorphism. Because the same animals were used for both wing and femur measurements, these differences are unlikely to be attributable solely to individual variation. Table 2 also reveals a statistically significant trend across treatments for greater slopes for ME than for PC females (two‐tailed sign test, *p* = .03) but no such trend for males, further supporting the idea that plasticity divergence differs between sexes. Our ANOVA revealed significant interactions between genotypes nested within population and environmental parameters, but their *F*‐values were considerably lower than the major factors affecting variation in wing and femur size (Table 3), suggesting that there may be additional subtle population differences that were not revealed by our plasticity analyses. Taken together, our results suggest that femurs of ME females evolved higher plasticity response to nutrition compared to the PC population, and that plasticity response to nutrition evolves heterogeneously across sexes and tissues on short timescales in *D. melanogaster*.

To our knowledge, this is the first report of divergent nutritional plasticity response in different *D. melanogaster* populations. While we cannot interpret the population differences as a direct consequence of the measured variables, the extensive literature on latitudinal clines in *D. melanogaster* suggests that these observed differences likely result from local adaptation (Adrion et al., 2015). Similar patterns have been observed in two other *Drosophila* species (Gilchrist & Huey, 2004; Liefting et al., 2009), further supporting the hypothesis that increased plasticity is more likely to evolve in populations experiencing heterogeneous environments (Gilchrist, 1995; Price et al., 2003). The female‐specific shift in nutritional plasticity led to divergent sexual dimorphism in the ME and PC populations, which is consistent with observations from other insect species where changes in nutritional plasticity are the primary driver for within species differences in sexual dimorphism (Fairbairn, 2005; Stillwell et al., 2010).

## 4 CONCLUSION

By examining two environmental parameters and their effect on developmental plasticity of different tissues in two populations, we discovered that the temperate population evolved increased nutritional plasticity of female femur size, leading to divergent sexual dimorphism across the two populations under different diets. Thus, phenotypic plasticity can evolve over short timescales in a sex‐ and tissue‐specific manner.

## CONFLICT OF INTEREST

Authors declare no competing interests or conflict of interests.

## AUTHOR CONTRIBUTION

**Didem Sarikaya:** Conceptualization (lead); Data curation (equal); Formal analysis (equal); Funding acquisition (equal); Investigation (equal); Methodology (equal); Project administration (equal); Supervision (equal); Validation (equal); Visualization (equal); Writing‐original draft (equal); Writing‐review & editing (equal). **Katherine Rickelton:** Data curation (equal); Investigation (equal). **Julie Cridland:** Data curation (equal); Formal analysis (lead); Methodology (equal); Validation (equal); Writing‐review & editing (equal). **Ryan Hatmaker:** Data curation (equal); Investigation (equal). **Hayley Sheehy:** Investigation (equal). **Sophia Davis:** Investigation (equal). **Nossin Khan:** Investigation (equal). **Ashley Kochummen:** Investigation (equal). **David Begun:** Conceptualization (equal); Funding acquisition (equal); Methodology (equal); Project administration (equal); Resources (equal); Supervision (equal); Writing‐original draft (equal); Writing‐review & editing (equal).

## Supporting information

Fig S1

File S1

## Data Availability

All scripts are available on https://github.com/JMCridland/SexandTissueSpecificEvolutioninDmel and raw datasets and figures/tables are available on https://doi.org/10.25338/B81K92.
