# Supplementary figures and images for: Sex and tissue‐specific evolution of developmental plasticity in Drosophila melanogaster
Source: Ecol Evol. 2020 Dec 17;11(3):1334–41. doi: 10.1002/ece3.7136 (PMC7863663; doi:10.1002/ece3.7136)

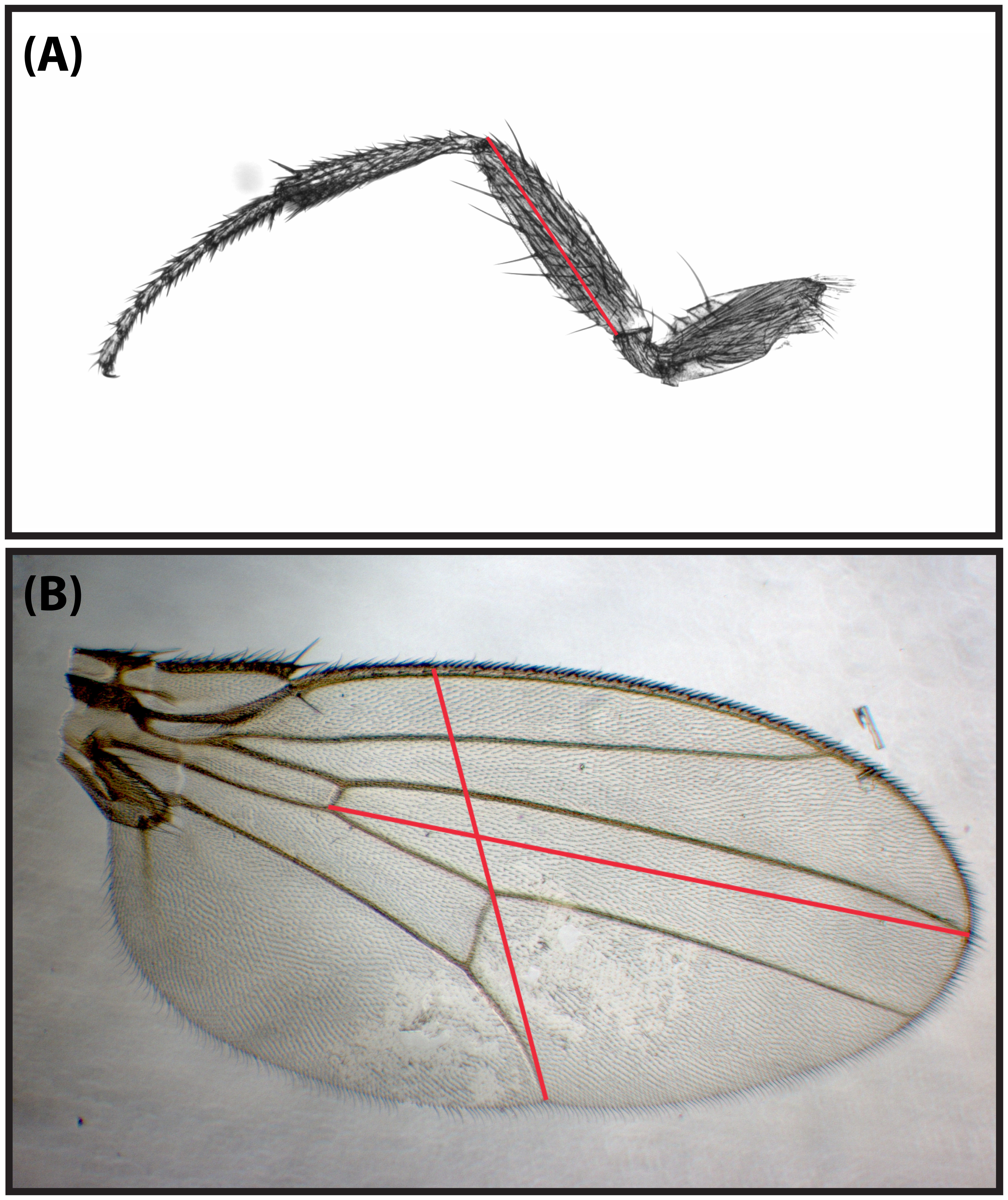

Supplement: Supplementary file 1 — Fig S1 [file ECE3-11-1334-s001.tif]
