# Supplementary material for: Sex and tissue‐specific evolution of developmental plasticity in Drosophila melanogaster
Source: Ecol Evol. 2020 Dec 17;11(3):1334–41. doi: 10.1002/ece3.7136 (PMC7863663; doi:10.1002/ece3.7136)
Supplement: Supplementary file 2 — File S1 [file ECE3-11-1334-s002.pdf]

Supplementary File 1

Julie Cridland

10/28/2020

R Markdown

##Starting with the wing size data.

```
library(ggplot2)
```

```
## Warning: package 'ggplot2' was built under R version 3.6.2
```

```
library(ggthemes)
theme_set(theme_tufte())

x<-read.table("~/Dropbox/Didem_data/Size/Wingsize.txt",header=TRUE)
#going to focus on length as a meausre of size
L<-subset(x,x$Axis=="l")
W<-subset(x,x$Axis=="w")

summary(L)
```

```
##     Genotype      Temp      Population Sex      Food      Axis      Cat
##  FFD16  : 235   High:2155   FFD:2213   F:2144   F:2269   l:4308   21F:1137
##  FFD19  : 233   Low :2153   PC :2095   M:2164   Q:2039   w:   0   21Q:1016
##  FFD29  : 231                                                     25F:1132
##  PC141  : 231                                                     25Q:1023
##  FFD2   : 228
##  PC85   : 225
##  (Other):2925
##   Measurement
##  Min.   :0.8267
##  1st Qu.:1.3105
##  Median :1.4192
##  Mean   :1.4167
##  3rd Qu.:1.5244
##  Max.   :1.8820
##
```

```
summary(W)
```

```
##     Genotype      Temp       Population Sex      Food      Axis      Cat     
##  FFD16  : 235   High:2155   FFD:2214   F:2144   F:2269   l:   0   21F:1137  
##  FFD19  : 233   Low :2154   PC :2095   M:2165   Q:2040   w:4309   21Q:1017  
##  FFD29  : 231                                                     25F:1132  
##  PC141  : 231                                                     25Q:1023  
##  FFD2   : 228                                                               
##  PC85   : 225                                                               
##  (Other):2926                                                               
##   Measurement    
##  Min.   :0.5967  
##  1st Qu.:0.9025  
##  Median :0.9682  
##  Mean   :0.9671  
##  3rd Qu.:1.0385  
##  Max.   :1.5573  
## 
```

```
###Compare the length and width
modl<-lm(log(L$Measurement) ~ L$Temp + L$Population:L$Genotype + L$Sex)
modw<-lm(log(W$Measurement) ~ W$Temp + W$Population:W$Genotype + W$Sex)
print(summary(modl))
```

```
##
## Call:
## lm(formula = log(L$Measurement) ~ L$Temp + L$Population:L$Genotype +
##     L$Sex)
##
## Residuals:
##      Min       1Q   Median       3Q      Max
## -0.57156 -0.03234  0.01706  0.04790  0.17213
##
## Coefficients: (21 not defined because of singularities)
##                                  Estimate Std. Error t value Pr(>|t|)
## (Intercept)                      0.352639   0.005152  68.448  < 2e-16 ***
## L$TempLow                        0.095384   0.002244  42.498  < 2e-16 ***
## L$SexM                          -0.119813   0.002240 -53.490  < 2e-16 ***
## L$PopulationFFD:L$GenotypeFFD10  0.008963   0.006969   1.286   0.1985
## L$PopulationPC:L$GenotypeFFD10         NA         NA      NA       NA
## L$PopulationFFD:L$GenotypeFFD14  0.038314   0.007136   5.369 8.35e-08 ***
## L$PopulationPC:L$GenotypeFFD14         NA         NA      NA       NA
## L$PopulationFFD:L$GenotypeFFD16  0.028854   0.006840   4.219 2.51e-05 ***
## L$PopulationPC:L$GenotypeFFD16         NA         NA      NA       NA
## L$PopulationFFD:L$GenotypeFFD19  0.064308   0.006855   9.382  < 2e-16 ***
## L$PopulationPC:L$GenotypeFFD19         NA         NA      NA       NA
## L$PopulationFFD:L$GenotypeFFD2   0.009085   0.006891   1.318   0.1875
## L$PopulationPC:L$GenotypeFFD2          NA         NA      NA       NA
## L$PopulationFFD:L$GenotypeFFD22  0.016620   0.007045   2.359   0.0184 *
## L$PopulationPC:L$GenotypeFFD22         NA         NA      NA       NA
## L$PopulationFFD:L$GenotypeFFD23  0.061297   0.006922   8.856  < 2e-16 ***
## L$PopulationPC:L$GenotypeFFD23         NA         NA      NA       NA
## L$PopulationFFD:L$GenotypeFFD29  0.004077   0.006869   0.594   0.5528
## L$PopulationPC:L$GenotypeFFD29         NA         NA      NA       NA
## L$PopulationFFD:L$GenotypeFFD4   0.016566   0.006937   2.388   0.0170 *
## L$PopulationPC:L$GenotypeFFD4          NA         NA      NA       NA
## L$PopulationFFD:L$GenotypeFFD6   0.027277   0.007004   3.894 1.00e-04 ***
## L$PopulationPC:L$GenotypeFFD6          NA         NA      NA       NA
## L$PopulationFFD:L$GenotypePC113        NA         NA      NA       NA
## L$PopulationPC:L$GenotypePC113  -0.052375   0.006977  -7.506 7.35e-14 ***
## L$PopulationFFD:L$GenotypePC114        NA         NA      NA       NA
## L$PopulationPC:L$GenotypePC114  -0.091103   0.006979 -13.055  < 2e-16 ***
## L$PopulationFFD:L$GenotypePC12         NA         NA      NA       NA
## L$PopulationPC:L$GenotypePC12    0.001009   0.006986   0.144   0.8852
## L$PopulationFFD:L$GenotypePC136        NA         NA      NA       NA
## L$PopulationPC:L$GenotypePC136  -0.045651   0.007215  -6.327 2.75e-10 ***
## L$PopulationFFD:L$GenotypePC141        NA         NA      NA       NA
## L$PopulationPC:L$GenotypePC141  -0.034621   0.006869  -5.040 4.84e-07 ***
## L$PopulationFFD:L$GenotypePC155        NA         NA      NA       NA
## L$PopulationPC:L$GenotypePC155   0.047544   0.007056   6.739 1.81e-11 ***
## L$PopulationFFD:L$GenotypePC167        NA         NA      NA       NA
## L$PopulationPC:L$GenotypePC167  -0.033590   0.007660  -4.385 1.19e-05 ***
## L$PopulationFFD:L$GenotypePC189        NA         NA      NA       NA
## L$PopulationPC:L$GenotypePC189  -0.056279   0.006937  -8.113 6.41e-16 ***
## L$PopulationFFD:L$GenotypePC200        NA         NA      NA       NA
## L$PopulationPC:L$GenotypePC200   0.012679   0.007019   1.806   0.0709 .
## L$PopulationFFD:L$GenotypePC85         NA         NA      NA       NA
```

```
## L$PopulationPC:L$GenotypePC85           NA          NA      NA       NA
## ---
## Signif. codes:  0 '***' 0.001 '**' 0.01 '*' 0.05 '.' 0.1 ' ' 1
##
## Residual standard error: 0.07333 on 4286 degrees of freedom
## Multiple R-squared:  0.5846, Adjusted R-squared:  0.5825
## F-statistic: 287.2 on 21 and 4286 DF,  p-value: < 2.2e-16
```

```
print(summary(modw))
```

```
##
## Call:
## lm(formula = log(W$Measurement) ~ W$Temp + W$Population:W$Genotype +
##     W$Sex)
##
## Residuals:
##      Min       1Q   Median       3Q      Max
## -0.39189 -0.03539  0.01564  0.05039  0.36401
##
## Coefficients: (21 not defined because of singularities)
##                                  Estimate Std. Error t value Pr(>|t|)
## (Intercept)                     -0.051328   0.005237  -9.802  < 2e-16 ***
## W$TempLow                        0.075817   0.002281  33.239  < 2e-16 ***
## W$SexM                          -0.106044   0.002276 -46.583  < 2e-16 ***
## W$PopulationFFD:W$GenotypeFFD10  0.048662   0.007083   6.870 7.34e-12 ***
## W$PopulationPC:W$GenotypeFFD10         NA         NA      NA       NA
## W$PopulationFFD:W$GenotypeFFD14  0.068833   0.007253   9.490  < 2e-16 ***
## W$PopulationPC:W$GenotypeFFD14         NA         NA      NA       NA
## W$PopulationFFD:W$GenotypeFFD16  0.056907   0.006952   8.186 3.53e-16 ***
## W$PopulationPC:W$GenotypeFFD16         NA         NA      NA       NA
## W$PopulationFFD:W$GenotypeFFD19  0.102008   0.006967  14.642  < 2e-16 ***
## W$PopulationPC:W$GenotypeFFD19         NA         NA      NA       NA
## W$PopulationFFD:W$GenotypeFFD2   0.024452   0.007004   3.491 0.000486 ***
## W$PopulationPC:W$GenotypeFFD2          NA         NA      NA       NA
## W$PopulationFFD:W$GenotypeFFD22  0.060447   0.007160   8.442  < 2e-16 ***
## W$PopulationPC:W$GenotypeFFD22         NA         NA      NA       NA
## W$PopulationFFD:W$GenotypeFFD23  0.077399   0.007035  11.001  < 2e-16 ***
## W$PopulationPC:W$GenotypeFFD23         NA         NA      NA       NA
## W$PopulationFFD:W$GenotypeFFD29  0.043292   0.006981   6.201 6.13e-10 ***
## W$PopulationPC:W$GenotypeFFD29         NA         NA      NA       NA
## W$PopulationFFD:W$GenotypeFFD4   0.054425   0.007043   7.728 1.36e-14 ***
## W$PopulationPC:W$GenotypeFFD4          NA         NA      NA       NA
## W$PopulationFFD:W$GenotypeFFD6   0.039917   0.007119   5.607 2.19e-08 ***
## W$PopulationPC:W$GenotypeFFD6          NA         NA      NA       NA
## W$PopulationFFD:W$GenotypePC113        NA         NA      NA       NA
## W$PopulationPC:W$GenotypePC113  -0.023952   0.007092  -3.377 0.000738 ***
## W$PopulationFFD:W$GenotypePC114        NA         NA      NA       NA
## W$PopulationPC:W$GenotypePC114  -0.072578   0.007093 -10.232  < 2e-16 ***
## W$PopulationFFD:W$GenotypePC12         NA         NA      NA       NA
## W$PopulationPC:W$GenotypePC12    0.021422   0.007101   3.017 0.002569 **
## W$PopulationFFD:W$GenotypePC136        NA         NA      NA       NA
## W$PopulationPC:W$GenotypePC136  -0.023269   0.007333  -3.173 0.001519 **
## W$PopulationFFD:W$GenotypePC141        NA         NA      NA       NA
## W$PopulationPC:W$GenotypePC141   0.018794   0.006981   2.692 0.007130 **
## W$PopulationFFD:W$GenotypePC155        NA         NA      NA       NA
## W$PopulationPC:W$GenotypePC155   0.033242   0.007171   4.635 3.67e-06 ***
## W$PopulationFFD:W$GenotypePC167        NA         NA      NA       NA
## W$PopulationPC:W$GenotypePC167  -0.024743   0.007786  -3.178 0.001494 **
## W$PopulationFFD:W$GenotypePC189        NA         NA      NA       NA
## W$PopulationPC:W$GenotypePC189  -0.009454   0.007051  -1.341 0.180030
## W$PopulationFFD:W$GenotypePC200        NA         NA      NA       NA
## W$PopulationPC:W$GenotypePC200   0.031658   0.007134   4.437 9.34e-06 ***
## W$PopulationFFD:W$GenotypePC85         NA         NA      NA       NA
```

```
## W$PopulationPC:W$GenotypePC85          NA          NA      NA        NA
## ---
## Signif. codes:  0 '***' 0.001 '**' 0.01 '*' 0.05 '.' 0.1 ' ' 1
##
## Residual standard error: 0.07453 on 4287 degrees of freedom
## Multiple R-squared:  0.5182, Adjusted R-squared:  0.5159
## F-statistic: 219.6 on 21 and 4287 DF,  p-value: < 2.2e-16
```

```
print(anova(modl))
```

```
## Analysis of Variance Table
##
## Response: log(L$Measurement)
##                           Df  Sum Sq Mean Sq  F value    Pr(>F)    
## L$Temp                     1  9.5718  9.5718 1780.034 < 2.2e-16 ***
## L$Sex                      1 15.8118 15.8118 2940.450 < 2.2e-16 ***
## L$Population:L$Genotype   19  7.0465  0.3709   68.969 < 2.2e-16 ***
## Residuals               4286 23.0472  0.0054                       
## ---
## Signif. codes:  0 '***' 0.001 '**' 0.01 '*' 0.05 '.' 0.1 ' ' 1
```

```
print(anova(modw))
```

```
## Analysis of Variance Table
##
## Response: log(W$Measurement)
##                           Df  Sum Sq Mean Sq  F value    Pr(>F)    
## W$Temp                     1  6.0432  6.0432 1087.872 < 2.2e-16 ***
## W$Sex                      1 12.4293 12.4293 2237.473 < 2.2e-16 ***
## W$Population:W$Genotype   19  7.1453  0.3761   67.699 < 2.2e-16 ***
## Residuals               4287 23.8146  0.0056                       
## ---
## Signif. codes:  0 '***' 0.001 '**' 0.01 '*' 0.05 '.' 0.1 ' ' 1
```

```
##Names are ordered Population Temperature Sex Food
```

##First look at the population level patterns at different temperatures for the fully fed group.

```
High_F<-subset(x,x$Temp=="High"&x$Food=="F"&x$Axis=="l")

ggplot(data=High_F, aes(Population,log(Measurement))) + geom_boxplot(aes(fill=Sex),width
=0.4) + theme_tufte() + coord_cartesian(ylim = c(0, 1)) + ggtitle("High_Fed")
```

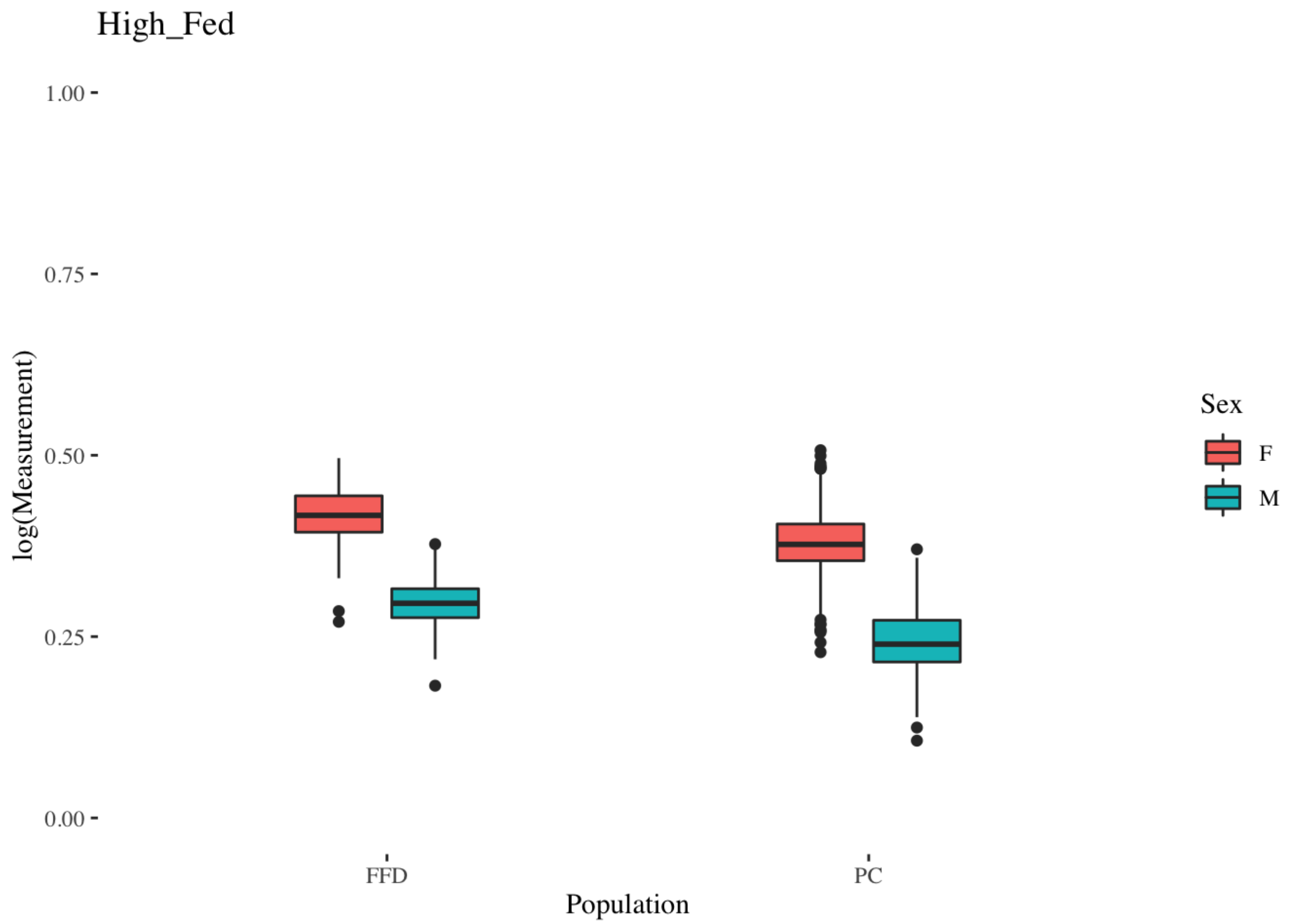


```
Low_F<-subset(x,x$Temp=="Low"&x$Food=="F"&x$Axis=="l")

ggplot(data=Low_F, aes(Population,log(Measurement))) + geom_boxplot(aes(fill=Sex),width=
0.4) + theme_tufte() + coord_cartesian(ylim = c(0,1)) + ggtitle("Low_Fed")
```

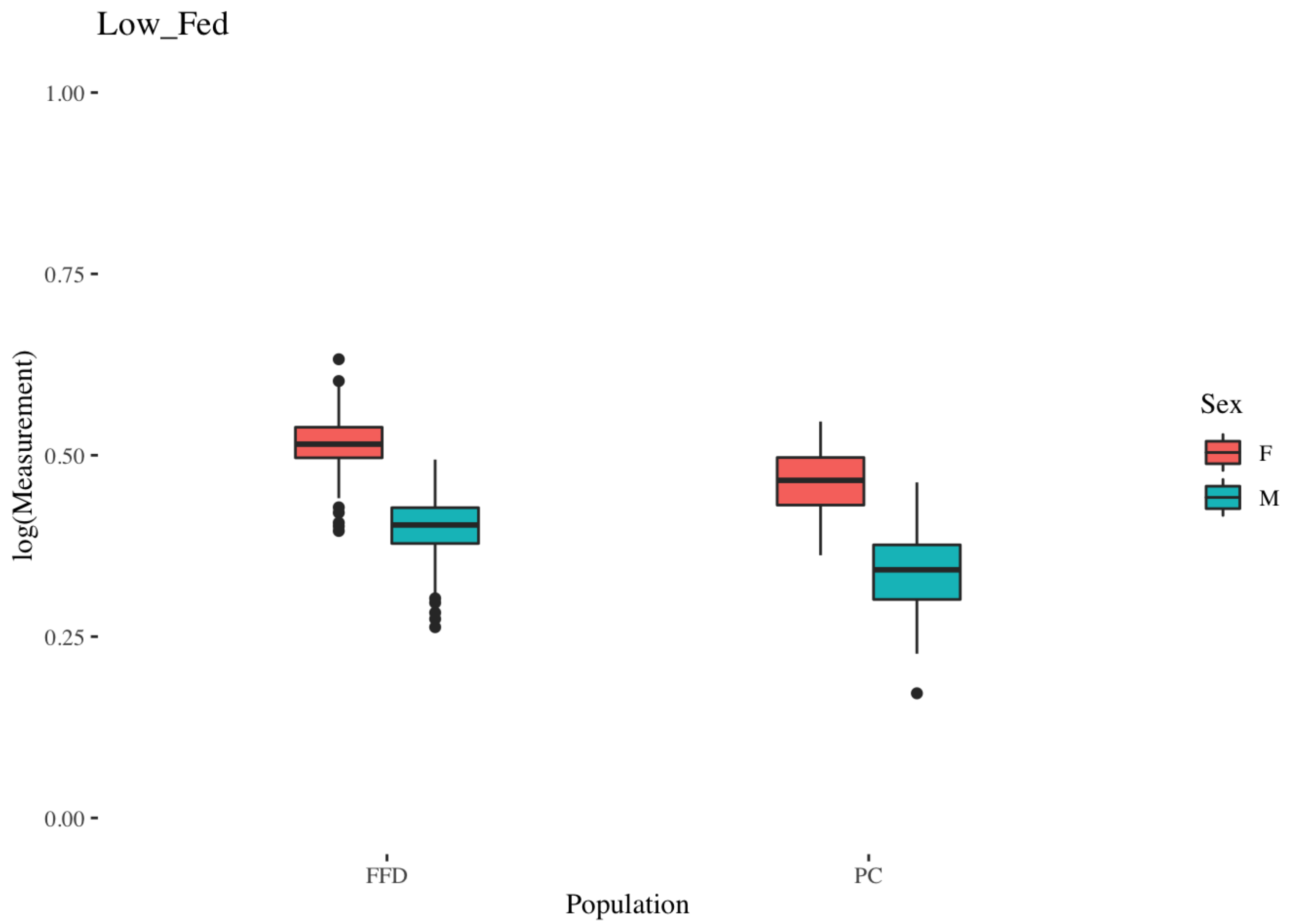

###Next look at the population level patterns at different temperatures for quarter food group.

```
High_Q<-subset(x,x$Temp=="High"&x$Food=="Q"&x$Axis=="l")

Low_Q<-subset(x,x$Temp=="Low"&x$Food=="Q"&x$Axis=="l")


ggplot(data=High_Q, aes(Population,log(Measurement))) + geom_boxplot(aes(fill=Sex),width
=0.7) + theme_tufte() + coord_cartesian(ylim = c(0.1,0.6)) + ggtitle("High_Fed") + theme
(axis.text.x = element_text(angle = 90, hjust = 1))
```

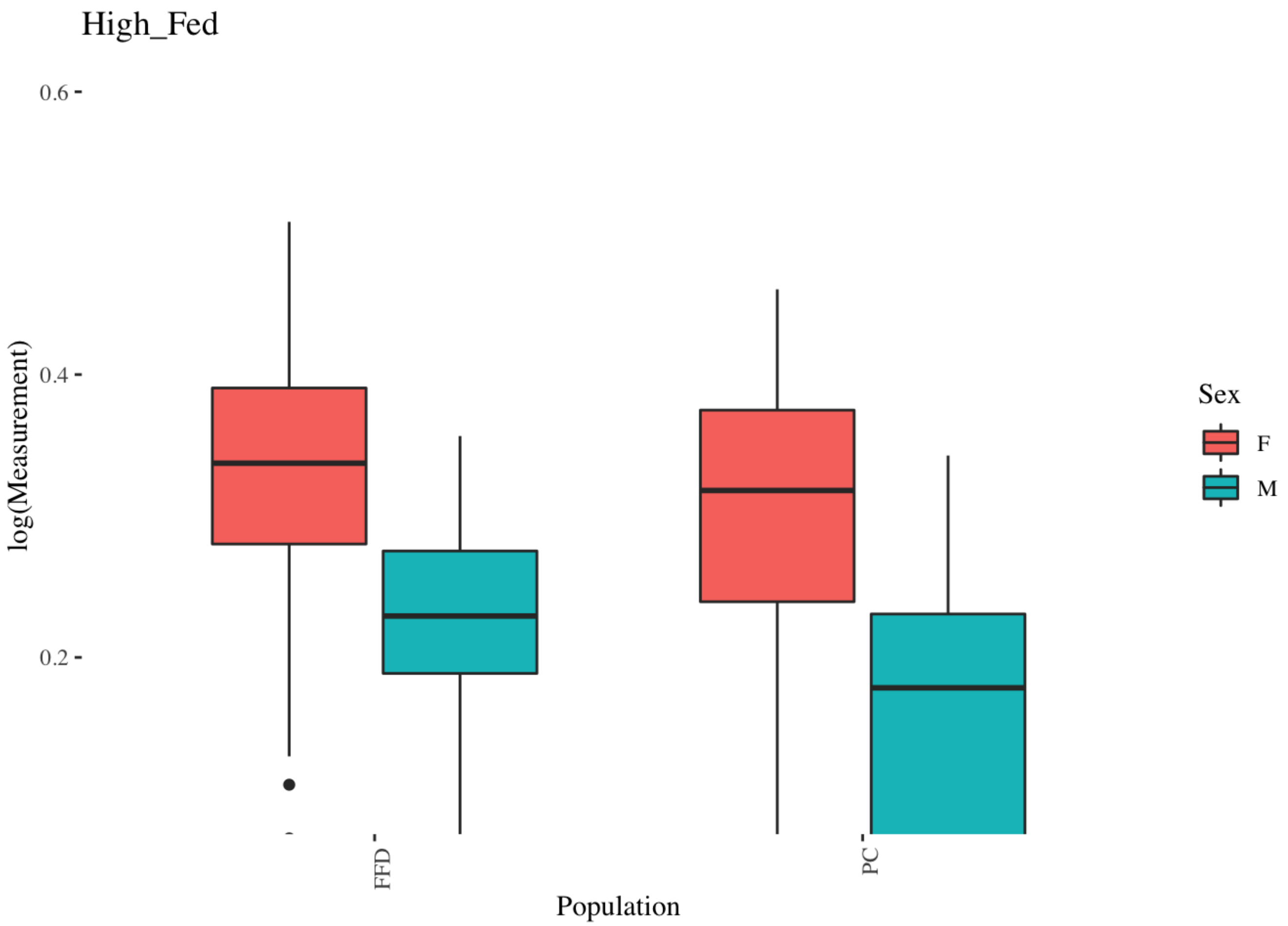


```
ggplot(data=Low_Q, aes(Population,log(Measurement))) + geom_boxplot(aes(fill=Sex),width=
0.7) + theme_tufte() + coord_cartesian(ylim = c(0.1, 0.6)) + ggtitle("Low_Fed") + theme
(axis.text.x = element_text(angle = 90, hjust = 1))
```

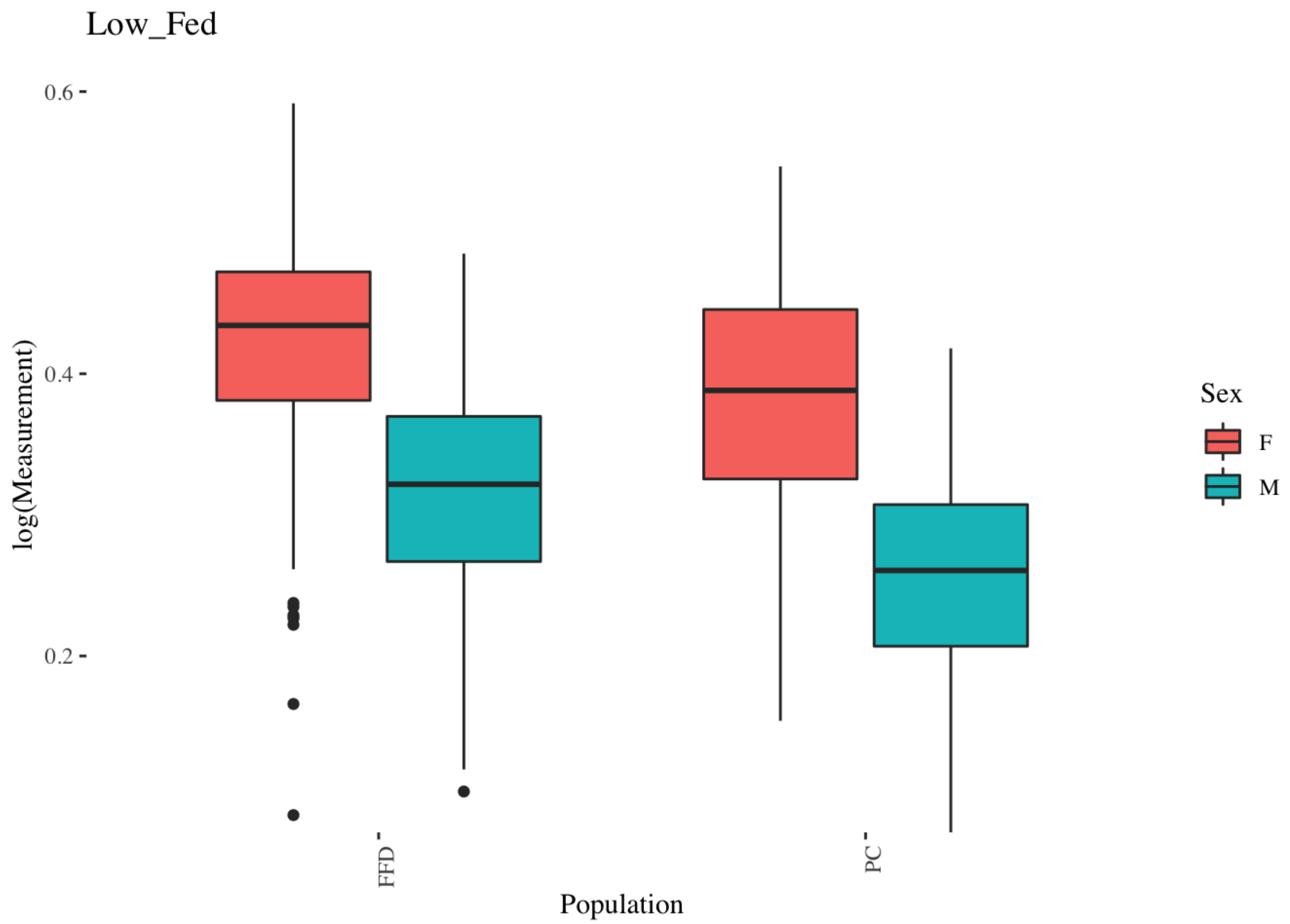

###Next look at differences between temperatures for the fully fed group.

```
FF<-subset(x,x$Food=="F"&x$Sex=="F"&x$Axis=="l")

ggplot(data=FF, aes(Population,log(Measurement))) + geom_boxplot(aes(fill=Temp),width=0.
7) + theme_tufte() + coord_cartesian(ylim = c(0.1, 0.6)) + ggtitle("Females Fed") + them
e(axis.text.x = element_text(angle = 90, hjust = 1))
```

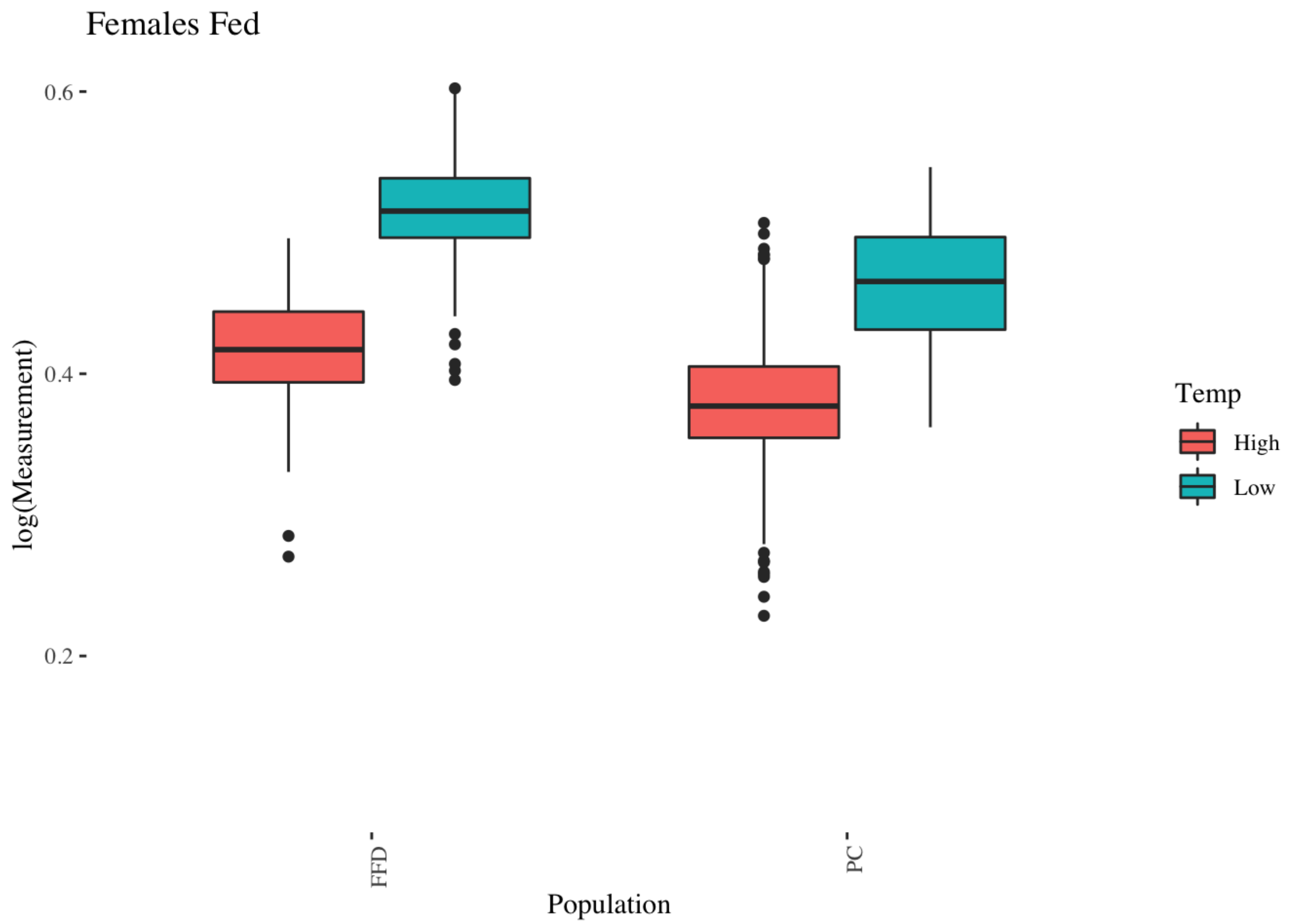


```
MF<-subset(x,x$Food=="F"&x$Sex=="M"&x$Axis=="l")

ggplot(data=MF, aes(Population,log(Measurement))) + geom_boxplot(aes(fill=Temp),width=0.
7) + theme_tufte() + coord_cartesian(ylim = c(0.1, 0.6)) + ggtitle("Males Fed") + theme
(axis.text.x = element_text(angle = 90, hjust = 1))
```

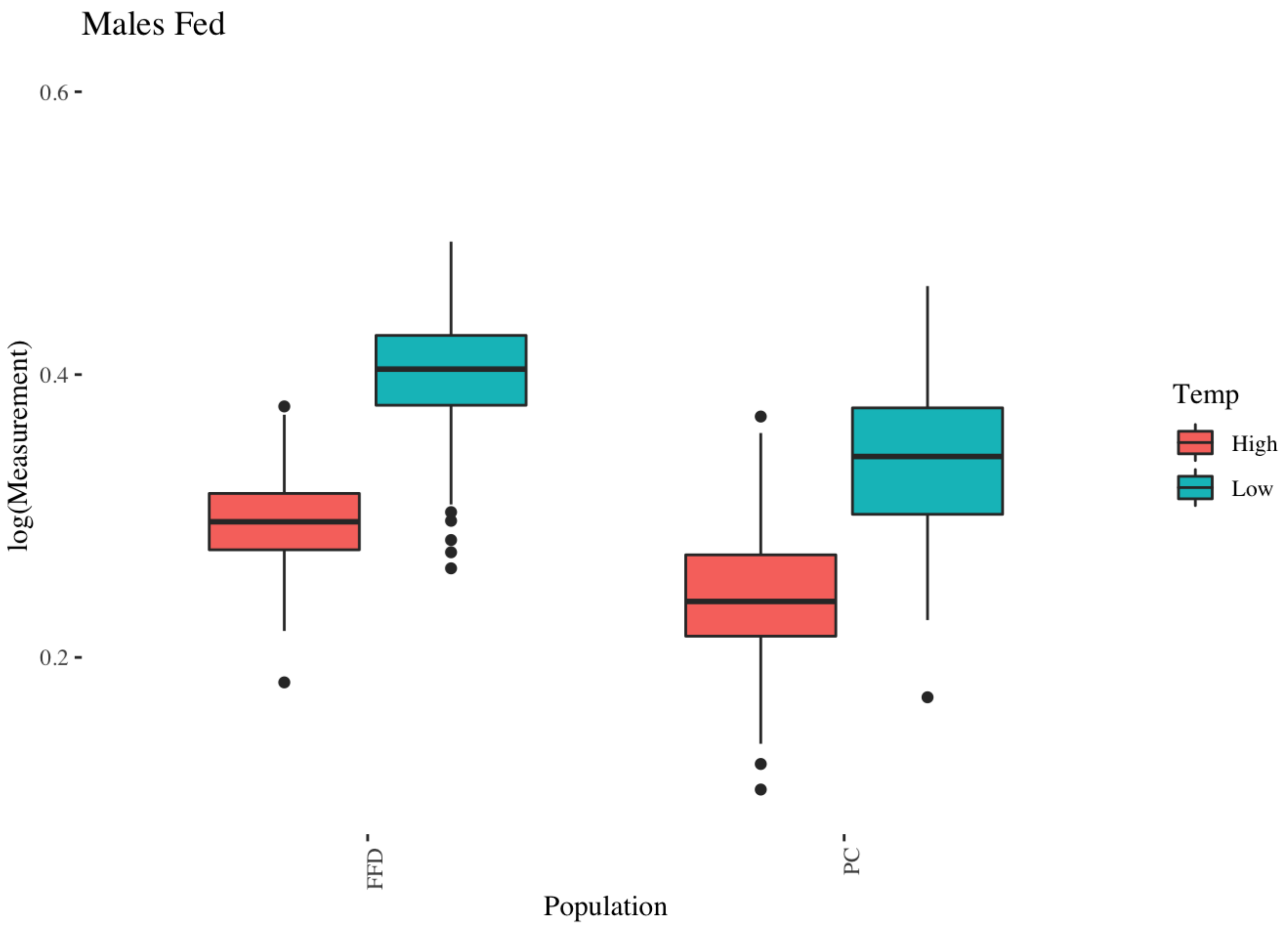

###Next repeat the temperature analysis for the quarter food group.

```
FQ<-subset(x,x$Food=="Q"&x$Sex=="F"&x$Axis=="l")

ggplot(data=FQ, aes(Population,log(Measurement))) + geom_boxplot(aes(fill=Temp),width=0.
7) + theme_tufte() + coord_cartesian(ylim = c(0.1, 0.6)) + ggtitle("Females Quarter") +
 theme(axis.text.x = element_text(angle = 90, hjust = 1))
```

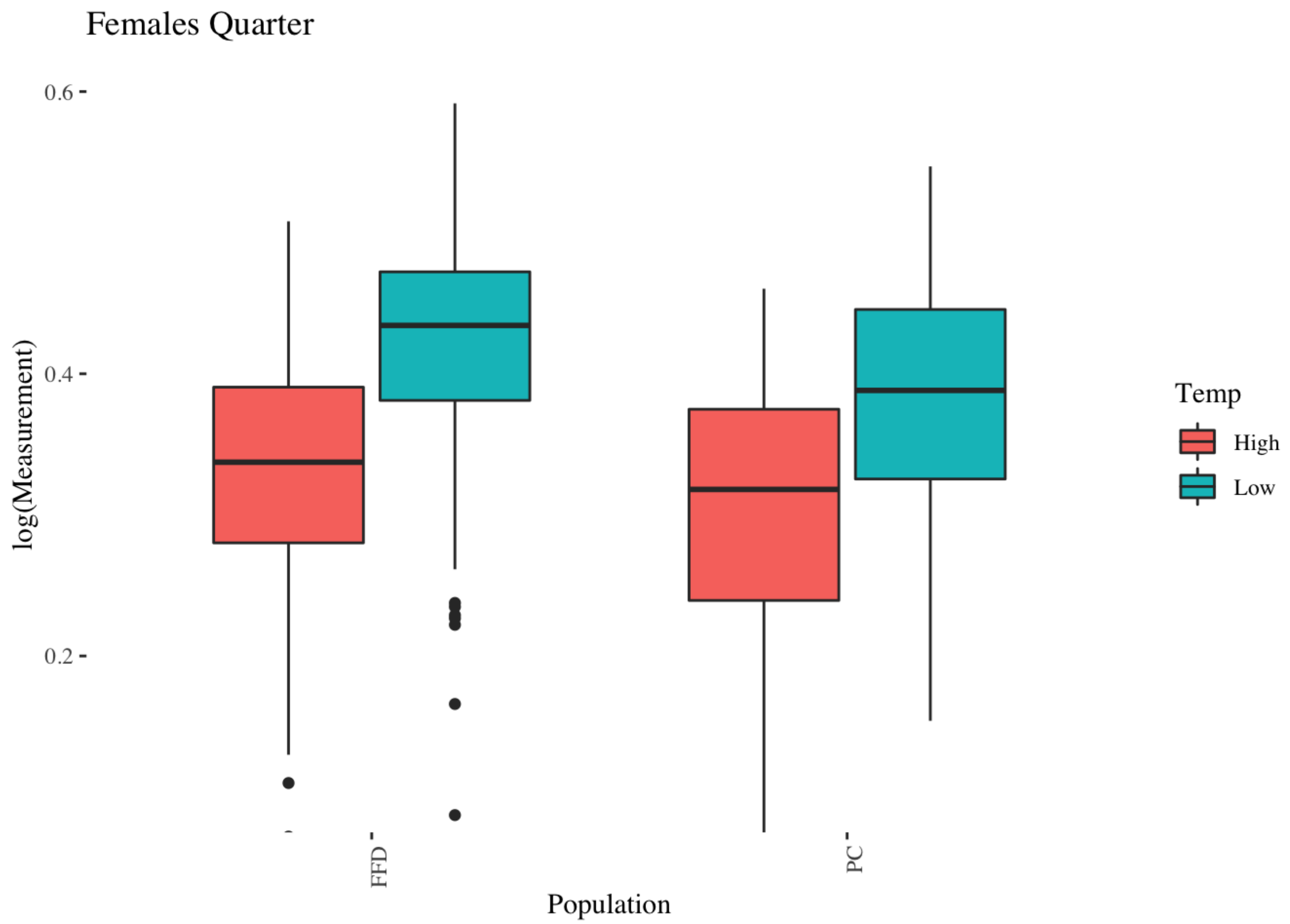


```
MQ<-subset(x,x$Food=="Q"&x$Sex=="M"&x$Axis=="l")

ggplot(data=MQ, aes(Population,log(Measurement))) + geom_boxplot(aes(fill=Temp),width=0.
7) + theme_tufte() + coord_cartesian(ylim = c(0.1,0.6)) + ggtitle("Males Quarter") + the
me(axis.text.x = element_text(angle = 90, hjust = 1))
```

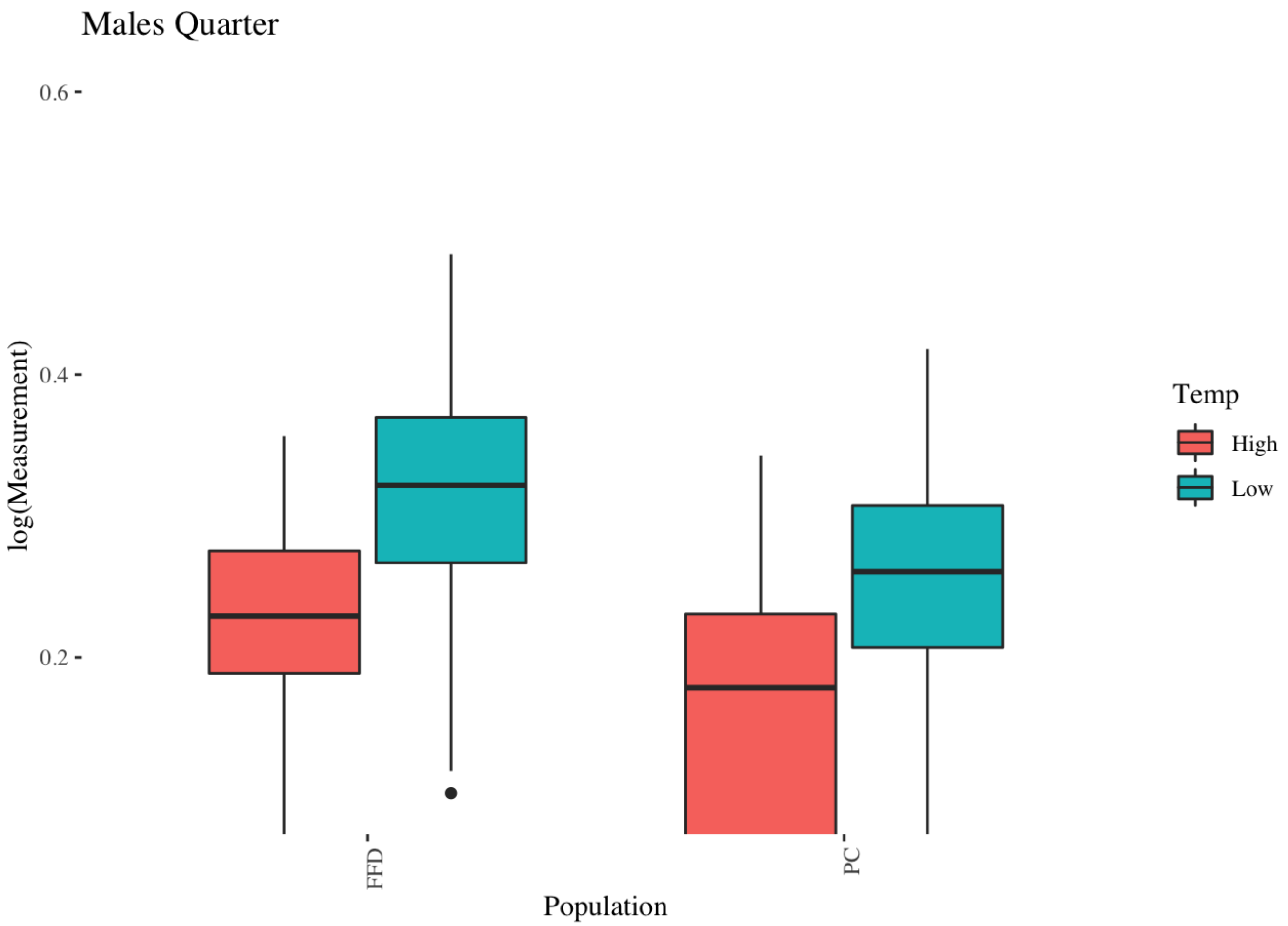

Do some global ANOVAs including nested genotype within population for Wing data

```
GlobalMod<-lm(log(L$Measurement) ~ L$Population*L$Population:L$Genotype*L$Sex*L$Temp*L$F
ood)
print(anova(GlobalMod))
```

```
## Analysis of Variance Table
##
## Response: log(L$Measurement)
##                                               Df  Sum Sq Mean Sq   F value
## L$Population                                   1  2.9282  2.9282 1195.7396
## L$Sex                                          1 15.7648 15.7648 6437.7073
## L$Temp                                         1  9.6887  9.6887 3956.4515
## L$Food                                         1  7.9310  7.9310 3238.6963
## L$Population:L$Genotype                       18  3.9889  0.2216   90.4951
## L$Population:L$Sex                             1  0.0803  0.0803   32.8008
## L$Population:L$Temp                            1  0.0103  0.0103    4.2129
## L$Sex:L$Temp                                   1  0.0327  0.0327   13.3507
## L$Population:L$Food                            1  0.0003  0.0003    0.1377
## L$Sex:L$Food                                   1  0.0059  0.0059    2.4196
## L$Temp:L$Food                                  1  0.0006  0.0006    0.2342
## L$Population:L$Genotype:L$Sex                 18  0.1566  0.0087    3.5531
## L$Population:L$Genotype:L$Temp                18  2.2019  0.1223   49.9527
## L$Population:L$Sex:L$Temp                      1  0.0031  0.0031    1.2683
## L$Population:L$Genotype:L$Food                18  1.1242  0.0625   25.5045
## L$Population:L$Sex:L$Food                      1  0.0155  0.0155    6.3329
## L$Population:L$Temp:L$Food                     1  0.0278  0.0278   11.3467
## L$Sex:L$Temp:L$Food                            1  0.0017  0.0017    0.6854
## L$Population:L$Genotype:L$Sex:L$Temp          17  0.1323  0.0078    3.1777
## L$Population:L$Genotype:L$Sex:L$Food          18  0.1457  0.0081    3.3062
## L$Population:L$Genotype:L$Temp:L$Food         18  1.0123  0.0562   22.9657
## L$Population:L$Sex:L$Temp:L$Food               1  0.0002  0.0002    0.0696
## L$Population:L$Genotype:L$Sex:L$Temp:L$Food   17  0.0616  0.0036    1.4808
## Residuals                                   4150 10.1626  0.0024
##                                                Pr(>F)
## L$Population                                < 2.2e-16 ***
## L$Sex                                       < 2.2e-16 ***
## L$Temp                                      < 2.2e-16 ***
## L$Food                                      < 2.2e-16 ***
## L$Population:L$Genotype                     < 2.2e-16 ***
## L$Population:L$Sex                          1.093e-08 ***
## L$Population:L$Temp                         0.0401803 *
## L$Sex:L$Temp                                0.0002615 ***
## L$Population:L$Food                         0.7105966
## L$Sex:L$Food                                0.1198994
## L$Temp:L$Food                               0.6284307
## L$Population:L$Genotype:L$Sex               5.313e-07 ***
## L$Population:L$Genotype:L$Temp              < 2.2e-16 ***
## L$Population:L$Sex:L$Temp                   0.2601442
## L$Population:L$Genotype:L$Food              < 2.2e-16 ***
## L$Population:L$Sex:L$Food                   0.0118896 *
## L$Population:L$Temp:L$Food                  0.0007627 ***
## L$Sex:L$Temp:L$Food                         0.4077731
## L$Population:L$Genotype:L$Sex:L$Temp        1.078e-05 ***
## L$Population:L$Genotype:L$Sex:L$Food        2.750e-06 ***
## L$Population:L$Genotype:L$Temp:L$Food       < 2.2e-16 ***
## L$Population:L$Sex:L$Temp:L$Food            0.7919803
## L$Population:L$Genotype:L$Sex:L$Temp:L$Food 0.0915607 .
## Residuals
```

```
## ---
## Signif. codes:  0 '***' 0.001 '**' 0.01 '*' 0.05 '.' 0.1 ' ' 1
```

```
print(summary(GlobalMod))
```

```
##
## Call:
## lm(formula = log(L$Measurement) ~ L$Population * L$Population:L$Genotype *
##     L$Sex * L$Temp * L$Food)
##
## Residuals:
##      Min       1Q   Median       3Q      Max
## -0.48172 -0.02058  0.00298  0.02458  0.18894
##
## Coefficients: (162 not defined because of singularities)
##                                                        Estimate Std. Error
## (Intercept)                                           3.907e-01  9.524e-03
## L$PopulationPC                                       -1.121e-02  1.335e-02
## L$SexM                                               -1.381e-01  1.335e-02
## L$TempLow                                             1.043e-01  1.313e-02
## L$FoodQ                                              -7.682e-02  1.374e-02
## L$PopulationFFD:L$GenotypeFFD14                       4.109e-02  1.347e-02
## L$PopulationPC:L$GenotypeFFD14                               NA         NA
## L$PopulationFFD:L$GenotypeFFD16                       3.396e-02  1.313e-02
## L$PopulationPC:L$GenotypeFFD16                               NA         NA
## L$PopulationFFD:L$GenotypeFFD19                       5.179e-02  1.335e-02
## L$PopulationPC:L$GenotypeFFD19                               NA         NA
## L$PopulationFFD:L$GenotypeFFD2                        4.082e-02  1.293e-02
## L$PopulationPC:L$GenotypeFFD2                                NA         NA
## L$PopulationFFD:L$GenotypeFFD22                       3.978e-02  1.388e-02
## L$PopulationPC:L$GenotypeFFD22                               NA         NA
## L$PopulationFFD:L$GenotypeFFD23                       4.398e-02  1.313e-02
## L$PopulationPC:L$GenotypeFFD23                               NA         NA
## L$PopulationFFD:L$GenotypeFFD29                       7.429e-03  1.335e-02
## L$PopulationPC:L$GenotypeFFD29                               NA         NA
## L$PopulationFFD:L$GenotypeFFD4                        3.592e-03  1.284e-02
## L$PopulationPC:L$GenotypeFFD4                                NA         NA
## L$PopulationFFD:L$GenotypeFFD6                        1.490e-02  1.303e-02
## L$PopulationPC:L$GenotypeFFD6                                NA         NA
## L$PopulationFFD:L$GenotypePC113                              NA         NA
## L$PopulationPC:L$GenotypePC113                       -2.647e-02  1.300e-02
## L$PopulationFFD:L$GenotypePC114                              NA         NA
## L$PopulationPC:L$GenotypePC114                       -5.725e-02  1.300e-02
## L$PopulationFFD:L$GenotypePC12                               NA         NA
## L$PopulationPC:L$GenotypePC12                         3.781e-03  1.311e-02
## L$PopulationFFD:L$GenotypePC136                              NA         NA
## L$PopulationPC:L$GenotypePC136                       -2.127e-02  1.300e-02
## L$PopulationFFD:L$GenotypePC141                              NA         NA
## L$PopulationPC:L$GenotypePC141                       -1.493e-02  1.311e-02
## L$PopulationFFD:L$GenotypePC155                              NA         NA
## L$PopulationPC:L$GenotypePC155                        8.398e-02  1.323e-02
## L$PopulationFFD:L$GenotypePC167                              NA         NA
## L$PopulationPC:L$GenotypePC167                       -3.078e-02  2.245e-02
## L$PopulationFFD:L$GenotypePC189                              NA         NA
## L$PopulationPC:L$GenotypePC189                        2.893e-03  1.300e-02
## L$PopulationFFD:L$GenotypePC200                              NA         NA
## L$PopulationPC:L$GenotypePC200                        3.222e-02  1.300e-02
## L$PopulationFFD:L$GenotypePC85                               NA         NA
```

```
## L$PopulationPC:L$GenotypePC85                             NA         NA
## L$PopulationPC:L$SexM                               2.945e-02  1.879e-02
## L$PopulationPC:L$TempLow                           -8.771e-04  1.848e-02
## L$SexM:L$TempLow                                   -5.573e-03  1.848e-02
## L$PopulationPC:L$FoodQ                              2.765e-02  1.956e-02
## L$SexM:L$FoodQ                                      2.570e-02  1.945e-02
## L$TempLow:L$FoodQ                                   1.310e-02  1.930e-02
## L$PopulationFFD:L$GenotypeFFD14:L$SexM             -4.931e-03  1.872e-02
## L$PopulationPC:L$GenotypeFFD14:L$SexM                      NA         NA
## L$PopulationFFD:L$GenotypeFFD16:L$SexM              1.206e-02  1.828e-02
## L$PopulationPC:L$GenotypeFFD16:L$SexM                      NA         NA
## L$PopulationFFD:L$GenotypeFFD19:L$SexM              1.175e-02  1.863e-02
## L$PopulationPC:L$GenotypeFFD19:L$SexM                      NA         NA
## L$PopulationFFD:L$GenotypeFFD2:L$SexM               1.053e-02  1.850e-02
## L$PopulationPC:L$GenotypeFFD2:L$SexM                       NA         NA
## L$PopulationFFD:L$GenotypeFFD22:L$SexM              1.851e-02  1.955e-02
## L$PopulationPC:L$GenotypeFFD22:L$SexM                      NA         NA
## L$PopulationFFD:L$GenotypeFFD23:L$SexM              2.144e-02  1.881e-02
## L$PopulationPC:L$GenotypeFFD23:L$SexM                      NA         NA
## L$PopulationFFD:L$GenotypeFFD29:L$SexM              1.583e-02  1.888e-02
## L$PopulationPC:L$GenotypeFFD29:L$SexM                      NA         NA
## L$PopulationFFD:L$GenotypeFFD4:L$SexM               3.260e-02  1.852e-02
## L$PopulationPC:L$GenotypeFFD4:L$SexM                       NA         NA
## L$PopulationFFD:L$GenotypeFFD6:L$SexM               3.397e-02  1.827e-02
## L$PopulationPC:L$GenotypeFFD6:L$SexM                       NA         NA
## L$PopulationFFD:L$GenotypePC113:L$SexM                     NA         NA
## L$PopulationPC:L$GenotypePC113:L$SexM              -2.109e-02  1.839e-02
## L$PopulationFFD:L$GenotypePC114:L$SexM                     NA         NA
## L$PopulationPC:L$GenotypePC114:L$SexM               1.644e-03  1.873e-02
## L$PopulationFFD:L$GenotypePC12:L$SexM                      NA         NA
## L$PopulationPC:L$GenotypePC12:L$SexM               -4.294e-02  1.847e-02
## L$PopulationFFD:L$GenotypePC136:L$SexM                     NA         NA
## L$PopulationPC:L$GenotypePC136:L$SexM              -5.168e-02  1.839e-02
## L$PopulationFFD:L$GenotypePC141:L$SexM                     NA         NA
## L$PopulationPC:L$GenotypePC141:L$SexM              -1.242e-02  1.839e-02
## L$PopulationFFD:L$GenotypePC155:L$SexM                     NA         NA
## L$PopulationPC:L$GenotypePC155:L$SexM              -2.719e-02  1.855e-02
## L$PopulationFFD:L$GenotypePC167:L$SexM                     NA         NA
## L$PopulationPC:L$GenotypePC167:L$SexM              -3.292e-03  1.822e-02
## L$PopulationFFD:L$GenotypePC189:L$SexM                     NA         NA
## L$PopulationPC:L$GenotypePC189:L$SexM              -6.252e-02  1.839e-02
## L$PopulationFFD:L$GenotypePC200:L$SexM                     NA         NA
## L$PopulationPC:L$GenotypePC200:L$SexM              -1.175e-02  1.847e-02
## L$PopulationFFD:L$GenotypePC85:L$SexM                      NA         NA
## L$PopulationPC:L$GenotypePC85:L$SexM                       NA         NA
## L$PopulationFFD:L$GenotypeFFD14:L$TempLow          -2.831e-02  1.856e-02
## L$PopulationPC:L$GenotypeFFD14:L$TempLow                   NA         NA
## L$PopulationFFD:L$GenotypeFFD16:L$TempLow          -2.641e-02  1.848e-02
## L$PopulationPC:L$GenotypeFFD16:L$TempLow                   NA         NA
## L$PopulationFFD:L$GenotypeFFD19:L$TempLow           6.551e-04  1.881e-02
## L$PopulationPC:L$GenotypeFFD19:L$TempLow                   NA         NA
## L$PopulationFFD:L$GenotypeFFD2:L$TempLow           -4.263e-02  1.834e-02
## L$PopulationPC:L$GenotypeFFD2:L$TempLow                    NA         NA
## L$PopulationFFD:L$GenotypeFFD22:L$TempLow          -3.366e-02  1.920e-02
```

```
## L$PopulationPC:L$GenotypeFFD22:L$TempLow                 NA         NA
## L$PopulationFFD:L$GenotypeFFD23:L$TempLow         3.243e-02  1.840e-02
## L$PopulationPC:L$GenotypeFFD23:L$TempLow                 NA         NA
## L$PopulationFFD:L$GenotypeFFD29:L$TempLow         2.524e-02  1.848e-02
## L$PopulationPC:L$GenotypeFFD29:L$TempLow                 NA         NA
## L$PopulationFFD:L$GenotypeFFD4:L$TempLow          2.822e-02  1.819e-02
## L$PopulationPC:L$GenotypeFFD4:L$TempLow                  NA         NA
## L$PopulationFFD:L$GenotypeFFD6:L$TempLow          6.614e-05  1.849e-02
## L$PopulationPC:L$GenotypeFFD6:L$TempLow                  NA         NA
## L$PopulationFFD:L$GenotypePC113:L$TempLow                NA         NA
## L$PopulationPC:L$GenotypePC113:L$TempLow         -3.567e-02  1.890e-02
## L$PopulationFFD:L$GenotypePC114:L$TempLow                NA         NA
## L$PopulationPC:L$GenotypePC114:L$TempLow          4.279e-02  1.917e-02
## L$PopulationFFD:L$GenotypePC12:L$TempLow                 NA         NA
## L$PopulationPC:L$GenotypePC12:L$TempLow          -5.769e-03  1.875e-02
## L$PopulationFFD:L$GenotypePC136:L$TempLow                NA         NA
## L$PopulationPC:L$GenotypePC136:L$TempLow         -2.342e-02  1.857e-02
## L$PopulationFFD:L$GenotypePC141:L$TempLow                NA         NA
## L$PopulationPC:L$GenotypePC141:L$TempLow         -5.025e-02  1.847e-02
## L$PopulationFFD:L$GenotypePC155:L$TempLow                NA         NA
## L$PopulationPC:L$GenotypePC155:L$TempLow         -5.781e-02  1.855e-02
## L$PopulationFFD:L$GenotypePC167:L$TempLow                NA         NA
## L$PopulationPC:L$GenotypePC167:L$TempLow          3.173e-02  1.838e-02
## L$PopulationFFD:L$GenotypePC189:L$TempLow                NA         NA
## L$PopulationPC:L$GenotypePC189:L$TempLow         -6.038e-02  1.831e-02
## L$PopulationFFD:L$GenotypePC200:L$TempLow                NA         NA
## L$PopulationPC:L$GenotypePC200:L$TempLow         -2.072e-02  1.831e-02
## L$PopulationFFD:L$GenotypePC85:L$TempLow                 NA         NA
## L$PopulationPC:L$GenotypePC85:L$TempLow                  NA         NA
## L$PopulationPC:L$SexM:L$TempLow                  -6.214e-03  2.607e-02
## L$PopulationFFD:L$GenotypeFFD14:L$FoodQ          -2.334e-03  1.942e-02
## L$PopulationPC:L$GenotypeFFD14:L$FoodQ                   NA         NA
## L$PopulationFFD:L$GenotypeFFD16:L$FoodQ           1.805e-02  1.891e-02
## L$PopulationPC:L$GenotypeFFD16:L$FoodQ                   NA         NA
## L$PopulationFFD:L$GenotypeFFD19:L$FoodQ           4.272e-02  1.884e-02
## L$PopulationPC:L$GenotypeFFD19:L$FoodQ                   NA         NA
## L$PopulationFFD:L$GenotypeFFD2:L$FoodQ           -3.410e-02  1.878e-02
## L$PopulationPC:L$GenotypeFFD2:L$FoodQ                    NA         NA
## L$PopulationFFD:L$GenotypeFFD22:L$FoodQ           2.686e-04  1.971e-02
## L$PopulationPC:L$GenotypeFFD22:L$FoodQ                   NA         NA
## L$PopulationFFD:L$GenotypeFFD23:L$FoodQ          -3.093e-02  1.876e-02
## L$PopulationPC:L$GenotypeFFD23:L$FoodQ                   NA         NA
## L$PopulationFFD:L$GenotypeFFD29:L$FoodQ          -8.735e-02  1.891e-02
## L$PopulationPC:L$GenotypeFFD29:L$FoodQ                   NA         NA
## L$PopulationFFD:L$GenotypeFFD4:L$FoodQ           -2.622e-03  1.922e-02
## L$PopulationPC:L$GenotypeFFD4:L$FoodQ                    NA         NA
## L$PopulationFFD:L$GenotypeFFD6:L$FoodQ           -1.480e-02  1.893e-02
## L$PopulationPC:L$GenotypeFFD6:L$FoodQ                    NA         NA
## L$PopulationFFD:L$GenotypePC113:L$FoodQ                  NA         NA
## L$PopulationPC:L$GenotypePC113:L$FoodQ            8.109e-03  1.933e-02
## L$PopulationFFD:L$GenotypePC114:L$FoodQ                  NA         NA
## L$PopulationPC:L$GenotypePC114:L$FoodQ           -1.774e-01  1.890e-02
## L$PopulationFFD:L$GenotypePC12:L$FoodQ                   NA         NA
## L$PopulationPC:L$GenotypePC12:L$FoodQ            -9.520e-03  1.913e-02
```

```
## L$PopulationFFD:L$GenotypePC136:L$FoodQ                     NA         NA
## L$PopulationPC:L$GenotypePC136:L$FoodQ              -7.919e-03  1.967e-02
## L$PopulationFFD:L$GenotypePC141:L$FoodQ                     NA         NA
## L$PopulationPC:L$GenotypePC141:L$FoodQ              -1.003e-02  1.897e-02
## L$PopulationFFD:L$GenotypePC155:L$FoodQ                     NA         NA
## L$PopulationPC:L$GenotypePC155:L$FoodQ              -5.724e-02  1.905e-02
## L$PopulationFFD:L$GenotypePC167:L$FoodQ                     NA         NA
## L$PopulationPC:L$GenotypePC167:L$FoodQ              -2.301e-01  3.318e-02
## L$PopulationFFD:L$GenotypePC189:L$FoodQ                     NA         NA
## L$PopulationPC:L$GenotypePC189:L$FoodQ              -6.051e-02  1.883e-02
## L$PopulationFFD:L$GenotypePC200:L$FoodQ                     NA         NA
## L$PopulationPC:L$GenotypePC200:L$FoodQ               2.936e-02  1.914e-02
## L$PopulationFFD:L$GenotypePC85:L$FoodQ                      NA         NA
## L$PopulationPC:L$GenotypePC85:L$FoodQ                       NA         NA
## L$PopulationPC:L$SexM:L$FoodQ                       -3.971e-02  2.728e-02
## L$PopulationPC:L$TempLow:L$FoodQ                    -5.094e-02  2.696e-02
## L$SexM:L$TempLow:L$FoodQ                             2.741e-02  2.693e-02
## L$PopulationFFD:L$GenotypeFFD14:L$SexM:L$TempLow     2.468e-02  2.597e-02
## L$PopulationPC:L$GenotypeFFD14:L$SexM:L$TempLow             NA         NA
## L$PopulationFFD:L$GenotypeFFD16:L$SexM:L$TempLow     3.407e-02  2.581e-02
## L$PopulationPC:L$GenotypeFFD16:L$SexM:L$TempLow             NA         NA
## L$PopulationFFD:L$GenotypeFFD19:L$SexM:L$TempLow    -7.205e-03  2.631e-02
## L$PopulationPC:L$GenotypeFFD19:L$SexM:L$TempLow             NA         NA
## L$PopulationFFD:L$GenotypeFFD2:L$SexM:L$TempLow      4.610e-02  2.571e-02
## L$PopulationPC:L$GenotypeFFD2:L$SexM:L$TempLow              NA         NA
## L$PopulationFFD:L$GenotypeFFD22:L$SexM:L$TempLow     9.424e-03  2.691e-02
## L$PopulationPC:L$GenotypeFFD22:L$SexM:L$TempLow             NA         NA
## L$PopulationFFD:L$GenotypeFFD23:L$SexM:L$TempLow     4.904e-03  2.614e-02
## L$PopulationPC:L$GenotypeFFD23:L$SexM:L$TempLow             NA         NA
## L$PopulationFFD:L$GenotypeFFD29:L$SexM:L$TempLow     2.083e-02  2.613e-02
## L$PopulationPC:L$GenotypeFFD29:L$SexM:L$TempLow             NA         NA
## L$PopulationFFD:L$GenotypeFFD4:L$SexM:L$TempLow     -1.151e-02  2.593e-02
## L$PopulationPC:L$GenotypeFFD4:L$SexM:L$TempLow              NA         NA
## L$PopulationFFD:L$GenotypeFFD6:L$SexM:L$TempLow     -1.186e-02  2.592e-02
## L$PopulationPC:L$GenotypeFFD6:L$SexM:L$TempLow              NA         NA
## L$PopulationFFD:L$GenotypePC113:L$SexM:L$TempLow            NA         NA
## L$PopulationPC:L$GenotypePC113:L$SexM:L$TempLow      9.734e-03  2.643e-02
## L$PopulationFFD:L$GenotypePC114:L$SexM:L$TempLow            NA         NA
## L$PopulationPC:L$GenotypePC114:L$SexM:L$TempLow     -2.129e-03  2.674e-02
## L$PopulationFFD:L$GenotypePC12:L$SexM:L$TempLow             NA         NA
## L$PopulationPC:L$GenotypePC12:L$SexM:L$TempLow       3.122e-02  2.639e-02
## L$PopulationFFD:L$GenotypePC136:L$SexM:L$TempLow            NA         NA
## L$PopulationPC:L$GenotypePC136:L$SexM:L$TempLow      3.653e-02  2.620e-02
## L$PopulationFFD:L$GenotypePC141:L$SexM:L$TempLow            NA         NA
## L$PopulationPC:L$GenotypePC141:L$SexM:L$TempLow      3.429e-02  2.595e-02
## L$PopulationFFD:L$GenotypePC155:L$SexM:L$TempLow            NA         NA
## L$PopulationPC:L$GenotypePC155:L$SexM:L$TempLow      2.930e-02  2.618e-02
## L$PopulationFFD:L$GenotypePC167:L$SexM:L$TempLow            NA         NA
## L$PopulationPC:L$GenotypePC167:L$SexM:L$TempLow             NA         NA
## L$PopulationFFD:L$GenotypePC189:L$SexM:L$TempLow            NA         NA
## L$PopulationPC:L$GenotypePC189:L$SexM:L$TempLow      3.809e-02  2.589e-02
## L$PopulationFFD:L$GenotypePC200:L$SexM:L$TempLow            NA         NA
## L$PopulationPC:L$GenotypePC200:L$SexM:L$TempLow      1.620e-02  2.589e-02
## L$PopulationFFD:L$GenotypePC85:L$SexM:L$TempLow             NA         NA
```

```
## L$PopulationPC:L$GenotypePC85:L$SexM:L$TempLow                  NA         NA
## L$PopulationFFD:L$GenotypeFFD14:L$SexM:L$FoodQ           2.358e-02  2.776e-02
## L$PopulationPC:L$GenotypeFFD14:L$SexM:L$FoodQ                   NA         NA
## L$PopulationFFD:L$GenotypeFFD16:L$SexM:L$FoodQ          -4.247e-02  2.675e-02
## L$PopulationPC:L$GenotypeFFD16:L$SexM:L$FoodQ                   NA         NA
## L$PopulationFFD:L$GenotypeFFD19:L$SexM:L$FoodQ          -1.338e-02  2.656e-02
## L$PopulationPC:L$GenotypeFFD19:L$SexM:L$FoodQ                   NA         NA
## L$PopulationFFD:L$GenotypeFFD2:L$SexM:L$FoodQ           -1.656e-02  2.672e-02
## L$PopulationPC:L$GenotypeFFD2:L$SexM:L$FoodQ                    NA         NA
## L$PopulationFFD:L$GenotypeFFD22:L$SexM:L$FoodQ          -2.776e-02  2.765e-02
## L$PopulationPC:L$GenotypeFFD22:L$SexM:L$FoodQ                   NA         NA
## L$PopulationFFD:L$GenotypeFFD23:L$SexM:L$FoodQ          -4.185e-03  2.678e-02
## L$PopulationPC:L$GenotypeFFD23:L$SexM:L$FoodQ                   NA         NA
## L$PopulationFFD:L$GenotypeFFD29:L$SexM:L$FoodQ           3.396e-02  2.706e-02
## L$PopulationPC:L$GenotypeFFD29:L$SexM:L$FoodQ                   NA         NA
## L$PopulationFFD:L$GenotypeFFD4:L$SexM:L$FoodQ           -1.952e-02  2.709e-02
## L$PopulationPC:L$GenotypeFFD4:L$SexM:L$FoodQ                    NA         NA
## L$PopulationFFD:L$GenotypeFFD6:L$SexM:L$FoodQ           -2.369e-02  2.662e-02
## L$PopulationPC:L$GenotypeFFD6:L$SexM:L$FoodQ                    NA         NA
## L$PopulationFFD:L$GenotypePC113:L$SexM:L$FoodQ                  NA         NA
## L$PopulationPC:L$GenotypePC113:L$SexM:L$FoodQ            4.779e-03  2.693e-02
## L$PopulationFFD:L$GenotypePC114:L$SexM:L$FoodQ                  NA         NA
## L$PopulationPC:L$GenotypePC114:L$SexM:L$FoodQ           -7.707e-03  2.645e-02
## L$PopulationFFD:L$GenotypePC12:L$SexM:L$FoodQ                   NA         NA
## L$PopulationPC:L$GenotypePC12:L$SexM:L$FoodQ             3.721e-02  2.659e-02
## L$PopulationFFD:L$GenotypePC136:L$SexM:L$FoodQ                  NA         NA
## L$PopulationPC:L$GenotypePC136:L$SexM:L$FoodQ            1.889e-02  2.817e-02
## L$PopulationFFD:L$GenotypePC141:L$SexM:L$FoodQ                  NA         NA
## L$PopulationPC:L$GenotypePC141:L$SexM:L$FoodQ            3.604e-02  2.655e-02
## L$PopulationFFD:L$GenotypePC155:L$SexM:L$FoodQ                  NA         NA
## L$PopulationPC:L$GenotypePC155:L$SexM:L$FoodQ            1.816e-02  2.666e-02
## L$PopulationFFD:L$GenotypePC167:L$SexM:L$FoodQ                  NA         NA
## L$PopulationPC:L$GenotypePC167:L$SexM:L$FoodQ            1.383e-02  2.630e-02
## L$PopulationFFD:L$GenotypePC189:L$SexM:L$FoodQ                  NA         NA
## L$PopulationPC:L$GenotypePC189:L$SexM:L$FoodQ            3.537e-02  2.703e-02
## L$PopulationFFD:L$GenotypePC200:L$SexM:L$FoodQ                  NA         NA
## L$PopulationPC:L$GenotypePC200:L$SexM:L$FoodQ            1.394e-02  2.824e-02
## L$PopulationFFD:L$GenotypePC85:L$SexM:L$FoodQ                   NA         NA
## L$PopulationPC:L$GenotypePC85:L$SexM:L$FoodQ                    NA         NA
## L$PopulationFFD:L$GenotypeFFD14:L$TempLow:L$FoodQ       -1.347e-02  2.755e-02
## L$PopulationPC:L$GenotypeFFD14:L$TempLow:L$FoodQ                NA         NA
## L$PopulationFFD:L$GenotypeFFD16:L$TempLow:L$FoodQ       -3.173e-02  2.665e-02
## L$PopulationPC:L$GenotypeFFD16:L$TempLow:L$FoodQ                NA         NA
## L$PopulationFFD:L$GenotypeFFD19:L$TempLow:L$FoodQ       -5.344e-02  2.678e-02
## L$PopulationPC:L$GenotypeFFD19:L$TempLow:L$FoodQ                NA         NA
## L$PopulationFFD:L$GenotypeFFD2:L$TempLow:L$FoodQ        -7.181e-02  2.721e-02
## L$PopulationPC:L$GenotypeFFD2:L$TempLow:L$FoodQ                 NA         NA
## L$PopulationFFD:L$GenotypeFFD22:L$TempLow:L$FoodQ       -5.579e-02  2.758e-02
## L$PopulationPC:L$GenotypeFFD22:L$TempLow:L$FoodQ                NA         NA
## L$PopulationFFD:L$GenotypeFFD23:L$TempLow:L$FoodQ        2.103e-02  2.679e-02
## L$PopulationPC:L$GenotypeFFD23:L$TempLow:L$FoodQ                NA         NA
## L$PopulationFFD:L$GenotypeFFD29:L$TempLow:L$FoodQ        4.388e-02  2.655e-02
## L$PopulationPC:L$GenotypeFFD29:L$TempLow:L$FoodQ                NA         NA
## L$PopulationFFD:L$GenotypeFFD4:L$TempLow:L$FoodQ        -8.579e-02  2.705e-02
```

```
## L$PopulationPC:L$GenotypeFFD4:L$TempLow:L$FoodQ                   NA         NA
## L$PopulationFFD:L$GenotypeFFD6:L$TempLow:L$FoodQ            1.981e-02  2.678e-02
## L$PopulationPC:L$GenotypeFFD6:L$TempLow:L$FoodQ                   NA         NA
## L$PopulationFFD:L$GenotypePC113:L$TempLow:L$FoodQ                 NA         NA
## L$PopulationPC:L$GenotypePC113:L$TempLow:L$FoodQ            2.039e-02  2.693e-02
## L$PopulationFFD:L$GenotypePC114:L$TempLow:L$FoodQ                 NA         NA
## L$PopulationPC:L$GenotypePC114:L$TempLow:L$FoodQ            2.059e-01  2.699e-02
## L$PopulationFFD:L$GenotypePC12:L$TempLow:L$FoodQ                  NA         NA
## L$PopulationPC:L$GenotypePC12:L$TempLow:L$FoodQ             6.048e-02  2.674e-02
## L$PopulationFFD:L$GenotypePC136:L$TempLow:L$FoodQ                 NA         NA
## L$PopulationPC:L$GenotypePC136:L$TempLow:L$FoodQ           -1.733e-02  2.789e-02
## L$PopulationFFD:L$GenotypePC141:L$TempLow:L$FoodQ                 NA         NA
## L$PopulationPC:L$GenotypePC141:L$TempLow:L$FoodQ            2.487e-02  2.637e-02
## L$PopulationFFD:L$GenotypePC155:L$TempLow:L$FoodQ                 NA         NA
## L$PopulationPC:L$GenotypePC155:L$TempLow:L$FoodQ            9.259e-02  2.663e-02
## L$PopulationFFD:L$GenotypePC167:L$TempLow:L$FoodQ                 NA         NA
## L$PopulationPC:L$GenotypePC167:L$TempLow:L$FoodQ            2.139e-01  2.746e-02
## L$PopulationFFD:L$GenotypePC189:L$TempLow:L$FoodQ                 NA         NA
## L$PopulationPC:L$GenotypePC189:L$TempLow:L$FoodQ            3.326e-02  2.649e-02
## L$PopulationFFD:L$GenotypePC200:L$TempLow:L$FoodQ                 NA         NA
## L$PopulationPC:L$GenotypePC200:L$TempLow:L$FoodQ           -1.022e-01  2.643e-02
## L$PopulationFFD:L$GenotypePC85:L$TempLow:L$FoodQ                  NA         NA
## L$PopulationPC:L$GenotypePC85:L$TempLow:L$FoodQ                   NA         NA
## L$PopulationPC:L$SexM:L$TempLow:L$FoodQ                    -1.074e-03  3.778e-02
## L$PopulationFFD:L$GenotypeFFD14:L$SexM:L$TempLow:L$FoodQ -4.152e-02  3.947e-02
## L$PopulationPC:L$GenotypeFFD14:L$SexM:L$TempLow:L$FoodQ           NA         NA
## L$PopulationFFD:L$GenotypeFFD16:L$SexM:L$TempLow:L$FoodQ -3.780e-02  3.735e-02
## L$PopulationPC:L$GenotypeFFD16:L$SexM:L$TempLow:L$FoodQ           NA         NA
## L$PopulationFFD:L$GenotypeFFD19:L$SexM:L$TempLow:L$FoodQ -1.999e-02  3.741e-02
## L$PopulationPC:L$GenotypeFFD19:L$SexM:L$TempLow:L$FoodQ           NA         NA
## L$PopulationFFD:L$GenotypeFFD2:L$SexM:L$TempLow:L$FoodQ  -2.693e-02  3.778e-02
## L$PopulationPC:L$GenotypeFFD2:L$SexM:L$TempLow:L$FoodQ            NA         NA
## L$PopulationFFD:L$GenotypeFFD22:L$SexM:L$TempLow:L$FoodQ -2.562e-02  3.845e-02
## L$PopulationPC:L$GenotypeFFD22:L$SexM:L$TempLow:L$FoodQ           NA         NA
## L$PopulationFFD:L$GenotypeFFD23:L$SexM:L$TempLow:L$FoodQ -4.948e-02  3.782e-02
## L$PopulationPC:L$GenotypeFFD23:L$SexM:L$TempLow:L$FoodQ           NA         NA
## L$PopulationFFD:L$GenotypeFFD29:L$SexM:L$TempLow:L$FoodQ -9.530e-02  3.749e-02
## L$PopulationPC:L$GenotypeFFD29:L$SexM:L$TempLow:L$FoodQ           NA         NA
## L$PopulationFFD:L$GenotypeFFD4:L$SexM:L$TempLow:L$FoodQ   2.199e-02  3.794e-02
## L$PopulationPC:L$GenotypeFFD4:L$SexM:L$TempLow:L$FoodQ            NA         NA
## L$PopulationFFD:L$GenotypeFFD6:L$SexM:L$TempLow:L$FoodQ  -1.865e-02  3.950e-02
## L$PopulationPC:L$GenotypeFFD6:L$SexM:L$TempLow:L$FoodQ            NA         NA
## L$PopulationFFD:L$GenotypePC113:L$SexM:L$TempLow:L$FoodQ          NA         NA
## L$PopulationPC:L$GenotypePC113:L$SexM:L$TempLow:L$FoodQ  -7.731e-02  3.782e-02
## L$PopulationFFD:L$GenotypePC114:L$SexM:L$TempLow:L$FoodQ          NA         NA
## L$PopulationPC:L$GenotypePC114:L$SexM:L$TempLow:L$FoodQ  -6.090e-02  3.792e-02
## L$PopulationFFD:L$GenotypePC12:L$SexM:L$TempLow:L$FoodQ           NA         NA
## L$PopulationPC:L$GenotypePC12:L$SexM:L$TempLow:L$FoodQ   -3.904e-02  3.785e-02
## L$PopulationFFD:L$GenotypePC136:L$SexM:L$TempLow:L$FoodQ          NA         NA
## L$PopulationPC:L$GenotypePC136:L$SexM:L$TempLow:L$FoodQ   7.382e-03  3.952e-02
## L$PopulationFFD:L$GenotypePC141:L$SexM:L$TempLow:L$FoodQ          NA         NA
## L$PopulationPC:L$GenotypePC141:L$SexM:L$TempLow:L$FoodQ  -5.404e-02  3.718e-02
## L$PopulationFFD:L$GenotypePC155:L$SexM:L$TempLow:L$FoodQ          NA         NA
## L$PopulationPC:L$GenotypePC155:L$SexM:L$TempLow:L$FoodQ  -1.611e-02  3.871e-02
```

```
## L$PopulationFFD:L$GenotypePC167:L$SexM:L$TempLow:L$FoodQ          NA         NA
## L$PopulationPC:L$GenotypePC167:L$SexM:L$TempLow:L$FoodQ           NA         NA
## L$PopulationFFD:L$GenotypePC189:L$SexM:L$TempLow:L$FoodQ          NA         NA
## L$PopulationPC:L$GenotypePC189:L$SexM:L$TempLow:L$FoodQ    1.911e-02  3.771e-02
## L$PopulationFFD:L$GenotypePC200:L$SexM:L$TempLow:L$FoodQ          NA         NA
## L$PopulationPC:L$GenotypePC200:L$SexM:L$TempLow:L$FoodQ    3.784e-04  3.854e-02
## L$PopulationFFD:L$GenotypePC85:L$SexM:L$TempLow:L$FoodQ           NA         NA
## L$PopulationPC:L$GenotypePC85:L$SexM:L$TempLow:L$FoodQ            NA         NA
##                                                            t value Pr(>|t|)
## (Intercept)                                                 41.028  < 2e-16 ***
## L$PopulationPC                                              -0.840 0.401141
## L$SexM                                                     -10.343  < 2e-16 ***
## L$TempLow                                                    7.948 2.42e-15 ***
## L$FoodQ                                                     -5.593 2.38e-08 ***
## L$PopulationFFD:L$GenotypeFFD14                              3.051 0.002296 **
## L$PopulationPC:L$GenotypeFFD14                                  NA       NA
## L$PopulationFFD:L$GenotypeFFD16                              2.587 0.009710 **
## L$PopulationPC:L$GenotypeFFD16                                  NA       NA
## L$PopulationFFD:L$GenotypeFFD19                              3.880 0.000106 ***
## L$PopulationPC:L$GenotypeFFD19                                  NA       NA
## L$PopulationFFD:L$GenotypeFFD2                               3.157 0.001607 **
## L$PopulationPC:L$GenotypeFFD2                                   NA       NA
## L$PopulationFFD:L$GenotypeFFD22                              2.866 0.004183 **
## L$PopulationPC:L$GenotypeFFD22                                  NA       NA
## L$PopulationFFD:L$GenotypeFFD23                              3.351 0.000814 ***
## L$PopulationPC:L$GenotypeFFD23                                  NA       NA
## L$PopulationFFD:L$GenotypeFFD29                              0.557 0.577844
## L$PopulationPC:L$GenotypeFFD29                                  NA       NA
## L$PopulationFFD:L$GenotypeFFD4                               0.280 0.779709
## L$PopulationPC:L$GenotypeFFD4                                   NA       NA
## L$PopulationFFD:L$GenotypeFFD6                               1.144 0.252719
## L$PopulationPC:L$GenotypeFFD6                                   NA       NA
## L$PopulationFFD:L$GenotypePC113                                 NA       NA
## L$PopulationPC:L$GenotypePC113                              -2.036 0.041856 *
## L$PopulationFFD:L$GenotypePC114                                 NA       NA
## L$PopulationPC:L$GenotypePC114                              -4.403 1.09e-05 ***
## L$PopulationFFD:L$GenotypePC12                                  NA       NA
## L$PopulationPC:L$GenotypePC12                                0.288 0.773052
## L$PopulationFFD:L$GenotypePC136                                 NA       NA
## L$PopulationPC:L$GenotypePC136                              -1.636 0.101910
## L$PopulationFFD:L$GenotypePC141                                 NA       NA
## L$PopulationPC:L$GenotypePC141                              -1.138 0.254994
## L$PopulationFFD:L$GenotypePC155                                 NA       NA
## L$PopulationPC:L$GenotypePC155                               6.349 2.39e-10 ***
## L$PopulationFFD:L$GenotypePC167                                 NA       NA
## L$PopulationPC:L$GenotypePC167                              -1.371 0.170405
## L$PopulationFFD:L$GenotypePC189                                 NA       NA
## L$PopulationPC:L$GenotypePC189                               0.222 0.823951
## L$PopulationFFD:L$GenotypePC200                                 NA       NA
## L$PopulationPC:L$GenotypePC200                               2.478 0.013253 *
## L$PopulationFFD:L$GenotypePC85                                  NA       NA
## L$PopulationPC:L$GenotypePC85                                   NA       NA
## L$PopulationPC:L$SexM                                        1.567 0.117145
## L$PopulationPC:L$TempLow                                    -0.047 0.962141
```

```
## L$SexM:L$TempLow                                    -0.302 0.762969
## L$PopulationPC:L$FoodQ                               1.414 0.157519
## L$SexM:L$FoodQ                                       1.322 0.186308
## L$TempLow:L$FoodQ                                    0.679 0.497331
## L$PopulationFFD:L$GenotypeFFD14:L$SexM              -0.263 0.792271
## L$PopulationPC:L$GenotypeFFD14:L$SexM                   NA       NA
## L$PopulationFFD:L$GenotypeFFD16:L$SexM               0.660 0.509258
## L$PopulationPC:L$GenotypeFFD16:L$SexM                   NA       NA
## L$PopulationFFD:L$GenotypeFFD19:L$SexM               0.631 0.528306
## L$PopulationPC:L$GenotypeFFD19:L$SexM                   NA       NA
## L$PopulationFFD:L$GenotypeFFD2:L$SexM                0.569 0.569066
## L$PopulationPC:L$GenotypeFFD2:L$SexM                    NA       NA
## L$PopulationFFD:L$GenotypeFFD22:L$SexM               0.947 0.343900
## L$PopulationPC:L$GenotypeFFD22:L$SexM                   NA       NA
## L$PopulationFFD:L$GenotypeFFD23:L$SexM               1.140 0.254506
## L$PopulationPC:L$GenotypeFFD23:L$SexM                   NA       NA
## L$PopulationFFD:L$GenotypeFFD29:L$SexM               0.839 0.401792
## L$PopulationPC:L$GenotypeFFD29:L$SexM                   NA       NA
## L$PopulationFFD:L$GenotypeFFD4:L$SexM                1.760 0.078430 .
## L$PopulationPC:L$GenotypeFFD4:L$SexM                    NA       NA
## L$PopulationFFD:L$GenotypeFFD6:L$SexM                1.860 0.063026 .
## L$PopulationPC:L$GenotypeFFD6:L$SexM                    NA       NA
## L$PopulationFFD:L$GenotypePC113:L$SexM                  NA       NA
## L$PopulationPC:L$GenotypePC113:L$SexM               -1.147 0.251396
## L$PopulationFFD:L$GenotypePC114:L$SexM                  NA       NA
## L$PopulationPC:L$GenotypePC114:L$SexM                0.088 0.930060
## L$PopulationFFD:L$GenotypePC12:L$SexM                   NA       NA
## L$PopulationPC:L$GenotypePC12:L$SexM                -2.325 0.020106 *
## L$PopulationFFD:L$GenotypePC136:L$SexM                  NA       NA
## L$PopulationPC:L$GenotypePC136:L$SexM               -2.811 0.004969 **
## L$PopulationFFD:L$GenotypePC141:L$SexM                  NA       NA
## L$PopulationPC:L$GenotypePC141:L$SexM               -0.675 0.499524
## L$PopulationFFD:L$GenotypePC155:L$SexM                  NA       NA
## L$PopulationPC:L$GenotypePC155:L$SexM               -1.466 0.142772
## L$PopulationFFD:L$GenotypePC167:L$SexM                  NA       NA
## L$PopulationPC:L$GenotypePC167:L$SexM               -0.181 0.856649
## L$PopulationFFD:L$GenotypePC189:L$SexM                  NA       NA
## L$PopulationPC:L$GenotypePC189:L$SexM               -3.400 0.000681 ***
## L$PopulationFFD:L$GenotypePC200:L$SexM                  NA       NA
## L$PopulationPC:L$GenotypePC200:L$SexM               -0.636 0.524681
## L$PopulationFFD:L$GenotypePC85:L$SexM                   NA       NA
## L$PopulationPC:L$GenotypePC85:L$SexM                    NA       NA
## L$PopulationFFD:L$GenotypeFFD14:L$TempLow           -1.525 0.127406
## L$PopulationPC:L$GenotypeFFD14:L$TempLow                NA       NA
## L$PopulationFFD:L$GenotypeFFD16:L$TempLow           -1.429 0.152986
## L$PopulationPC:L$GenotypeFFD16:L$TempLow                NA       NA
## L$PopulationFFD:L$GenotypeFFD19:L$TempLow            0.035 0.972225
## L$PopulationPC:L$GenotypeFFD19:L$TempLow                NA       NA
## L$PopulationFFD:L$GenotypeFFD2:L$TempLow            -2.325 0.020136 *
## L$PopulationPC:L$GenotypeFFD2:L$TempLow                 NA       NA
## L$PopulationFFD:L$GenotypeFFD22:L$TempLow           -1.753 0.079648 .
## L$PopulationPC:L$GenotypeFFD22:L$TempLow                NA       NA
## L$PopulationFFD:L$GenotypeFFD23:L$TempLow            1.763 0.078022 .
## L$PopulationPC:L$GenotypeFFD23:L$TempLow                NA       NA
```

```
## L$PopulationFFD:L$GenotypeFFD29:L$TempLow                1.366 0.171955    
## L$PopulationPC:L$GenotypeFFD29:L$TempLow                    NA       NA    
## L$PopulationFFD:L$GenotypeFFD4:L$TempLow                 1.551 0.120965    
## L$PopulationPC:L$GenotypeFFD4:L$TempLow                     NA       NA    
## L$PopulationFFD:L$GenotypeFFD6:L$TempLow                 0.004 0.997147    
## L$PopulationPC:L$GenotypeFFD6:L$TempLow                     NA       NA    
## L$PopulationFFD:L$GenotypePC113:L$TempLow                   NA       NA    
## L$PopulationPC:L$GenotypePC113:L$TempLow                -1.887 0.059195 .  
## L$PopulationFFD:L$GenotypePC114:L$TempLow                   NA       NA    
## L$PopulationPC:L$GenotypePC114:L$TempLow                 2.233 0.025614 *  
## L$PopulationFFD:L$GenotypePC12:L$TempLow                    NA       NA    
## L$PopulationPC:L$GenotypePC12:L$TempLow                 -0.308 0.758334    
## L$PopulationFFD:L$GenotypePC136:L$TempLow                   NA       NA    
## L$PopulationPC:L$GenotypePC136:L$TempLow                -1.261 0.207304    
## L$PopulationFFD:L$GenotypePC141:L$TempLow                   NA       NA    
## L$PopulationPC:L$GenotypePC141:L$TempLow                -2.721 0.006534 ** 
## L$PopulationFFD:L$GenotypePC155:L$TempLow                   NA       NA    
## L$PopulationPC:L$GenotypePC155:L$TempLow                -3.117 0.001842 ** 
## L$PopulationFFD:L$GenotypePC167:L$TempLow                   NA       NA    
## L$PopulationPC:L$GenotypePC167:L$TempLow                 1.726 0.084434 .  
## L$PopulationFFD:L$GenotypePC189:L$TempLow                   NA       NA    
## L$PopulationPC:L$GenotypePC189:L$TempLow                -3.298 0.000982 ***
## L$PopulationFFD:L$GenotypePC200:L$TempLow                   NA       NA    
## L$PopulationPC:L$GenotypePC200:L$TempLow                -1.132 0.257887    
## L$PopulationFFD:L$GenotypePC85:L$TempLow                    NA       NA    
## L$PopulationPC:L$GenotypePC85:L$TempLow                     NA       NA    
## L$PopulationPC:L$SexM:L$TempLow                         -0.238 0.811601    
## L$PopulationFFD:L$GenotypeFFD14:L$FoodQ                 -0.120 0.904345    
## L$PopulationPC:L$GenotypeFFD14:L$FoodQ                      NA       NA    
## L$PopulationFFD:L$GenotypeFFD16:L$FoodQ                  0.954 0.340001    
## L$PopulationPC:L$GenotypeFFD16:L$FoodQ                      NA       NA    
## L$PopulationFFD:L$GenotypeFFD19:L$FoodQ                  2.267 0.023445 *  
## L$PopulationPC:L$GenotypeFFD19:L$FoodQ                      NA       NA    
## L$PopulationFFD:L$GenotypeFFD2:L$FoodQ                  -1.816 0.069460 .  
## L$PopulationPC:L$GenotypeFFD2:L$FoodQ                       NA       NA    
## L$PopulationFFD:L$GenotypeFFD22:L$FoodQ                  0.014 0.989128    
## L$PopulationPC:L$GenotypeFFD22:L$FoodQ                      NA       NA    
## L$PopulationFFD:L$GenotypeFFD23:L$FoodQ                 -1.649 0.099221 .  
## L$PopulationPC:L$GenotypeFFD23:L$FoodQ                      NA       NA    
## L$PopulationFFD:L$GenotypeFFD29:L$FoodQ                 -4.618 3.98e-06 ***
## L$PopulationPC:L$GenotypeFFD29:L$FoodQ                      NA       NA    
## L$PopulationFFD:L$GenotypeFFD4:L$FoodQ                  -0.136 0.891464    
## L$PopulationPC:L$GenotypeFFD4:L$FoodQ                       NA       NA    
## L$PopulationFFD:L$GenotypeFFD6:L$FoodQ                  -0.782 0.434327    
## L$PopulationPC:L$GenotypeFFD6:L$FoodQ                       NA       NA    
## L$PopulationFFD:L$GenotypePC113:L$FoodQ                     NA       NA    
## L$PopulationPC:L$GenotypePC113:L$FoodQ                   0.420 0.674826    
## L$PopulationFFD:L$GenotypePC114:L$FoodQ                     NA       NA    
## L$PopulationPC:L$GenotypePC114:L$FoodQ                  -9.387  < 2e-16 ***
## L$PopulationFFD:L$GenotypePC12:L$FoodQ                      NA       NA    
## L$PopulationPC:L$GenotypePC12:L$FoodQ                   -0.498 0.618711    
## L$PopulationFFD:L$GenotypePC136:L$FoodQ                     NA       NA    
## L$PopulationPC:L$GenotypePC136:L$FoodQ                  -0.403 0.687268    
## L$PopulationFFD:L$GenotypePC141:L$FoodQ                     NA       NA    
```

```
## L$PopulationPC:L$GenotypePC141:L$FoodQ                    -0.529 0.596947
## L$PopulationFFD:L$GenotypePC155:L$FoodQ                       NA       NA
## L$PopulationPC:L$GenotypePC155:L$FoodQ                    -3.004 0.002678 **
## L$PopulationFFD:L$GenotypePC167:L$FoodQ                       NA       NA
## L$PopulationPC:L$GenotypePC167:L$FoodQ                    -6.935 4.69e-12 ***
## L$PopulationFFD:L$GenotypePC189:L$FoodQ                       NA       NA
## L$PopulationPC:L$GenotypePC189:L$FoodQ                    -3.213 0.001322 **
## L$PopulationFFD:L$GenotypePC200:L$FoodQ                       NA       NA
## L$PopulationPC:L$GenotypePC200:L$FoodQ                     1.534 0.125011
## L$PopulationFFD:L$GenotypePC85:L$FoodQ                        NA       NA
## L$PopulationPC:L$GenotypePC85:L$FoodQ                         NA       NA
## L$PopulationPC:L$SexM:L$FoodQ                             -1.456 0.145532
## L$PopulationPC:L$TempLow:L$FoodQ                          -1.889 0.058908 .
## L$SexM:L$TempLow:L$FoodQ                                   1.018 0.308760
## L$PopulationFFD:L$GenotypeFFD14:L$SexM:L$TempLow           0.950 0.341960
## L$PopulationPC:L$GenotypeFFD14:L$SexM:L$TempLow               NA       NA
## L$PopulationFFD:L$GenotypeFFD16:L$SexM:L$TempLow           1.320 0.187001
## L$PopulationPC:L$GenotypeFFD16:L$SexM:L$TempLow               NA       NA
## L$PopulationFFD:L$GenotypeFFD19:L$SexM:L$TempLow          -0.274 0.784213
## L$PopulationPC:L$GenotypeFFD19:L$SexM:L$TempLow               NA       NA
## L$PopulationFFD:L$GenotypeFFD2:L$SexM:L$TempLow            1.793 0.072996 .
## L$PopulationPC:L$GenotypeFFD2:L$SexM:L$TempLow                NA       NA
## L$PopulationFFD:L$GenotypeFFD22:L$SexM:L$TempLow           0.350 0.726186
## L$PopulationPC:L$GenotypeFFD22:L$SexM:L$TempLow               NA       NA
## L$PopulationFFD:L$GenotypeFFD23:L$SexM:L$TempLow           0.188 0.851183
## L$PopulationPC:L$GenotypeFFD23:L$SexM:L$TempLow               NA       NA
## L$PopulationFFD:L$GenotypeFFD29:L$SexM:L$TempLow           0.797 0.425495
## L$PopulationPC:L$GenotypeFFD29:L$SexM:L$TempLow               NA       NA
## L$PopulationFFD:L$GenotypeFFD4:L$SexM:L$TempLow           -0.444 0.657195
## L$PopulationPC:L$GenotypeFFD4:L$SexM:L$TempLow                NA       NA
## L$PopulationFFD:L$GenotypeFFD6:L$SexM:L$TempLow           -0.457 0.647443
## L$PopulationPC:L$GenotypeFFD6:L$SexM:L$TempLow                NA       NA
## L$PopulationFFD:L$GenotypePC113:L$SexM:L$TempLow              NA       NA
## L$PopulationPC:L$GenotypePC113:L$SexM:L$TempLow            0.368 0.712669
## L$PopulationFFD:L$GenotypePC114:L$SexM:L$TempLow              NA       NA
## L$PopulationPC:L$GenotypePC114:L$SexM:L$TempLow           -0.080 0.936536
## L$PopulationFFD:L$GenotypePC12:L$SexM:L$TempLow               NA       NA
## L$PopulationPC:L$GenotypePC12:L$SexM:L$TempLow             1.183 0.236820
## L$PopulationFFD:L$GenotypePC136:L$SexM:L$TempLow              NA       NA
## L$PopulationPC:L$GenotypePC136:L$SexM:L$TempLow            1.394 0.163252
## L$PopulationFFD:L$GenotypePC141:L$SexM:L$TempLow              NA       NA
## L$PopulationPC:L$GenotypePC141:L$SexM:L$TempLow            1.321 0.186428
## L$PopulationFFD:L$GenotypePC155:L$SexM:L$TempLow              NA       NA
## L$PopulationPC:L$GenotypePC155:L$SexM:L$TempLow            1.119 0.263097
## L$PopulationFFD:L$GenotypePC167:L$SexM:L$TempLow              NA       NA
## L$PopulationPC:L$GenotypePC167:L$SexM:L$TempLow               NA       NA
## L$PopulationFFD:L$GenotypePC189:L$SexM:L$TempLow              NA       NA
## L$PopulationPC:L$GenotypePC189:L$SexM:L$TempLow            1.471 0.141352
## L$PopulationFFD:L$GenotypePC200:L$SexM:L$TempLow              NA       NA
## L$PopulationPC:L$GenotypePC200:L$SexM:L$TempLow            0.626 0.531479
## L$PopulationFFD:L$GenotypePC85:L$SexM:L$TempLow               NA       NA
## L$PopulationPC:L$GenotypePC85:L$SexM:L$TempLow                NA       NA
## L$PopulationFFD:L$GenotypeFFD14:L$SexM:L$FoodQ             0.849 0.395717
## L$PopulationPC:L$GenotypeFFD14:L$SexM:L$FoodQ                 NA       NA
```

```
## L$PopulationFFD:L$GenotypeFFD16:L$SexM:L$FoodQ          -1.588 0.112445
## L$PopulationPC:L$GenotypeFFD16:L$SexM:L$FoodQ               NA       NA
## L$PopulationFFD:L$GenotypeFFD19:L$SexM:L$FoodQ          -0.504 0.614487
## L$PopulationPC:L$GenotypeFFD19:L$SexM:L$FoodQ               NA       NA
## L$PopulationFFD:L$GenotypeFFD2:L$SexM:L$FoodQ           -0.620 0.535503
## L$PopulationPC:L$GenotypeFFD2:L$SexM:L$FoodQ                NA       NA
## L$PopulationFFD:L$GenotypeFFD22:L$SexM:L$FoodQ          -1.004 0.315432
## L$PopulationPC:L$GenotypeFFD22:L$SexM:L$FoodQ               NA       NA
## L$PopulationFFD:L$GenotypeFFD23:L$SexM:L$FoodQ          -0.156 0.875842
## L$PopulationPC:L$GenotypeFFD23:L$SexM:L$FoodQ               NA       NA
## L$PopulationFFD:L$GenotypeFFD29:L$SexM:L$FoodQ           1.255 0.209484
## L$PopulationPC:L$GenotypeFFD29:L$SexM:L$FoodQ               NA       NA
## L$PopulationFFD:L$GenotypeFFD4:L$SexM:L$FoodQ           -0.721 0.471141
## L$PopulationPC:L$GenotypeFFD4:L$SexM:L$FoodQ                NA       NA
## L$PopulationFFD:L$GenotypeFFD6:L$SexM:L$FoodQ           -0.890 0.373697
## L$PopulationPC:L$GenotypeFFD6:L$SexM:L$FoodQ                NA       NA
## L$PopulationFFD:L$GenotypePC113:L$SexM:L$FoodQ              NA       NA
## L$PopulationPC:L$GenotypePC113:L$SexM:L$FoodQ            0.177 0.859134
## L$PopulationFFD:L$GenotypePC114:L$SexM:L$FoodQ              NA       NA
## L$PopulationPC:L$GenotypePC114:L$SexM:L$FoodQ           -0.291 0.770793
## L$PopulationFFD:L$GenotypePC12:L$SexM:L$FoodQ               NA       NA
## L$PopulationPC:L$GenotypePC12:L$SexM:L$FoodQ             1.400 0.161674
## L$PopulationFFD:L$GenotypePC136:L$SexM:L$FoodQ              NA       NA
## L$PopulationPC:L$GenotypePC136:L$SexM:L$FoodQ            0.671 0.502438
## L$PopulationFFD:L$GenotypePC141:L$SexM:L$FoodQ              NA       NA
## L$PopulationPC:L$GenotypePC141:L$SexM:L$FoodQ            1.357 0.174824
## L$PopulationFFD:L$GenotypePC155:L$SexM:L$FoodQ              NA       NA
## L$PopulationPC:L$GenotypePC155:L$SexM:L$FoodQ            0.681 0.495821
## L$PopulationFFD:L$GenotypePC167:L$SexM:L$FoodQ              NA       NA
## L$PopulationPC:L$GenotypePC167:L$SexM:L$FoodQ            0.526 0.599122
## L$PopulationFFD:L$GenotypePC189:L$SexM:L$FoodQ              NA       NA
## L$PopulationPC:L$GenotypePC189:L$SexM:L$FoodQ            1.309 0.190771
## L$PopulationFFD:L$GenotypePC200:L$SexM:L$FoodQ              NA       NA
## L$PopulationPC:L$GenotypePC200:L$SexM:L$FoodQ            0.494 0.621485
## L$PopulationFFD:L$GenotypePC85:L$SexM:L$FoodQ               NA       NA
## L$PopulationPC:L$GenotypePC85:L$SexM:L$FoodQ                NA       NA
## L$PopulationFFD:L$GenotypeFFD14:L$TempLow:L$FoodQ       -0.489 0.624931
## L$PopulationPC:L$GenotypeFFD14:L$TempLow:L$FoodQ            NA       NA
## L$PopulationFFD:L$GenotypeFFD16:L$TempLow:L$FoodQ       -1.190 0.234025
## L$PopulationPC:L$GenotypeFFD16:L$TempLow:L$FoodQ            NA       NA
## L$PopulationFFD:L$GenotypeFFD19:L$TempLow:L$FoodQ       -1.995 0.046080 *
## L$PopulationPC:L$GenotypeFFD19:L$TempLow:L$FoodQ            NA       NA
## L$PopulationFFD:L$GenotypeFFD2:L$TempLow:L$FoodQ        -2.639 0.008347 **
## L$PopulationPC:L$GenotypeFFD2:L$TempLow:L$FoodQ             NA       NA
## L$PopulationFFD:L$GenotypeFFD22:L$TempLow:L$FoodQ       -2.023 0.043168 *
## L$PopulationPC:L$GenotypeFFD22:L$TempLow:L$FoodQ            NA       NA
## L$PopulationFFD:L$GenotypeFFD23:L$TempLow:L$FoodQ        0.785 0.432596
## L$PopulationPC:L$GenotypeFFD23:L$TempLow:L$FoodQ            NA       NA
## L$PopulationFFD:L$GenotypeFFD29:L$TempLow:L$FoodQ        1.653 0.098420 .
## L$PopulationPC:L$GenotypeFFD29:L$TempLow:L$FoodQ            NA       NA
## L$PopulationFFD:L$GenotypeFFD4:L$TempLow:L$FoodQ        -3.172 0.001526 **
## L$PopulationPC:L$GenotypeFFD4:L$TempLow:L$FoodQ             NA       NA
## L$PopulationFFD:L$GenotypeFFD6:L$TempLow:L$FoodQ         0.740 0.459542
## L$PopulationPC:L$GenotypeFFD6:L$TempLow:L$FoodQ             NA       NA
```

```
## L$PopulationFFD:L$GenotypePC113:L$TempLow:L$FoodQ            NA        NA
## L$PopulationPC:L$GenotypePC113:L$TempLow:L$FoodQ          0.757 0.448890
## L$PopulationFFD:L$GenotypePC114:L$TempLow:L$FoodQ            NA        NA
## L$PopulationPC:L$GenotypePC114:L$TempLow:L$FoodQ          7.626 2.98e-14 ***
## L$PopulationFFD:L$GenotypePC12:L$TempLow:L$FoodQ             NA        NA
## L$PopulationPC:L$GenotypePC12:L$TempLow:L$FoodQ           2.262 0.023772 *
## L$PopulationFFD:L$GenotypePC136:L$TempLow:L$FoodQ            NA        NA
## L$PopulationPC:L$GenotypePC136:L$TempLow:L$FoodQ         -0.622 0.534304
## L$PopulationFFD:L$GenotypePC141:L$TempLow:L$FoodQ            NA        NA
## L$PopulationPC:L$GenotypePC141:L$TempLow:L$FoodQ          0.943 0.345664
## L$PopulationFFD:L$GenotypePC155:L$TempLow:L$FoodQ            NA        NA
## L$PopulationPC:L$GenotypePC155:L$TempLow:L$FoodQ          3.477 0.000512 ***
## L$PopulationFFD:L$GenotypePC167:L$TempLow:L$FoodQ            NA        NA
## L$PopulationPC:L$GenotypePC167:L$TempLow:L$FoodQ          7.791 8.37e-15 ***
## L$PopulationFFD:L$GenotypePC189:L$TempLow:L$FoodQ            NA        NA
## L$PopulationPC:L$GenotypePC189:L$TempLow:L$FoodQ          1.256 0.209297
## L$PopulationFFD:L$GenotypePC200:L$TempLow:L$FoodQ            NA        NA
## L$PopulationPC:L$GenotypePC200:L$TempLow:L$FoodQ         -3.866 0.000112 ***
## L$PopulationFFD:L$GenotypePC85:L$TempLow:L$FoodQ             NA        NA
## L$PopulationPC:L$GenotypePC85:L$TempLow:L$FoodQ              NA        NA
## L$PopulationPC:L$SexM:L$TempLow:L$FoodQ                  -0.028 0.977325
## L$PopulationFFD:L$GenotypeFFD14:L$SexM:L$TempLow:L$FoodQ  -1.052 0.292995
## L$PopulationPC:L$GenotypeFFD14:L$SexM:L$TempLow:L$FoodQ      NA        NA
## L$PopulationFFD:L$GenotypeFFD16:L$SexM:L$TempLow:L$FoodQ  -1.012 0.311553
## L$PopulationPC:L$GenotypeFFD16:L$SexM:L$TempLow:L$FoodQ      NA        NA
## L$PopulationFFD:L$GenotypeFFD19:L$SexM:L$TempLow:L$FoodQ  -0.534 0.593204
## L$PopulationPC:L$GenotypeFFD19:L$SexM:L$TempLow:L$FoodQ      NA        NA
## L$PopulationFFD:L$GenotypeFFD2:L$SexM:L$TempLow:L$FoodQ   -0.713 0.475977
## L$PopulationPC:L$GenotypeFFD2:L$SexM:L$TempLow:L$FoodQ       NA        NA
## L$PopulationFFD:L$GenotypeFFD22:L$SexM:L$TempLow:L$FoodQ  -0.666 0.505191
## L$PopulationPC:L$GenotypeFFD22:L$SexM:L$TempLow:L$FoodQ      NA        NA
## L$PopulationFFD:L$GenotypeFFD23:L$SexM:L$TempLow:L$FoodQ  -1.308 0.190795
## L$PopulationPC:L$GenotypeFFD23:L$SexM:L$TempLow:L$FoodQ      NA        NA
## L$PopulationFFD:L$GenotypeFFD29:L$SexM:L$TempLow:L$FoodQ  -2.542 0.011055 *
## L$PopulationPC:L$GenotypeFFD29:L$SexM:L$TempLow:L$FoodQ      NA        NA
## L$PopulationFFD:L$GenotypeFFD4:L$SexM:L$TempLow:L$FoodQ    0.580 0.562225
## L$PopulationPC:L$GenotypeFFD4:L$SexM:L$TempLow:L$FoodQ       NA        NA
## L$PopulationFFD:L$GenotypeFFD6:L$SexM:L$TempLow:L$FoodQ   -0.472 0.636893
## L$PopulationPC:L$GenotypeFFD6:L$SexM:L$TempLow:L$FoodQ       NA        NA
## L$PopulationFFD:L$GenotypePC113:L$SexM:L$TempLow:L$FoodQ      NA        NA
## L$PopulationPC:L$GenotypePC113:L$SexM:L$TempLow:L$FoodQ   -2.044 0.041023 *
## L$PopulationFFD:L$GenotypePC114:L$SexM:L$TempLow:L$FoodQ      NA        NA
## L$PopulationPC:L$GenotypePC114:L$SexM:L$TempLow:L$FoodQ   -1.606 0.108330
## L$PopulationFFD:L$GenotypePC12:L$SexM:L$TempLow:L$FoodQ       NA        NA
## L$PopulationPC:L$GenotypePC12:L$SexM:L$TempLow:L$FoodQ    -1.032 0.302355
## L$PopulationFFD:L$GenotypePC136:L$SexM:L$TempLow:L$FoodQ      NA        NA
## L$PopulationPC:L$GenotypePC136:L$SexM:L$TempLow:L$FoodQ    0.187 0.851843
## L$PopulationFFD:L$GenotypePC141:L$SexM:L$TempLow:L$FoodQ      NA        NA
## L$PopulationPC:L$GenotypePC141:L$SexM:L$TempLow:L$FoodQ   -1.453 0.146162
## L$PopulationFFD:L$GenotypePC155:L$SexM:L$TempLow:L$FoodQ      NA        NA
## L$PopulationPC:L$GenotypePC155:L$SexM:L$TempLow:L$FoodQ   -0.416 0.677308
## L$PopulationFFD:L$GenotypePC167:L$SexM:L$TempLow:L$FoodQ      NA        NA
## L$PopulationPC:L$GenotypePC167:L$SexM:L$TempLow:L$FoodQ      NA        NA
## L$PopulationFFD:L$GenotypePC189:L$SexM:L$TempLow:L$FoodQ      NA        NA
```

```
## L$PopulationPC:L$GenotypePC189:L$SexM:L$TempLow:L$FoodQ    0.507 0.612354
## L$PopulationFFD:L$GenotypePC200:L$SexM:L$TempLow:L$FoodQ      NA       NA
## L$PopulationPC:L$GenotypePC200:L$SexM:L$TempLow:L$FoodQ    0.010 0.992167
## L$PopulationFFD:L$GenotypePC85:L$SexM:L$TempLow:L$FoodQ       NA       NA
## L$PopulationPC:L$GenotypePC85:L$SexM:L$TempLow:L$FoodQ        NA       NA
## ---
## Signif. codes:  0 '***' 0.001 '**' 0.01 '*' 0.05 '.' 0.1 ' ' 1
##
## Residual standard error: 0.04949 on 4150 degrees of freedom
## Multiple R-squared:  0.8168, Adjusted R-squared:  0.8099
## F-statistic: 117.9 on 157 and 4150 DF,  p-value: < 2.2e-16
```

###Next we are going to do the same sets of analyses looking at the femur data.

```
y<-read.table("~/Dropbox/Didem_data/Size/Femursize.txt",header=TRUE)
summary(y)
```

```
##     Genotype    Population   Temp      Sex      Food      Cat
##  PC85   : 229   FFD:1854   High:1774   F:1971   F:1891   21F:973
##  PC141  : 224   PC :1749   Low :1829   M:1632   Q:1712   21Q:856
##  PC155  : 217                                            25F:918
##  FFD6   : 204                                            25Q:856
##  FFD29  : 202
##  FFD22  : 201
##  (Other):2326
##      Length
##  Min.   :0.3752
##  1st Qu.:0.5285
##  Median :0.5589
##  Mean   :0.5553
##  3rd Qu.:0.5860
##  Max.   :0.6665
##
```

##First look at the population level patterns at different temperatures for the fully fed group.

```
High_F<-subset(y,y$Temp=="High"&y$Food=="F")

Low_F<-subset(y,y$Temp=="Low"&y$Food=="F")

ggplot(data=High_F, aes(Population,log(Length))) + geom_boxplot(aes(fill=Sex),width=0.7)
+ theme_tufte()  + ggtitle("High_Fed") + theme(axis.text.x = element_text(angle = 90, hj
ust = 1))
```

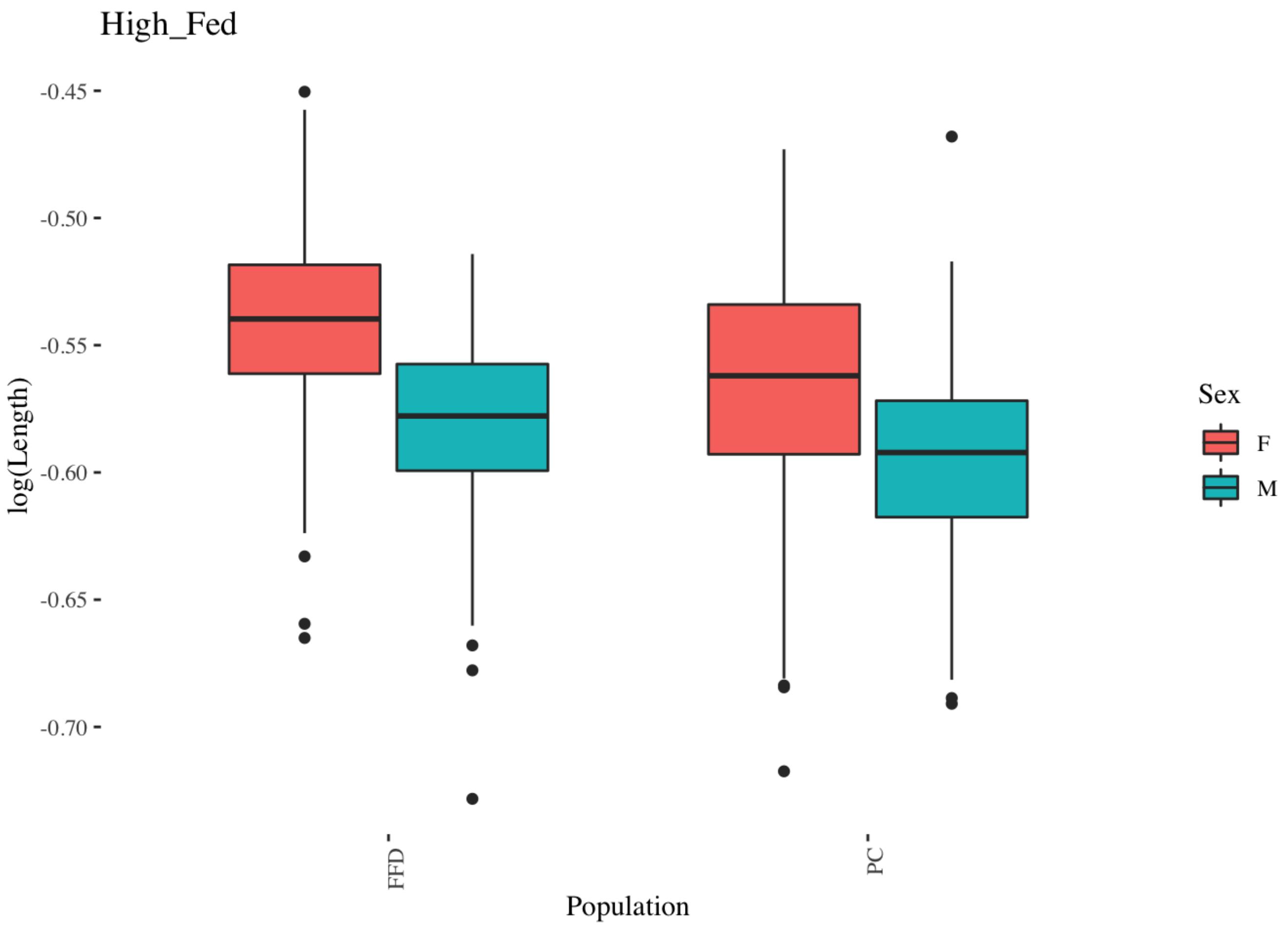


```
ggplot(data=Low_F, aes(Population,log(Length))) + geom_boxplot(aes(fill=Sex),width=0.7)
 + theme_tufte()  + ggtitle("Low_Fed") + theme(axis.text.x = element_text(angle = 90, hj
ust = 1))
```

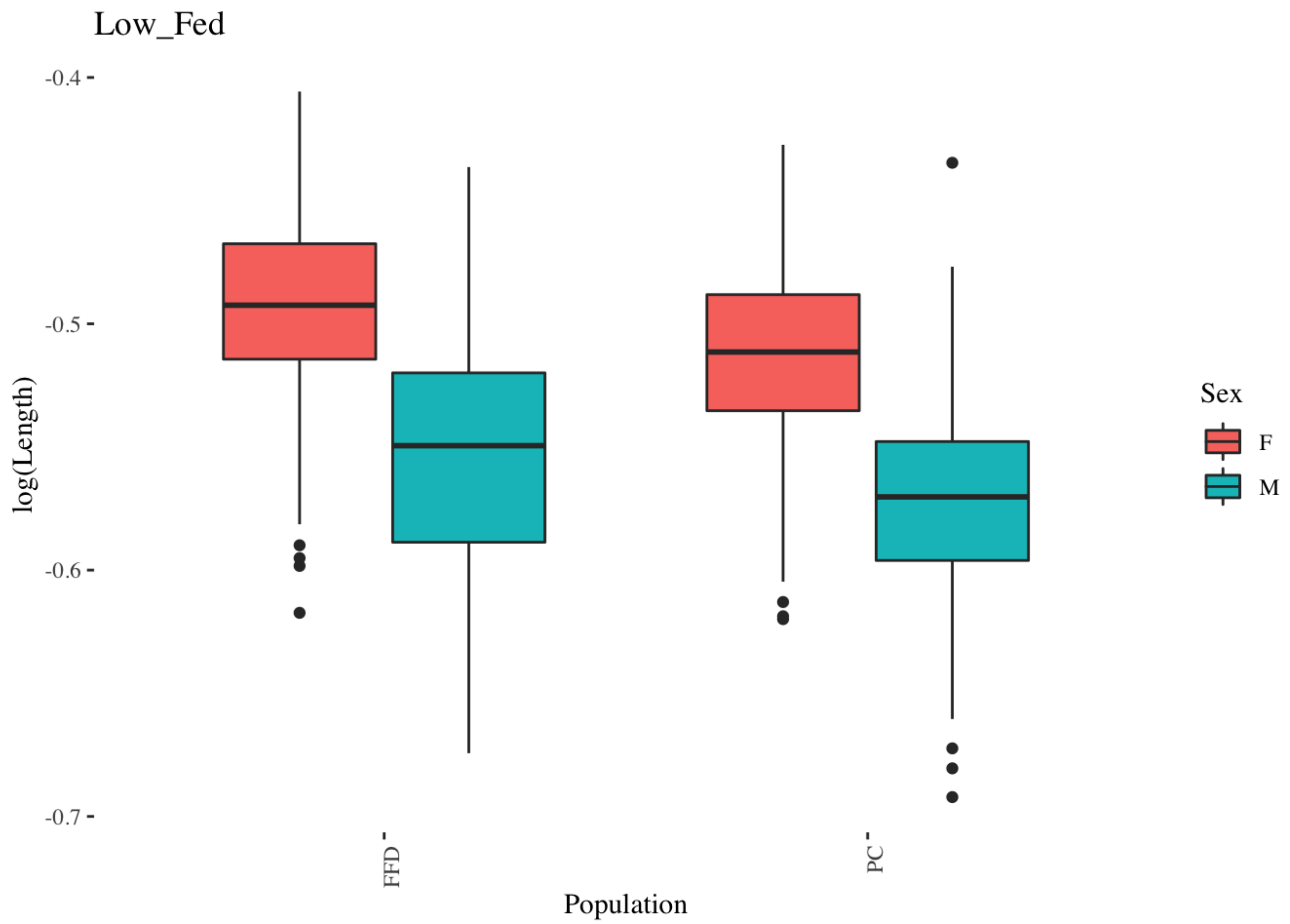

###Next repeat this analysis with the quarter food group.

```
High_Q<-subset(y,y$Temp=="High"&y$Food=="Q")

Low_Q<-subset(y,y$Temp=="Low"&y$Food=="Q")

ggplot(data=High_Q, aes(Population,log(Length))) + geom_boxplot(aes(fill=Sex),width=0.7)
+ theme_tufte() + ggtitle("High_Quarter") + theme(axis.text.x = element_text(angle = 90,
hjust = 1))
```

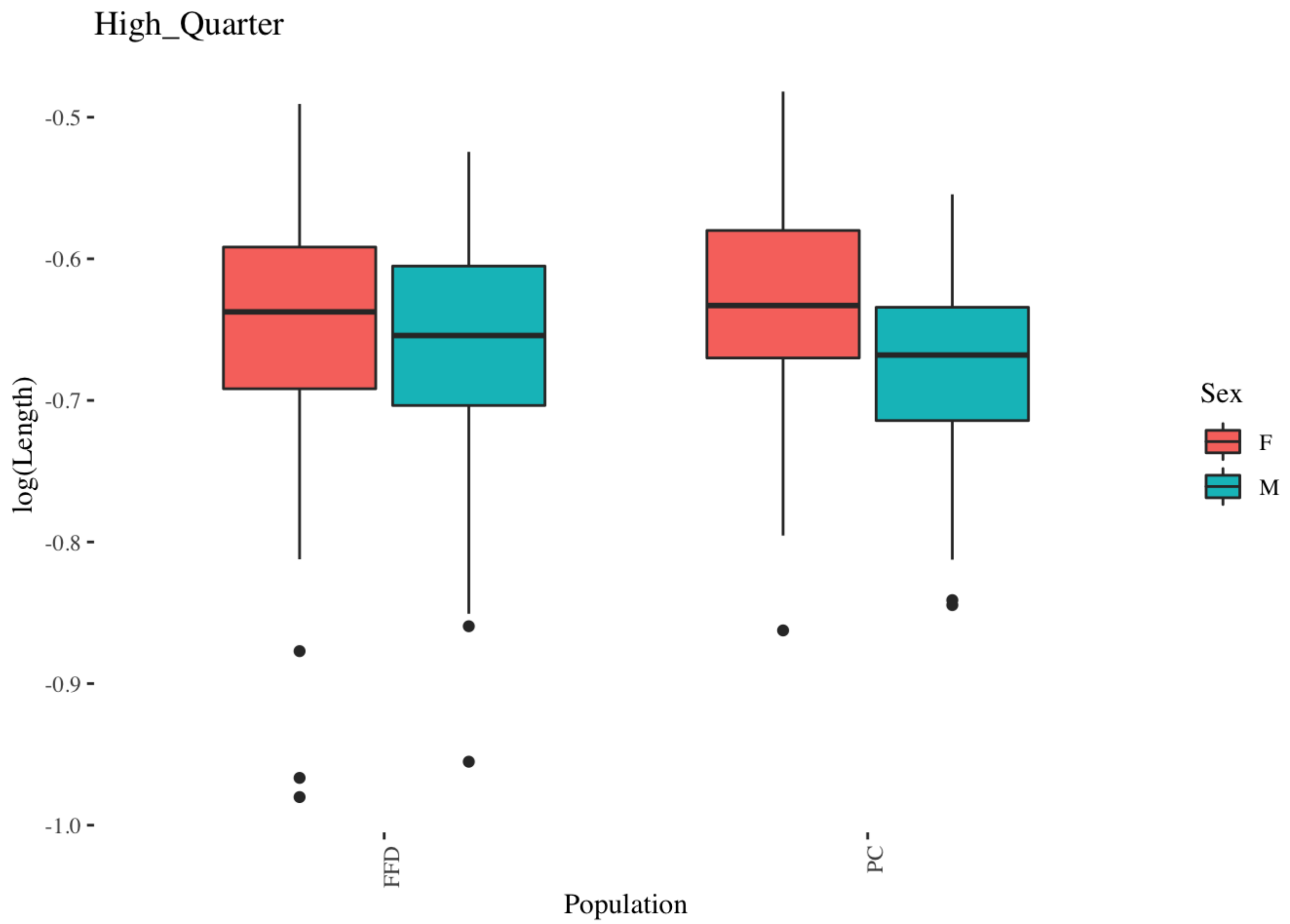


```
ggplot(data=Low_Q, aes(Population,log(Length))) + geom_boxplot(aes(fill=Sex),width=0.7)
 + theme_tufte()  + ggtitle("Low_Quarter") + theme(axis.text.x = element_text(angle = 90
, hjust = 1))
```

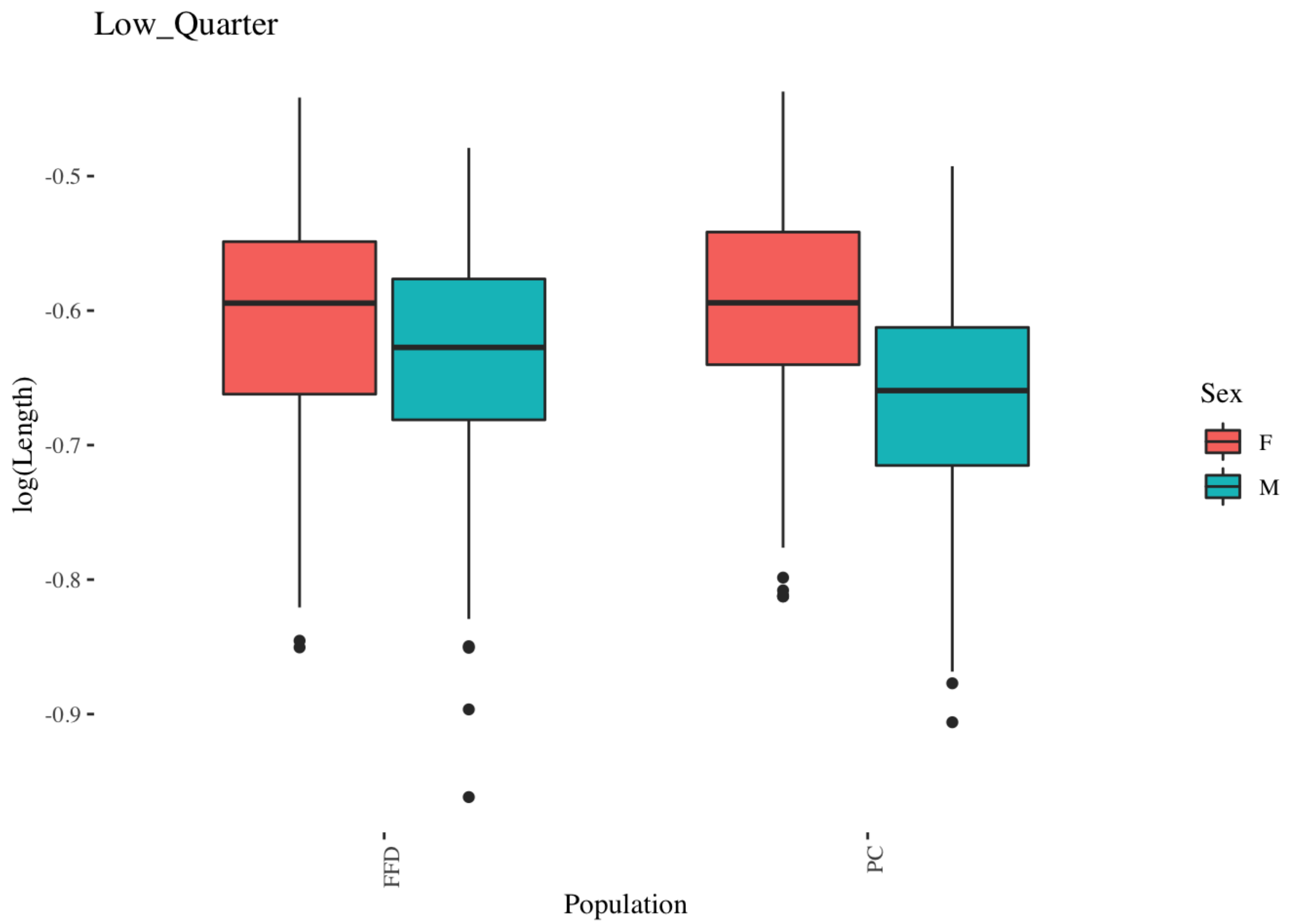

###Next we ware looking at the differences between temperatures for fully fed individuals.

```
fFF<-subset(y,y$Food=="F"&y$Sex=="F")

ggplot(data=fFF, aes(Population,log(Length))) + geom_boxplot(aes(fill=Temp),width=0.7) +
theme_tufte()  + ggtitle("Females Fed") + theme(axis.text.x = element_text(angle = 90, h
just = 1))
```

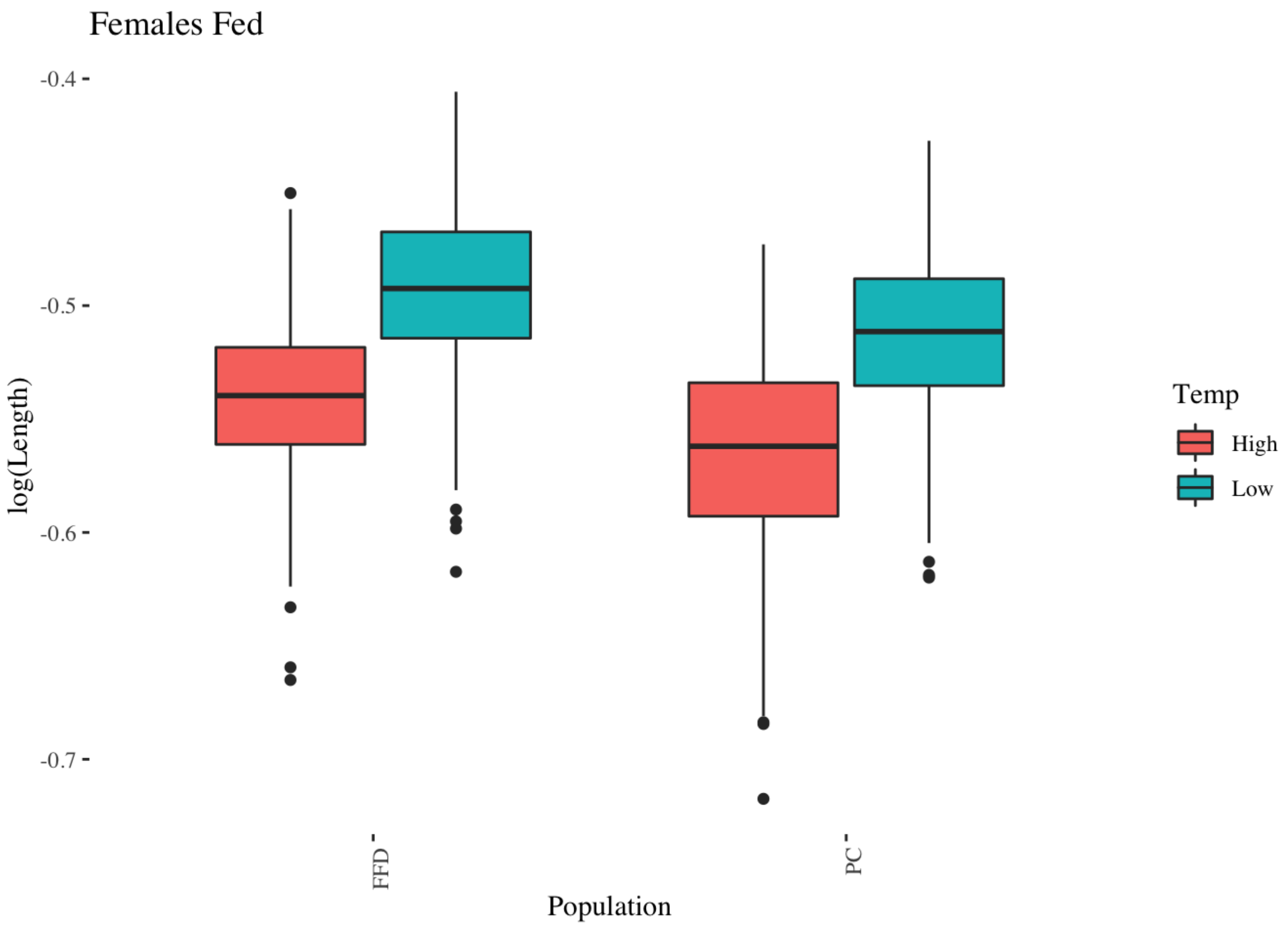


```
fMF<-subset(y,y$Food=="F"&y$Sex=="M")

ggplot(data=fMF, aes(Population,log(Length))) + geom_boxplot(aes(fill=Temp),width=0.7) +
theme_tufte()  + ggtitle("Males Fed") + theme(axis.text.x = element_text(angle = 90, hju
st = 1))
```

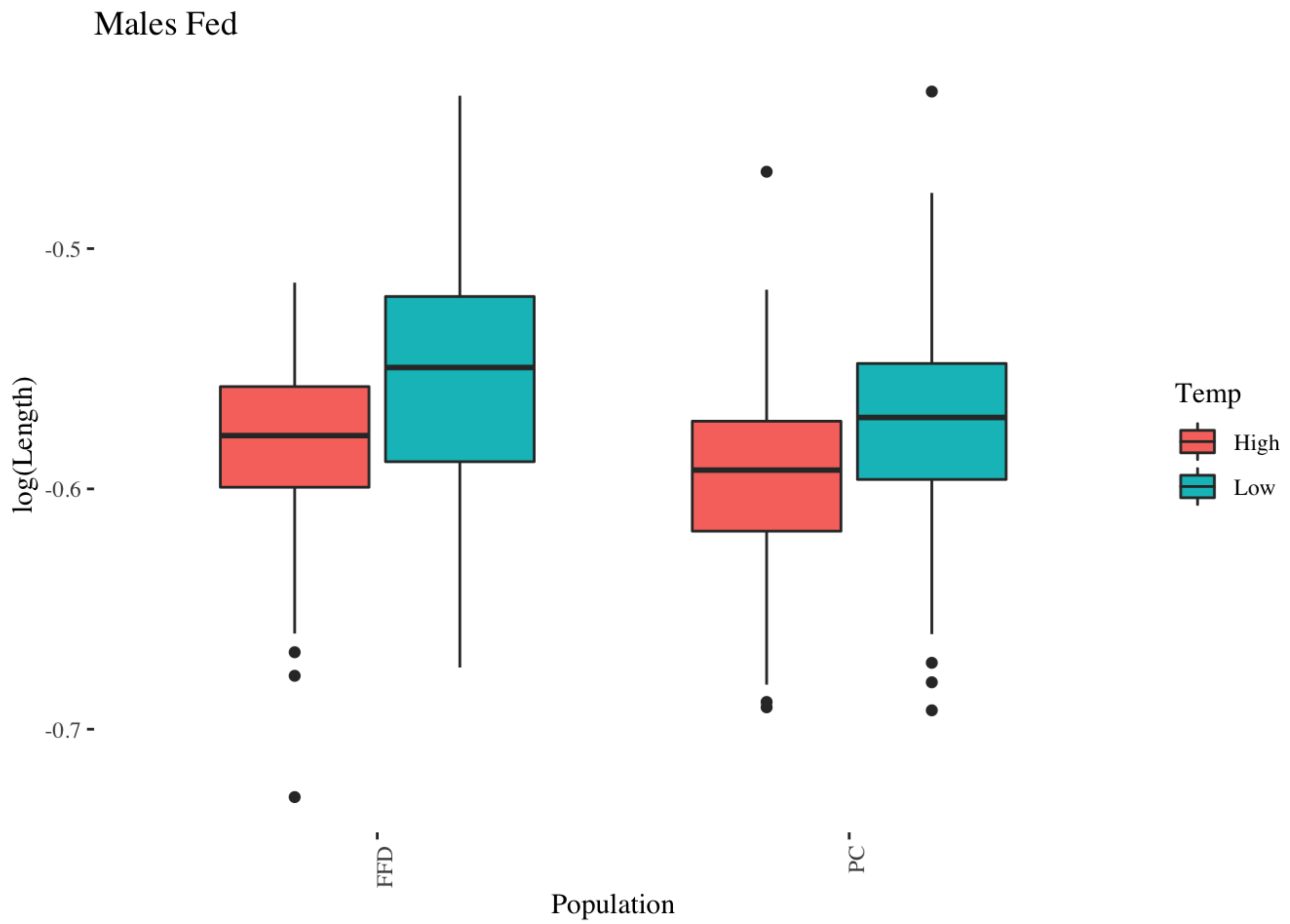

###Next we ware looking at the differences between temperatures for quarter fed individuals.

```
fFQ<-subset(y,y$Food=="Q"&y$Sex=="F")

ggplot(data=fFQ, aes(Population,log(Length))) + geom_boxplot(aes(fill=Temp),width=0.7) +
theme_tufte()  + ggtitle("Females Quarter") + theme(axis.text.x = element_text(angle = 9
0, hjust = 1))
```

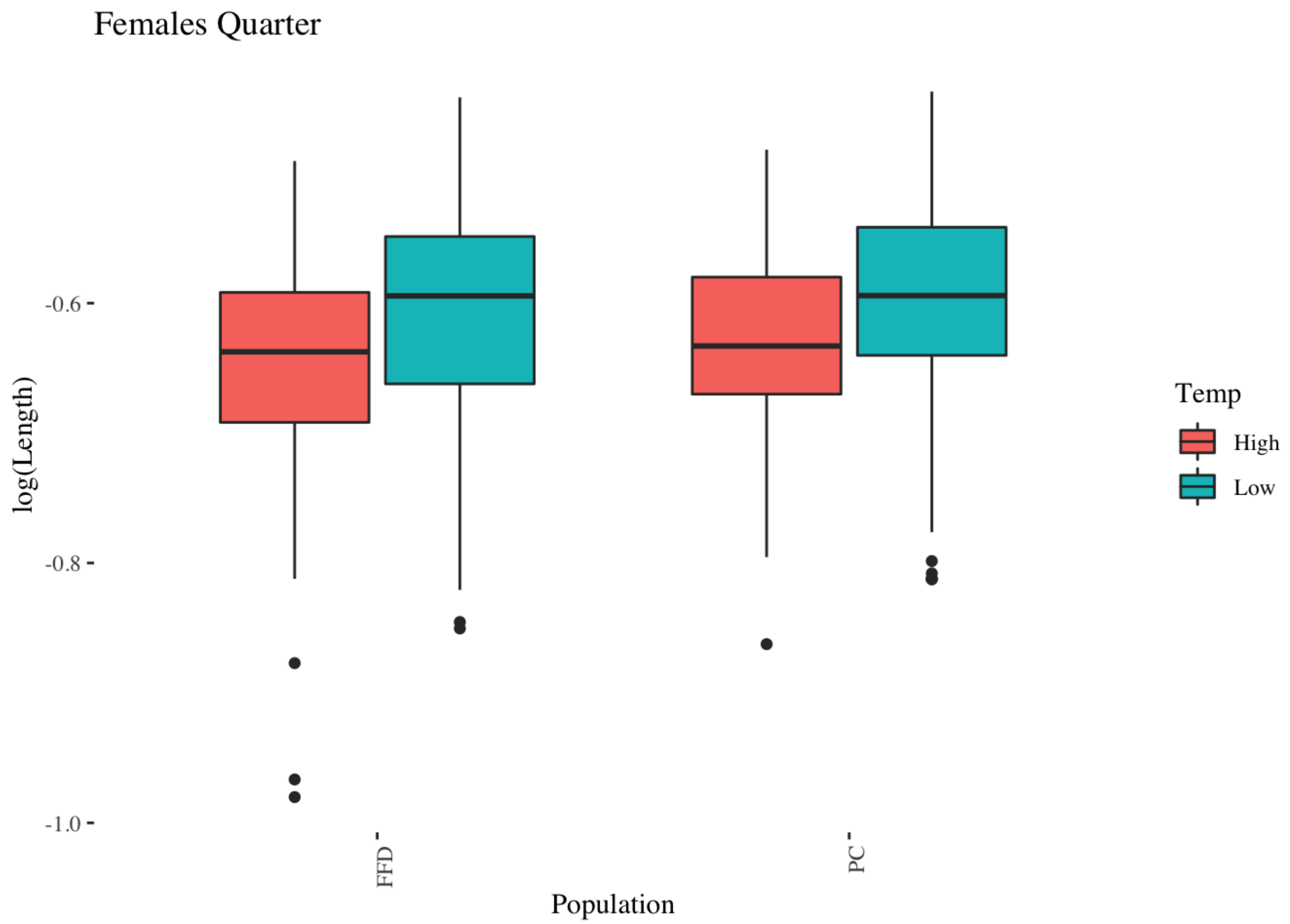


```
fMQ<-subset(y,y$Food=="Q"&y$Sex=="M")

ggplot(data=fMQ, aes(Population,log(Length))) + geom_boxplot(aes(fill=Temp),width=0.7) +
theme_tufte()  + ggtitle("Males Quarter") + theme(axis.text.x = element_text(angle = 90,
hjust = 1))
```

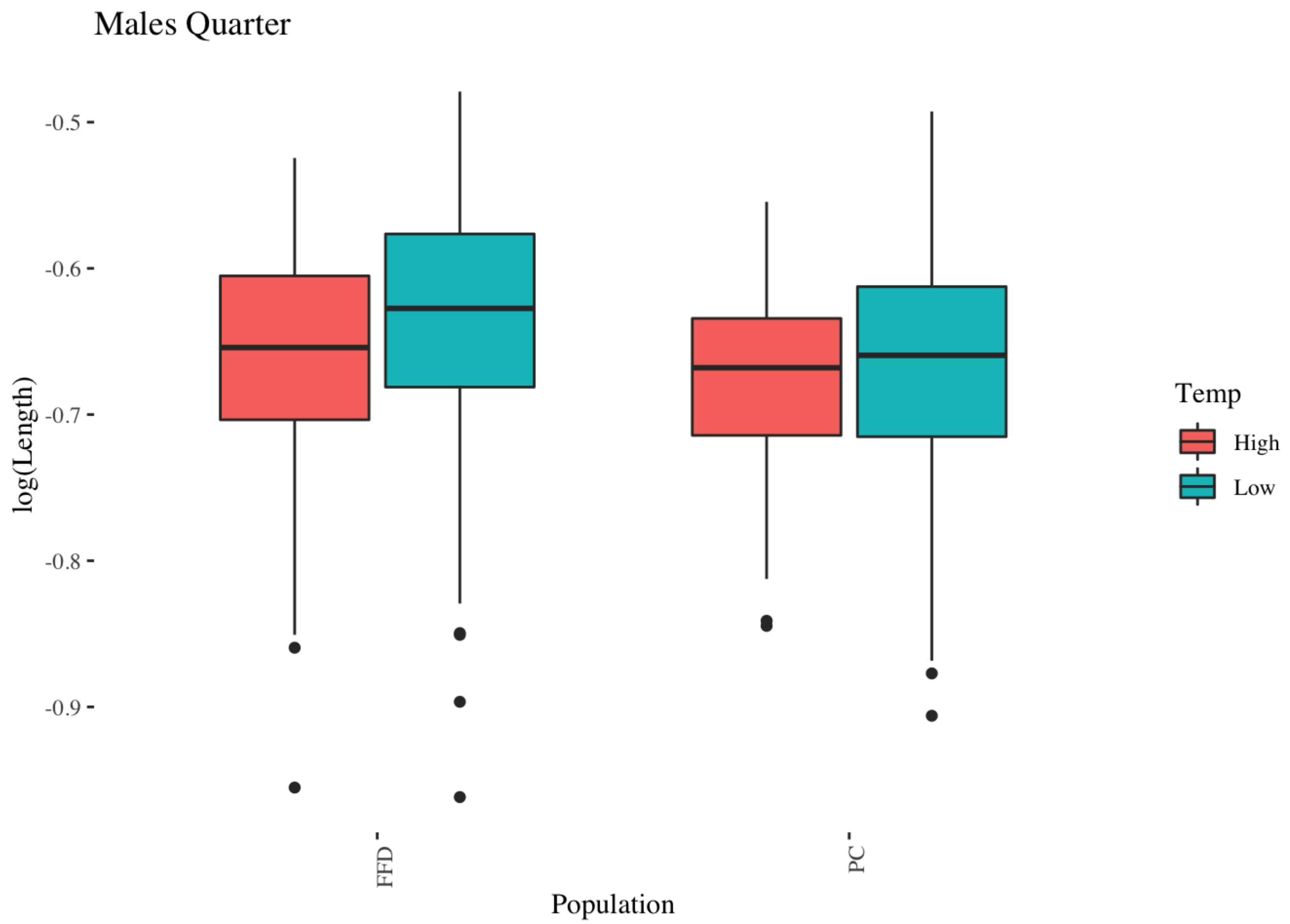

###Do global ANOVA for femur data

```
ModfGlobal<-lm(log(y$Length) ~ y$Population*y$Population:y$Genotype*y$Sex*y$Temp*y$Food)

print(anova(ModfGlobal))
```

```
## Analysis of Variance Table
##
## Response: log(y$Length)
##                                              Df Sum Sq Mean Sq   F value
## y$Population                                  1 0.0972  0.0972   35.9027
## y$Sex                                         1 2.0156  2.0156  744.6245
## y$Temp                                        1 1.0758  1.0758  397.4253
## y$Food                                        1 7.0679  7.0679 2611.0502
## y$Population:y$Genotype                      18 1.0145  0.0564   20.8211
## y$Population:y$Sex                            1 0.0242  0.0242    8.9272
## y$Population:y$Temp                           1 0.0019  0.0019    0.7068
## y$Sex:y$Temp                                  1 0.0561  0.0561   20.7355
## y$Population:y$Food                           1 0.0581  0.0581   21.4787
## y$Sex:y$Food                                  1 0.0216  0.0216    7.9952
## y$Temp:y$Food                                 1 0.0220  0.0220    8.1349
## y$Population:y$Genotype:y$Sex                17 0.1840  0.0108    3.9984
## y$Population:y$Genotype:y$Temp               17 0.7632  0.0449   16.5848
## y$Population:y$Sex:y$Temp                     1 0.0001  0.0001    0.0420
## y$Population:y$Genotype:y$Food               18 0.3981  0.0221    8.1709
## y$Population:y$Sex:y$Food                     1 0.0481  0.0481   17.7853
## y$Population:y$Temp:y$Food                    1 0.0020  0.0020    0.7518
## y$Sex:y$Temp:y$Food                           1 0.0003  0.0003    0.1068
## y$Population:y$Genotype:y$Sex:y$Temp         15 0.0652  0.0043    1.6053
## y$Population:y$Genotype:y$Sex:y$Food         16 0.2340  0.0146    5.4027
## y$Population:y$Genotype:y$Temp:y$Food        15 0.4473  0.0298   11.0161
## y$Population:y$Sex:y$Temp:y$Food              1 0.0079  0.0079    2.9273
## y$Population:y$Genotype:y$Sex:y$Temp:y$Food   7 0.0158  0.0023    0.8356
## Residuals                                  3464 9.3768  0.0027
##                                               Pr(>F)
## y$Population                               2.287e-09 ***
## y$Sex                                      < 2.2e-16 ***
## y$Temp                                     < 2.2e-16 ***
## y$Food                                     < 2.2e-16 ***
## y$Population:y$Genotype                    < 2.2e-16 ***
## y$Population:y$Sex                          0.002829 **
## y$Population:y$Temp                         0.400580
## y$Sex:y$Temp                               5.454e-06 ***
## y$Population:y$Food                        3.710e-06 ***
## y$Sex:y$Food                                0.004717 **
## y$Temp:y$Food                               0.004368 **
## y$Population:y$Genotype:y$Sex              5.877e-08 ***
## y$Population:y$Genotype:y$Temp             < 2.2e-16 ***
## y$Population:y$Sex:y$Temp                   0.837578
## y$Population:y$Genotype:y$Food             < 2.2e-16 ***
## y$Population:y$Sex:y$Food                  2.536e-05 ***
## y$Population:y$Temp:y$Food                  0.385952
## y$Sex:y$Temp:y$Food                         0.743886
## y$Population:y$Genotype:y$Sex:y$Temp        0.064391 .
## y$Population:y$Genotype:y$Sex:y$Food       1.635e-11 ***
## y$Population:y$Genotype:y$Temp:y$Food      < 2.2e-16 ***
## y$Population:y$Sex:y$Temp:y$Food            0.087184 .
## y$Population:y$Genotype:y$Sex:y$Temp:y$Food  0.557525
## Residuals
```

```
## ---
## Signif. codes:  0 '***' 0.001 '**' 0.01 '*' 0.05 '.' 0.1 ' ' 1
```

```
print(summary(ModfGlobal))
```

```
##
## Call:
## lm(formula = log(y$Length) ~ y$Population * y$Population:y$Genotype *
##     y$Sex * y$Temp * y$Food)
##
## Residuals:
##       Min        1Q    Median        3Q       Max
## -0.297619 -0.026702  0.003273  0.030467  0.224579
##
## Coefficients: (181 not defined because of singularities)
##                                                          Estimate Std. Error
## (Intercept)                                            -0.5382635  0.0110924
## y$PopulationPC                                         -0.0198851  0.0146038
## y$SexM                                                 -0.0436224  0.0155156
## y$TempLow                                               0.0143233  0.0153568
## y$FoodQ                                                -0.0828452  0.0152092
## y$PopulationFFD:y$GenotypeFFD14                         0.0016681  0.0153568
## y$PopulationPC:y$GenotypeFFD14                                 NA         NA
## y$PopulationFFD:y$GenotypeFFD16                        -0.0176767  0.0152092
## y$PopulationPC:y$GenotypeFFD16                                 NA         NA
## y$PopulationFFD:y$GenotypeFFD19                         0.0151622  0.0156870
## y$PopulationPC:y$GenotypeFFD19                                 NA         NA
## y$PopulationFFD:y$GenotypeFFD2                          0.0048641  0.0155156
## y$PopulationPC:y$GenotypeFFD2                                  NA         NA
## y$PopulationFFD:y$GenotypeFFD22                        -0.0273845  0.0155156
## y$PopulationPC:y$GenotypeFFD22                                 NA         NA
## y$PopulationFFD:y$GenotypeFFD23                         0.0077333  0.0150716
## y$PopulationPC:y$GenotypeFFD23                                 NA         NA
## y$PopulationFFD:y$GenotypeFFD29                         0.0033352  0.0150716
## y$PopulationPC:y$GenotypeFFD29                                 NA         NA
## y$PopulationFFD:y$GenotypeFFD4                          0.0047665  0.0148229
## y$PopulationPC:y$GenotypeFFD4                                  NA         NA
## y$PopulationFFD:y$GenotypeFFD6                         -0.0209190  0.0152092
## y$PopulationPC:y$GenotypeFFD6                                  NA         NA
## y$PopulationFFD:y$GenotypePC113                                NA         NA
## y$PopulationPC:y$GenotypePC113                         -0.0679866  0.0120613
## y$PopulationFFD:y$GenotypePC114                                NA         NA
## y$PopulationPC:y$GenotypePC114                          0.0009973  0.0139407
## y$PopulationFFD:y$GenotypePC12                                 NA         NA
## y$PopulationPC:y$GenotypePC12                          -0.0117256  0.0138017
## y$PopulationFFD:y$GenotypePC136                                NA         NA
## y$PopulationPC:y$GenotypePC136                         -0.0215282  0.0148031
## y$PopulationFFD:y$GenotypePC141                                NA         NA
## y$PopulationPC:y$GenotypePC141                          0.0196626  0.0134336
## y$PopulationFFD:y$GenotypePC155                                NA         NA
## y$PopulationPC:y$GenotypePC155                          0.0145126  0.0134336
## y$PopulationFFD:y$GenotypePC167                                NA         NA
## y$PopulationPC:y$GenotypePC167                          0.0192473  0.0142485
## y$PopulationFFD:y$GenotypePC189                                NA         NA
## y$PopulationPC:y$GenotypePC189                          0.0143087  0.0140893
## y$PopulationFFD:y$GenotypePC200                                NA         NA
## y$PopulationPC:y$GenotypePC200                          0.0358955  0.0121899
## y$PopulationFFD:y$GenotypePC85                                 NA         NA
```

```
## y$PopulationPC:y$GenotypePC85                              NA         NA
## y$PopulationPC:y$SexM                               0.0100942  0.0205230
## y$PopulationPC:y$TempLow                            0.0056015  0.0206474
## y$SexM:y$TempLow                                   -0.0031689  0.0215942
## y$PopulationPC:y$FoodQ                             -0.0095530  0.0208408
## y$SexM:y$FoodQ                                      0.0108587  0.0216033
## y$TempLow:y$FoodQ                                   0.0025574  0.0214895
## y$PopulationFFD:y$GenotypeFFD14:y$SexM              0.0014695  0.0214895
## y$PopulationPC:y$GenotypeFFD14:y$SexM                      NA         NA
## y$PopulationFFD:y$GenotypeFFD16:y$SexM              0.0212501  0.0218612
## y$PopulationPC:y$GenotypeFFD16:y$SexM                      NA         NA
## y$PopulationFFD:y$GenotypeFFD19:y$SexM             -0.0123924  0.0365467
## y$PopulationPC:y$GenotypeFFD19:y$SexM                      NA         NA
## y$PopulationFFD:y$GenotypeFFD2:y$SexM              -0.0149821  0.0217075
## y$PopulationPC:y$GenotypeFFD2:y$SexM                       NA         NA
## y$PopulationFFD:y$GenotypeFFD22:y$SexM              0.0432084  0.0217075
## y$PopulationPC:y$GenotypeFFD22:y$SexM                      NA         NA
## y$PopulationFFD:y$GenotypeFFD23:y$SexM             -0.0018897  0.0212867
## y$PopulationPC:y$GenotypeFFD23:y$SexM                      NA         NA
## y$PopulationFFD:y$GenotypeFFD29:y$SexM             -0.0092350  0.0217657
## y$PopulationPC:y$GenotypeFFD29:y$SexM                      NA         NA
## y$PopulationFFD:y$GenotypeFFD4:y$SexM               0.0193239  0.0209205
## y$PopulationPC:y$GenotypeFFD4:y$SexM                       NA         NA
## y$PopulationFFD:y$GenotypeFFD6:y$SexM              -0.0013278  0.0208231
## y$PopulationPC:y$GenotypeFFD6:y$SexM                       NA         NA
## y$PopulationFFD:y$GenotypePC113:y$SexM                     NA         NA
## y$PopulationPC:y$GenotypePC113:y$SexM               0.1334158  0.0276653
## y$PopulationFFD:y$GenotypePC114:y$SexM                     NA         NA
## y$PopulationPC:y$GenotypePC114:y$SexM              -0.0153220  0.0193599
## y$PopulationFFD:y$GenotypePC12:y$SexM                      NA         NA
## y$PopulationPC:y$GenotypePC12:y$SexM               -0.0186405  0.0202390
## y$PopulationFFD:y$GenotypePC136:y$SexM                     NA         NA
## y$PopulationPC:y$GenotypePC136:y$SexM               0.0083092  0.0205463
## y$PopulationFFD:y$GenotypePC141:y$SexM                     NA         NA
## y$PopulationPC:y$GenotypePC141:y$SexM              -0.0124477  0.0189212
## y$PopulationFFD:y$GenotypePC155:y$SexM                     NA         NA
## y$PopulationPC:y$GenotypePC155:y$SexM               0.0075498  0.0190797
## y$PopulationFFD:y$GenotypePC167:y$SexM                     NA         NA
## y$PopulationPC:y$GenotypePC167:y$SexM              -0.0304593  0.0200381
## y$PopulationFFD:y$GenotypePC189:y$SexM                     NA         NA
## y$PopulationPC:y$GenotypePC189:y$SexM                      NA         NA
## y$PopulationFFD:y$GenotypePC200:y$SexM                     NA         NA
## y$PopulationPC:y$GenotypePC200:y$SexM               0.0071902  0.0192571
## y$PopulationFFD:y$GenotypePC85:y$SexM                      NA         NA
## y$PopulationPC:y$GenotypePC85:y$SexM                       NA         NA
## y$PopulationFFD:y$GenotypeFFD14:y$TempLow           0.0304943  0.0217177
## y$PopulationPC:y$GenotypeFFD14:y$TempLow                   NA         NA
## y$PopulationFFD:y$GenotypeFFD16:y$TempLow           0.0461627  0.0210799
## y$PopulationPC:y$GenotypeFFD16:y$TempLow                   NA         NA
## y$PopulationFFD:y$GenotypeFFD19:y$TempLow           0.0074276  0.0215171
## y$PopulationPC:y$GenotypeFFD19:y$TempLow                   NA         NA
## y$PopulationFFD:y$GenotypeFFD2:y$TempLow            0.0310611  0.0213924
## y$PopulationPC:y$GenotypeFFD2:y$TempLow                    NA         NA
## y$PopulationFFD:y$GenotypeFFD22:y$TempLow           0.0430397  0.0212179
```

```
## y$PopulationPC:y$GenotypeFFD22:y$TempLow                    NA         NA
## y$PopulationFFD:y$GenotypeFFD23:y$TempLow            0.0591971  0.0210726
## y$PopulationPC:y$GenotypeFFD23:y$TempLow                    NA         NA
## y$PopulationFFD:y$GenotypeFFD29:y$TempLow            0.0316690  0.0199082
## y$PopulationPC:y$GenotypeFFD29:y$TempLow                    NA         NA
## y$PopulationFFD:y$GenotypeFFD4:y$TempLow             0.0501705  0.0205606
## y$PopulationPC:y$GenotypeFFD4:y$TempLow                     NA         NA
## y$PopulationFFD:y$GenotypeFFD6:y$TempLow             0.0400287  0.0211712
## y$PopulationPC:y$GenotypeFFD6:y$TempLow                     NA         NA
## y$PopulationFFD:y$GenotypePC113:y$TempLow                   NA         NA
## y$PopulationPC:y$GenotypePC113:y$TempLow            -0.0442628  0.0208888
## y$PopulationFFD:y$GenotypePC114:y$TempLow                   NA         NA
## y$PopulationPC:y$GenotypePC114:y$TempLow             0.0235613  0.0204363
## y$PopulationFFD:y$GenotypePC12:y$TempLow                    NA         NA
## y$PopulationPC:y$GenotypePC12:y$TempLow              0.0362719  0.0197737
## y$PopulationFFD:y$GenotypePC136:y$TempLow                   NA         NA
## y$PopulationPC:y$GenotypePC136:y$TempLow             0.0151607  0.0206800
## y$PopulationFFD:y$GenotypePC141:y$TempLow                   NA         NA
## y$PopulationPC:y$GenotypePC141:y$TempLow            -0.0017678  0.0194266
## y$PopulationFFD:y$GenotypePC155:y$TempLow                   NA         NA
## y$PopulationPC:y$GenotypePC155:y$TempLow             0.0256037  0.0198370
## y$PopulationFFD:y$GenotypePC167:y$TempLow                   NA         NA
## y$PopulationPC:y$GenotypePC167:y$TempLow             0.0216483  0.0205175
## y$PopulationFFD:y$GenotypePC189:y$TempLow                   NA         NA
## y$PopulationPC:y$GenotypePC189:y$TempLow             0.0135271  0.0188151
## y$PopulationFFD:y$GenotypePC200:y$TempLow                   NA         NA
## y$PopulationPC:y$GenotypePC200:y$TempLow                    NA         NA
## y$PopulationFFD:y$GenotypePC85:y$TempLow                    NA         NA
## y$PopulationPC:y$GenotypePC85:y$TempLow                     NA         NA
## y$PopulationPC:y$SexM:y$TempLow                     -0.0057265  0.0289891
## y$PopulationFFD:y$GenotypeFFD14:y$FoodQ              0.0443078  0.0375828
## y$PopulationPC:y$GenotypeFFD14:y$FoodQ                      NA         NA
## y$PopulationFFD:y$GenotypeFFD16:y$FoodQ             -0.0094242  0.0213843
## y$PopulationPC:y$GenotypeFFD16:y$FoodQ                      NA         NA
## y$PopulationFFD:y$GenotypeFFD19:y$FoodQ             -0.0101637  0.0218495
## y$PopulationPC:y$GenotypeFFD19:y$FoodQ                      NA         NA
## y$PopulationFFD:y$GenotypeFFD2:y$FoodQ               0.0321560  0.0214895
## y$PopulationPC:y$GenotypeFFD2:y$FoodQ                       NA         NA
## y$PopulationFFD:y$GenotypeFFD22:y$FoodQ              0.0130151  0.0214895
## y$PopulationPC:y$GenotypeFFD22:y$FoodQ                      NA         NA
## y$PopulationFFD:y$GenotypeFFD23:y$FoodQ             -0.0663998  0.0207872
## y$PopulationPC:y$GenotypeFFD23:y$FoodQ                      NA         NA
## y$PopulationFFD:y$GenotypeFFD29:y$FoodQ             -0.0647705  0.0209653
## y$PopulationPC:y$GenotypeFFD29:y$FoodQ                      NA         NA
## y$PopulationFFD:y$GenotypeFFD4:y$FoodQ              -0.0312283  0.0203793
## y$PopulationPC:y$GenotypeFFD4:y$FoodQ                       NA         NA
## y$PopulationFFD:y$GenotypeFFD6:y$FoodQ              -0.0216443  0.0208871
## y$PopulationPC:y$GenotypeFFD6:y$FoodQ                       NA         NA
## y$PopulationFFD:y$GenotypePC113:y$FoodQ                     NA         NA
## y$PopulationPC:y$GenotypePC113:y$FoodQ               0.0892487  0.0195270
## y$PopulationFFD:y$GenotypePC114:y$FoodQ                     NA         NA
## y$PopulationPC:y$GenotypePC114:y$FoodQ               0.0452991  0.0203816
## y$PopulationFFD:y$GenotypePC12:y$FoodQ                      NA         NA
## y$PopulationPC:y$GenotypePC12:y$FoodQ                0.0044319  0.0202867
```

```
## y$PopulationFFD:y$GenotypePC136:y$FoodQ                          NA          NA
## y$PopulationPC:y$GenotypePC136:y$FoodQ                    0.0025804   0.0351323
## y$PopulationFFD:y$GenotypePC141:y$FoodQ                          NA          NA
## y$PopulationPC:y$GenotypePC141:y$FoodQ                    0.0152159   0.0195826
## y$PopulationFFD:y$GenotypePC155:y$FoodQ                          NA          NA
## y$PopulationPC:y$GenotypePC155:y$FoodQ                   -0.0199390   0.0195826
## y$PopulationFFD:y$GenotypePC167:y$FoodQ                          NA          NA
## y$PopulationPC:y$GenotypePC167:y$FoodQ                    0.0435143   0.0208206
## y$PopulationFFD:y$GenotypePC189:y$FoodQ                          NA          NA
## y$PopulationPC:y$GenotypePC189:y$FoodQ                   -0.0079093   0.0204835
## y$PopulationFFD:y$GenotypePC200:y$FoodQ                          NA          NA
## y$PopulationPC:y$GenotypePC200:y$FoodQ                   -0.0853193   0.0210089
## y$PopulationFFD:y$GenotypePC85:y$FoodQ                           NA          NA
## y$PopulationPC:y$GenotypePC85:y$FoodQ                            NA          NA
## y$PopulationPC:y$SexM:y$FoodQ                            -0.0089309   0.0290610
## y$PopulationPC:y$TempLow:y$FoodQ                         -0.0105936   0.0294165
## y$SexM:y$TempLow:y$FoodQ                                  0.0271703   0.0303026
## y$PopulationFFD:y$GenotypeFFD14:y$SexM:y$TempLow         -0.0090936   0.0305526
## y$PopulationPC:y$GenotypeFFD14:y$SexM:y$TempLow                  NA          NA
## y$PopulationFFD:y$GenotypeFFD16:y$SexM:y$TempLow         -0.0473017   0.0300666
## y$PopulationPC:y$GenotypeFFD16:y$SexM:y$TempLow                  NA          NA
## y$PopulationFFD:y$GenotypeFFD19:y$SexM:y$TempLow         -0.0060589   0.0300951
## y$PopulationPC:y$GenotypeFFD19:y$SexM:y$TempLow                  NA          NA
## y$PopulationFFD:y$GenotypeFFD2:y$SexM:y$TempLow           0.0005710   0.0303965
## y$PopulationPC:y$GenotypeFFD2:y$SexM:y$TempLow                   NA          NA
## y$PopulationFFD:y$GenotypeFFD22:y$SexM:y$TempLow         -0.0277728   0.0300290
## y$PopulationPC:y$GenotypeFFD22:y$SexM:y$TempLow                  NA          NA
## y$PopulationFFD:y$GenotypeFFD23:y$SexM:y$TempLow         -0.0527670   0.0290916
## y$PopulationPC:y$GenotypeFFD23:y$SexM:y$TempLow                  NA          NA
## y$PopulationFFD:y$GenotypeFFD29:y$SexM:y$TempLow         -0.0517825   0.0293031
## y$PopulationPC:y$GenotypeFFD29:y$SexM:y$TempLow                  NA          NA
## y$PopulationFFD:y$GenotypeFFD4:y$SexM:y$TempLow          -0.0183075   0.0292186
## y$PopulationPC:y$GenotypeFFD4:y$SexM:y$TempLow                   NA          NA
## y$PopulationFFD:y$GenotypeFFD6:y$SexM:y$TempLow          -0.0285773   0.0295223
## y$PopulationPC:y$GenotypeFFD6:y$SexM:y$TempLow                   NA          NA
## y$PopulationFFD:y$GenotypePC113:y$SexM:y$TempLow                 NA          NA
## y$PopulationPC:y$GenotypePC113:y$SexM:y$TempLow          -0.0454502   0.0294882
## y$PopulationFFD:y$GenotypePC114:y$SexM:y$TempLow                 NA          NA
## y$PopulationPC:y$GenotypePC114:y$SexM:y$TempLow           0.0041772   0.0281372
## y$PopulationFFD:y$GenotypePC12:y$SexM:y$TempLow                  NA          NA
## y$PopulationPC:y$GenotypePC12:y$SexM:y$TempLow            0.0127850   0.0285703
## y$PopulationFFD:y$GenotypePC136:y$SexM:y$TempLow                 NA          NA
## y$PopulationPC:y$GenotypePC136:y$SexM:y$TempLow          -0.0193284   0.0287774
## y$PopulationFFD:y$GenotypePC141:y$SexM:y$TempLow                 NA          NA
## y$PopulationPC:y$GenotypePC141:y$SexM:y$TempLow          -0.0008772   0.0273595
## y$PopulationFFD:y$GenotypePC155:y$SexM:y$TempLow                 NA          NA
## y$PopulationPC:y$GenotypePC155:y$SexM:y$TempLow          -0.0058687   0.0277611
## y$PopulationFFD:y$GenotypePC167:y$SexM:y$TempLow                 NA          NA
## y$PopulationPC:y$GenotypePC167:y$SexM:y$TempLow                  NA          NA
## y$PopulationFFD:y$GenotypePC189:y$SexM:y$TempLow                 NA          NA
## y$PopulationPC:y$GenotypePC189:y$SexM:y$TempLow                  NA          NA
## y$PopulationFFD:y$GenotypePC200:y$SexM:y$TempLow                 NA          NA
## y$PopulationPC:y$GenotypePC200:y$SexM:y$TempLow                  NA          NA
## y$PopulationFFD:y$GenotypePC85:y$SexM:y$TempLow                  NA          NA
```

```
## y$PopulationPC:y$GenotypePC85:y$SexM:y$TempLow                 NA          NA
## y$PopulationFFD:y$GenotypeFFD14:y$SexM:y$FoodQ          0.0052564   0.0307533
## y$PopulationPC:y$GenotypeFFD14:y$SexM:y$FoodQ                  NA          NA
## y$PopulationFFD:y$GenotypeFFD16:y$SexM:y$FoodQ         -0.0202579   0.0310490
## y$PopulationPC:y$GenotypeFFD16:y$SexM:y$FoodQ                  NA          NA
## y$PopulationFFD:y$GenotypeFFD19:y$SexM:y$FoodQ          0.0257025   0.0295612
## y$PopulationPC:y$GenotypeFFD19:y$SexM:y$FoodQ                  NA          NA
## y$PopulationFFD:y$GenotypeFFD2:y$SexM:y$FoodQ          -0.0001082   0.0301526
## y$PopulationPC:y$GenotypeFFD2:y$SexM:y$FoodQ                   NA          NA
## y$PopulationFFD:y$GenotypeFFD22:y$SexM:y$FoodQ         -0.0017246   0.0301700
## y$PopulationPC:y$GenotypeFFD22:y$SexM:y$FoodQ                  NA          NA
## y$PopulationFFD:y$GenotypeFFD23:y$SexM:y$FoodQ          0.0175726   0.0296628
## y$PopulationPC:y$GenotypeFFD23:y$SexM:y$FoodQ                  NA          NA
## y$PopulationFFD:y$GenotypeFFD29:y$SexM:y$FoodQ          0.0296823   0.0301320
## y$PopulationPC:y$GenotypeFFD29:y$SexM:y$FoodQ                  NA          NA
## y$PopulationFFD:y$GenotypeFFD4:y$SexM:y$FoodQ           0.0165245   0.0288614
## y$PopulationPC:y$GenotypeFFD4:y$SexM:y$FoodQ                   NA          NA
## y$PopulationFFD:y$GenotypeFFD6:y$SexM:y$FoodQ          -0.0046393   0.0290442
## y$PopulationPC:y$GenotypeFFD6:y$SexM:y$FoodQ                   NA          NA
## y$PopulationFFD:y$GenotypePC113:y$SexM:y$FoodQ                 NA          NA
## y$PopulationPC:y$GenotypePC113:y$SexM:y$FoodQ          -0.1582227   0.0281703
## y$PopulationFFD:y$GenotypePC114:y$SexM:y$FoodQ                 NA          NA
## y$PopulationPC:y$GenotypePC114:y$SexM:y$FoodQ          -0.0010783   0.0284493
## y$PopulationFFD:y$GenotypePC12:y$SexM:y$FoodQ                  NA          NA
## y$PopulationPC:y$GenotypePC12:y$SexM:y$FoodQ            0.0407836   0.0287761
## y$PopulationFFD:y$GenotypePC136:y$SexM:y$FoodQ                 NA          NA
## y$PopulationPC:y$GenotypePC136:y$SexM:y$FoodQ          -0.0117374   0.0286989
## y$PopulationFFD:y$GenotypePC141:y$SexM:y$FoodQ                 NA          NA
## y$PopulationPC:y$GenotypePC141:y$SexM:y$FoodQ           0.0022304   0.0273108
## y$PopulationFFD:y$GenotypePC155:y$SexM:y$FoodQ                 NA          NA
## y$PopulationPC:y$GenotypePC155:y$SexM:y$FoodQ          -0.0279938   0.0274208
## y$PopulationFFD:y$GenotypePC167:y$SexM:y$FoodQ                 NA          NA
## y$PopulationPC:y$GenotypePC167:y$SexM:y$FoodQ           0.0045263   0.0299509
## y$PopulationFFD:y$GenotypePC189:y$SexM:y$FoodQ                 NA          NA
## y$PopulationPC:y$GenotypePC189:y$SexM:y$FoodQ                  NA          NA
## y$PopulationFFD:y$GenotypePC200:y$SexM:y$FoodQ                 NA          NA
## y$PopulationPC:y$GenotypePC200:y$SexM:y$FoodQ                  NA          NA
## y$PopulationFFD:y$GenotypePC85:y$SexM:y$FoodQ                  NA          NA
## y$PopulationPC:y$GenotypePC85:y$SexM:y$FoodQ                   NA          NA
## y$PopulationFFD:y$GenotypeFFD14:y$TempLow:y$FoodQ      -0.0864991   0.0314845
## y$PopulationPC:y$GenotypeFFD14:y$TempLow:y$FoodQ               NA          NA
## y$PopulationFFD:y$GenotypeFFD16:y$TempLow:y$FoodQ      -0.0187945   0.0298686
## y$PopulationPC:y$GenotypeFFD16:y$TempLow:y$FoodQ               NA          NA
## y$PopulationFFD:y$GenotypeFFD19:y$TempLow:y$FoodQ      -0.0130678   0.0302035
## y$PopulationPC:y$GenotypeFFD19:y$TempLow:y$FoodQ               NA          NA
## y$PopulationFFD:y$GenotypeFFD2:y$TempLow:y$FoodQ       -0.1621149   0.0301526
## y$PopulationPC:y$GenotypeFFD2:y$TempLow:y$FoodQ                NA          NA
## y$PopulationFFD:y$GenotypeFFD22:y$TempLow:y$FoodQ      -0.0904703   0.0298842
## y$PopulationPC:y$GenotypeFFD22:y$TempLow:y$FoodQ               NA          NA
## y$PopulationFFD:y$GenotypeFFD23:y$TempLow:y$FoodQ       0.0453689   0.0294441
## y$PopulationPC:y$GenotypeFFD23:y$TempLow:y$FoodQ               NA          NA
## y$PopulationFFD:y$GenotypeFFD29:y$TempLow:y$FoodQ       0.0738373   0.0285771
## y$PopulationPC:y$GenotypeFFD29:y$TempLow:y$FoodQ               NA          NA
## y$PopulationFFD:y$GenotypeFFD4:y$TempLow:y$FoodQ               NA          NA
```

```
## y$PopulationPC:y$GenotypeFFD4:y$TempLow:y$FoodQ                      NA         NA
## y$PopulationFFD:y$GenotypeFFD6:y$TempLow:y$FoodQ              0.0318274  0.0297263
## y$PopulationPC:y$GenotypeFFD6:y$TempLow:y$FoodQ                      NA         NA
## y$PopulationFFD:y$GenotypePC113:y$TempLow:y$FoodQ                    NA         NA
## y$PopulationPC:y$GenotypePC113:y$TempLow:y$FoodQ                     NA         NA
## y$PopulationFFD:y$GenotypePC114:y$TempLow:y$FoodQ                    NA         NA
## y$PopulationPC:y$GenotypePC114:y$TempLow:y$FoodQ             -0.0032269  0.0289693
## y$PopulationFFD:y$GenotypePC12:y$TempLow:y$FoodQ                     NA         NA
## y$PopulationPC:y$GenotypePC12:y$TempLow:y$FoodQ               0.0268078  0.0288099
## y$PopulationFFD:y$GenotypePC136:y$TempLow:y$FoodQ                    NA         NA
## y$PopulationPC:y$GenotypePC136:y$TempLow:y$FoodQ             -0.0031325  0.0283275
## y$PopulationFFD:y$GenotypePC141:y$TempLow:y$FoodQ                    NA         NA
## y$PopulationPC:y$GenotypePC141:y$TempLow:y$FoodQ             -0.0022938  0.0282553
## y$PopulationFFD:y$GenotypePC155:y$TempLow:y$FoodQ                    NA         NA
## y$PopulationPC:y$GenotypePC155:y$TempLow:y$FoodQ              0.0631619  0.0282318
## y$PopulationFFD:y$GenotypePC167:y$TempLow:y$FoodQ                    NA         NA
## y$PopulationPC:y$GenotypePC167:y$TempLow:y$FoodQ              0.0368786  0.0297834
## y$PopulationFFD:y$GenotypePC189:y$TempLow:y$FoodQ                    NA         NA
## y$PopulationPC:y$GenotypePC189:y$TempLow:y$FoodQ              0.0182403  0.0281606
## y$PopulationFFD:y$GenotypePC200:y$TempLow:y$FoodQ                    NA         NA
## y$PopulationPC:y$GenotypePC200:y$TempLow:y$FoodQ                     NA         NA
## y$PopulationFFD:y$GenotypePC85:y$TempLow:y$FoodQ                     NA         NA
## y$PopulationPC:y$GenotypePC85:y$TempLow:y$FoodQ                      NA         NA
## y$PopulationPC:y$SexM:y$TempLow:y$FoodQ                      -0.0086304  0.0409899
## y$PopulationFFD:y$GenotypeFFD14:y$SexM:y$TempLow:y$FoodQ             NA         NA
## y$PopulationPC:y$GenotypeFFD14:y$SexM:y$TempLow:y$FoodQ              NA         NA
## y$PopulationFFD:y$GenotypeFFD16:y$SexM:y$TempLow:y$FoodQ      0.0369284  0.0430762
## y$PopulationPC:y$GenotypeFFD16:y$SexM:y$TempLow:y$FoodQ              NA         NA
## y$PopulationFFD:y$GenotypeFFD19:y$SexM:y$TempLow:y$FoodQ             NA         NA
## y$PopulationPC:y$GenotypeFFD19:y$SexM:y$TempLow:y$FoodQ              NA         NA
## y$PopulationFFD:y$GenotypeFFD2:y$SexM:y$TempLow:y$FoodQ              NA         NA
## y$PopulationPC:y$GenotypeFFD2:y$SexM:y$TempLow:y$FoodQ               NA         NA
## y$PopulationFFD:y$GenotypeFFD22:y$SexM:y$TempLow:y$FoodQ -0.0388556  0.0422330
## y$PopulationPC:y$GenotypeFFD22:y$SexM:y$TempLow:y$FoodQ              NA         NA
## y$PopulationFFD:y$GenotypeFFD23:y$SexM:y$TempLow:y$FoodQ             NA         NA
## y$PopulationPC:y$GenotypeFFD23:y$SexM:y$TempLow:y$FoodQ              NA         NA
## y$PopulationFFD:y$GenotypeFFD29:y$SexM:y$TempLow:y$FoodQ             NA         NA
## y$PopulationPC:y$GenotypeFFD29:y$SexM:y$TempLow:y$FoodQ              NA         NA
## y$PopulationFFD:y$GenotypeFFD4:y$SexM:y$TempLow:y$FoodQ              NA         NA
## y$PopulationPC:y$GenotypeFFD4:y$SexM:y$TempLow:y$FoodQ               NA         NA
## y$PopulationFFD:y$GenotypeFFD6:y$SexM:y$TempLow:y$FoodQ       0.0148038  0.0427611
## y$PopulationPC:y$GenotypeFFD6:y$SexM:y$TempLow:y$FoodQ               NA         NA
## y$PopulationFFD:y$GenotypePC113:y$SexM:y$TempLow:y$FoodQ             NA         NA
## y$PopulationPC:y$GenotypePC113:y$SexM:y$TempLow:y$FoodQ              NA         NA
## y$PopulationFFD:y$GenotypePC114:y$SexM:y$TempLow:y$FoodQ             NA         NA
## y$PopulationPC:y$GenotypePC114:y$SexM:y$TempLow:y$FoodQ      -0.0476815  0.0406491
## y$PopulationFFD:y$GenotypePC12:y$SexM:y$TempLow:y$FoodQ              NA         NA
## y$PopulationPC:y$GenotypePC12:y$SexM:y$TempLow:y$FoodQ       -0.0384399  0.0406345
## y$PopulationFFD:y$GenotypePC136:y$SexM:y$TempLow:y$FoodQ             NA         NA
## y$PopulationPC:y$GenotypePC136:y$SexM:y$TempLow:y$FoodQ              NA         NA
## y$PopulationFFD:y$GenotypePC141:y$SexM:y$TempLow:y$FoodQ             NA         NA
## y$PopulationPC:y$GenotypePC141:y$SexM:y$TempLow:y$FoodQ      -0.0327217  0.0392589
## y$PopulationFFD:y$GenotypePC155:y$SexM:y$TempLow:y$FoodQ             NA         NA
## y$PopulationPC:y$GenotypePC155:y$SexM:y$TempLow:y$FoodQ       0.0001884  0.0395851
```

```
## y$PopulationFFD:y$GenotypePC167:y$SexM:y$TempLow:y$FoodQ         NA         NA
## y$PopulationPC:y$GenotypePC167:y$SexM:y$TempLow:y$FoodQ          NA         NA
## y$PopulationFFD:y$GenotypePC189:y$SexM:y$TempLow:y$FoodQ         NA         NA
## y$PopulationPC:y$GenotypePC189:y$SexM:y$TempLow:y$FoodQ          NA         NA
## y$PopulationFFD:y$GenotypePC200:y$SexM:y$TempLow:y$FoodQ         NA         NA
## y$PopulationPC:y$GenotypePC200:y$SexM:y$TempLow:y$FoodQ          NA         NA
## y$PopulationFFD:y$GenotypePC85:y$SexM:y$TempLow:y$FoodQ          NA         NA
## y$PopulationPC:y$GenotypePC85:y$SexM:y$TempLow:y$FoodQ           NA         NA
##                                                           t value Pr(>|t|)
## (Intercept)                                               -48.525  < 2e-16 ***
## y$PopulationPC                                             -1.362  0.17340
## y$SexM                                                     -2.812  0.00496 **
## y$TempLow                                                   0.933  0.35104
## y$FoodQ                                                    -5.447 5.48e-08 ***
## y$PopulationFFD:y$GenotypeFFD14                             0.109  0.91351
## y$PopulationPC:y$GenotypeFFD14                                 NA       NA
## y$PopulationFFD:y$GenotypeFFD16                            -1.162  0.24522
## y$PopulationPC:y$GenotypeFFD16                                 NA       NA
## y$PopulationFFD:y$GenotypeFFD19                             0.967  0.33384
## y$PopulationPC:y$GenotypeFFD19                                 NA       NA
## y$PopulationFFD:y$GenotypeFFD2                              0.313  0.75392
## y$PopulationPC:y$GenotypeFFD2                                  NA       NA
## y$PopulationFFD:y$GenotypeFFD22                            -1.765  0.07766 .
## y$PopulationPC:y$GenotypeFFD22                                 NA       NA
## y$PopulationFFD:y$GenotypeFFD23                             0.513  0.60791
## y$PopulationPC:y$GenotypeFFD23                                 NA       NA
## y$PopulationFFD:y$GenotypeFFD29                             0.221  0.82488
## y$PopulationPC:y$GenotypeFFD29                                 NA       NA
## y$PopulationFFD:y$GenotypeFFD4                              0.322  0.74780
## y$PopulationPC:y$GenotypeFFD4                                  NA       NA
## y$PopulationFFD:y$GenotypeFFD6                             -1.375  0.16909
## y$PopulationPC:y$GenotypeFFD6                                  NA       NA
## y$PopulationFFD:y$GenotypePC113                                NA       NA
## y$PopulationPC:y$GenotypePC113                             -5.637 1.87e-08 ***
## y$PopulationFFD:y$GenotypePC114                                NA       NA
## y$PopulationPC:y$GenotypePC114                              0.072  0.94297
## y$PopulationFFD:y$GenotypePC12                                 NA       NA
## y$PopulationPC:y$GenotypePC12                              -0.850  0.39562
## y$PopulationFFD:y$GenotypePC136                                NA       NA
## y$PopulationPC:y$GenotypePC136                             -1.454  0.14595
## y$PopulationFFD:y$GenotypePC141                                NA       NA
## y$PopulationPC:y$GenotypePC141                              1.464  0.14337
## y$PopulationFFD:y$GenotypePC155                                NA       NA
## y$PopulationPC:y$GenotypePC155                              1.080  0.28007
## y$PopulationFFD:y$GenotypePC167                                NA       NA
## y$PopulationPC:y$GenotypePC167                              1.351  0.17684
## y$PopulationFFD:y$GenotypePC189                                NA       NA
## y$PopulationPC:y$GenotypePC189                              1.016  0.30990
## y$PopulationFFD:y$GenotypePC200                                NA       NA
## y$PopulationPC:y$GenotypePC200                              2.945  0.00325 **
## y$PopulationFFD:y$GenotypePC85                                 NA       NA
## y$PopulationPC:y$GenotypePC85                                  NA       NA
## y$PopulationPC:y$SexM                                       0.492  0.62286
## y$PopulationPC:y$TempLow                                    0.271  0.78618
```

```
## y$SexM:y$TempLow                                       -0.147  0.88334
## y$PopulationPC:y$FoodQ                                 -0.458  0.64671
## y$SexM:y$FoodQ                                          0.503  0.61525
## y$TempLow:y$FoodQ                                       0.119  0.90528
## y$PopulationFFD:y$GenotypeFFD14:y$SexM                  0.068  0.94549
## y$PopulationPC:y$GenotypeFFD14:y$SexM                      NA       NA
## y$PopulationFFD:y$GenotypeFFD16:y$SexM                  0.972  0.33110
## y$PopulationPC:y$GenotypeFFD16:y$SexM                      NA       NA
## y$PopulationFFD:y$GenotypeFFD19:y$SexM                 -0.339  0.73457
## y$PopulationPC:y$GenotypeFFD19:y$SexM                      NA       NA
## y$PopulationFFD:y$GenotypeFFD2:y$SexM                  -0.690  0.49013
## y$PopulationPC:y$GenotypeFFD2:y$SexM                       NA       NA
## y$PopulationFFD:y$GenotypeFFD22:y$SexM                  1.990  0.04662 *
## y$PopulationPC:y$GenotypeFFD22:y$SexM                      NA       NA
## y$PopulationFFD:y$GenotypeFFD23:y$SexM                 -0.089  0.92927
## y$PopulationPC:y$GenotypeFFD23:y$SexM                      NA       NA
## y$PopulationFFD:y$GenotypeFFD29:y$SexM                 -0.424  0.67138
## y$PopulationPC:y$GenotypeFFD29:y$SexM                      NA       NA
## y$PopulationFFD:y$GenotypeFFD4:y$SexM                   0.924  0.35571
## y$PopulationPC:y$GenotypeFFD4:y$SexM                       NA       NA
## y$PopulationFFD:y$GenotypeFFD6:y$SexM                  -0.064  0.94916
## y$PopulationPC:y$GenotypeFFD6:y$SexM                       NA       NA
## y$PopulationFFD:y$GenotypePC113:y$SexM                     NA       NA
## y$PopulationPC:y$GenotypePC113:y$SexM                   4.823 1.48e-06 ***
## y$PopulationFFD:y$GenotypePC114:y$SexM                     NA       NA
## y$PopulationPC:y$GenotypePC114:y$SexM                  -0.791  0.42875
## y$PopulationFFD:y$GenotypePC12:y$SexM                      NA       NA
## y$PopulationPC:y$GenotypePC12:y$SexM                   -0.921  0.35711
## y$PopulationFFD:y$GenotypePC136:y$SexM                     NA       NA
## y$PopulationPC:y$GenotypePC136:y$SexM                   0.404  0.68593
## y$PopulationFFD:y$GenotypePC141:y$SexM                     NA       NA
## y$PopulationPC:y$GenotypePC141:y$SexM                  -0.658  0.51066
## y$PopulationFFD:y$GenotypePC155:y$SexM                     NA       NA
## y$PopulationPC:y$GenotypePC155:y$SexM                   0.396  0.69235
## y$PopulationFFD:y$GenotypePC167:y$SexM                     NA       NA
## y$PopulationPC:y$GenotypePC167:y$SexM                  -1.520  0.12859
## y$PopulationFFD:y$GenotypePC189:y$SexM                     NA       NA
## y$PopulationPC:y$GenotypePC189:y$SexM                      NA       NA
## y$PopulationFFD:y$GenotypePC200:y$SexM                     NA       NA
## y$PopulationPC:y$GenotypePC200:y$SexM                   0.373  0.70889
## y$PopulationFFD:y$GenotypePC85:y$SexM                      NA       NA
## y$PopulationPC:y$GenotypePC85:y$SexM                       NA       NA
## y$PopulationFFD:y$GenotypeFFD14:y$TempLow               1.404  0.16037
## y$PopulationPC:y$GenotypeFFD14:y$TempLow                   NA       NA
## y$PopulationFFD:y$GenotypeFFD16:y$TempLow               2.190  0.02860 *
## y$PopulationPC:y$GenotypeFFD16:y$TempLow                   NA       NA
## y$PopulationFFD:y$GenotypeFFD19:y$TempLow               0.345  0.72997
## y$PopulationPC:y$GenotypeFFD19:y$TempLow                   NA       NA
## y$PopulationFFD:y$GenotypeFFD2:y$TempLow                1.452  0.14660
## y$PopulationPC:y$GenotypeFFD2:y$TempLow                    NA       NA
## y$PopulationFFD:y$GenotypeFFD22:y$TempLow               2.028  0.04259 *
## y$PopulationPC:y$GenotypeFFD22:y$TempLow                   NA       NA
## y$PopulationFFD:y$GenotypeFFD23:y$TempLow               2.809  0.00499 **
## y$PopulationPC:y$GenotypeFFD23:y$TempLow                   NA       NA
```

```
## y$PopulationFFD:y$GenotypeFFD29:y$TempLow                   1.591  0.11176
## y$PopulationPC:y$GenotypeFFD29:y$TempLow                       NA       NA
## y$PopulationFFD:y$GenotypeFFD4:y$TempLow                    2.440  0.01473 *
## y$PopulationPC:y$GenotypeFFD4:y$TempLow                        NA       NA
## y$PopulationFFD:y$GenotypeFFD6:y$TempLow                    1.891  0.05875 .
## y$PopulationPC:y$GenotypeFFD6:y$TempLow                        NA       NA
## y$PopulationFFD:y$GenotypePC113:y$TempLow                      NA       NA
## y$PopulationPC:y$GenotypePC113:y$TempLow                   -2.119  0.03416 *
## y$PopulationFFD:y$GenotypePC114:y$TempLow                      NA       NA
## y$PopulationPC:y$GenotypePC114:y$TempLow                    1.153  0.24903
## y$PopulationFFD:y$GenotypePC12:y$TempLow                       NA       NA
## y$PopulationPC:y$GenotypePC12:y$TempLow                     1.834  0.06669 .
## y$PopulationFFD:y$GenotypePC136:y$TempLow                      NA       NA
## y$PopulationPC:y$GenotypePC136:y$TempLow                    0.733  0.46354
## y$PopulationFFD:y$GenotypePC141:y$TempLow                      NA       NA
## y$PopulationPC:y$GenotypePC141:y$TempLow                   -0.091  0.92750
## y$PopulationFFD:y$GenotypePC155:y$TempLow                      NA       NA
## y$PopulationPC:y$GenotypePC155:y$TempLow                    1.291  0.19689
## y$PopulationFFD:y$GenotypePC167:y$TempLow                      NA       NA
## y$PopulationPC:y$GenotypePC167:y$TempLow                    1.055  0.29145
## y$PopulationFFD:y$GenotypePC189:y$TempLow                      NA       NA
## y$PopulationPC:y$GenotypePC189:y$TempLow                    0.719  0.47222
## y$PopulationFFD:y$GenotypePC200:y$TempLow                      NA       NA
## y$PopulationPC:y$GenotypePC200:y$TempLow                       NA       NA
## y$PopulationFFD:y$GenotypePC85:y$TempLow                       NA       NA
## y$PopulationPC:y$GenotypePC85:y$TempLow                        NA       NA
## y$PopulationPC:y$SexM:y$TempLow                            -0.198  0.84342
## y$PopulationFFD:y$GenotypeFFD14:y$FoodQ                     1.179  0.23850
## y$PopulationPC:y$GenotypeFFD14:y$FoodQ                         NA       NA
## y$PopulationFFD:y$GenotypeFFD16:y$FoodQ                    -0.441  0.65945
## y$PopulationPC:y$GenotypeFFD16:y$FoodQ                         NA       NA
## y$PopulationFFD:y$GenotypeFFD19:y$FoodQ                    -0.465  0.64184
## y$PopulationPC:y$GenotypeFFD19:y$FoodQ                         NA       NA
## y$PopulationFFD:y$GenotypeFFD2:y$FoodQ                      1.496  0.13465
## y$PopulationPC:y$GenotypeFFD2:y$FoodQ                          NA       NA
## y$PopulationFFD:y$GenotypeFFD22:y$FoodQ                     0.606  0.54479
## y$PopulationPC:y$GenotypeFFD22:y$FoodQ                         NA       NA
## y$PopulationFFD:y$GenotypeFFD23:y$FoodQ                    -3.194  0.00141 **
## y$PopulationPC:y$GenotypeFFD23:y$FoodQ                         NA       NA
## y$PopulationFFD:y$GenotypeFFD29:y$FoodQ                    -3.089  0.00202 **
## y$PopulationPC:y$GenotypeFFD29:y$FoodQ                         NA       NA
## y$PopulationFFD:y$GenotypeFFD4:y$FoodQ                     -1.532  0.12553
## y$PopulationPC:y$GenotypeFFD4:y$FoodQ                          NA       NA
## y$PopulationFFD:y$GenotypeFFD6:y$FoodQ                     -1.036  0.30016
## y$PopulationPC:y$GenotypeFFD6:y$FoodQ                          NA       NA
## y$PopulationFFD:y$GenotypePC113:y$FoodQ                        NA       NA
## y$PopulationPC:y$GenotypePC113:y$FoodQ                      4.571 5.03e-06 ***
## y$PopulationFFD:y$GenotypePC114:y$FoodQ                        NA       NA
## y$PopulationPC:y$GenotypePC114:y$FoodQ                      2.223  0.02631 *
## y$PopulationFFD:y$GenotypePC12:y$FoodQ                         NA       NA
## y$PopulationPC:y$GenotypePC12:y$FoodQ                       0.218  0.82708
## y$PopulationFFD:y$GenotypePC136:y$FoodQ                        NA       NA
## y$PopulationPC:y$GenotypePC136:y$FoodQ                      0.073  0.94145
## y$PopulationFFD:y$GenotypePC141:y$FoodQ                        NA       NA
```

```
## y$PopulationPC:y$GenotypePC141:y$FoodQ                     0.777  0.43721    
## y$PopulationFFD:y$GenotypePC155:y$FoodQ                       NA       NA    
## y$PopulationPC:y$GenotypePC155:y$FoodQ                    -1.018  0.30866    
## y$PopulationFFD:y$GenotypePC167:y$FoodQ                       NA       NA    
## y$PopulationPC:y$GenotypePC167:y$FoodQ                     2.090  0.03669 *  
## y$PopulationFFD:y$GenotypePC189:y$FoodQ                       NA       NA    
## y$PopulationPC:y$GenotypePC189:y$FoodQ                    -0.386  0.69942    
## y$PopulationFFD:y$GenotypePC200:y$FoodQ                       NA       NA    
## y$PopulationPC:y$GenotypePC200:y$FoodQ                    -4.061 4.99e-05 ***
## y$PopulationFFD:y$GenotypePC85:y$FoodQ                        NA       NA    
## y$PopulationPC:y$GenotypePC85:y$FoodQ                         NA       NA    
## y$PopulationPC:y$SexM:y$FoodQ                             -0.307  0.75862    
## y$PopulationPC:y$TempLow:y$FoodQ                          -0.360  0.71878    
## y$SexM:y$TempLow:y$FoodQ                                   0.897  0.36998    
## y$PopulationFFD:y$GenotypeFFD14:y$SexM:y$TempLow          -0.298  0.76600    
## y$PopulationPC:y$GenotypeFFD14:y$SexM:y$TempLow               NA       NA    
## y$PopulationFFD:y$GenotypeFFD16:y$SexM:y$TempLow          -1.573  0.11576    
## y$PopulationPC:y$GenotypeFFD16:y$SexM:y$TempLow               NA       NA    
## y$PopulationFFD:y$GenotypeFFD19:y$SexM:y$TempLow          -0.201  0.84046    
## y$PopulationPC:y$GenotypeFFD19:y$SexM:y$TempLow               NA       NA    
## y$PopulationFFD:y$GenotypeFFD2:y$SexM:y$TempLow            0.019  0.98501    
## y$PopulationPC:y$GenotypeFFD2:y$SexM:y$TempLow                NA       NA    
## y$PopulationFFD:y$GenotypeFFD22:y$SexM:y$TempLow          -0.925  0.35510    
## y$PopulationPC:y$GenotypeFFD22:y$SexM:y$TempLow               NA       NA    
## y$PopulationFFD:y$GenotypeFFD23:y$SexM:y$TempLow          -1.814  0.06979 .  
## y$PopulationPC:y$GenotypeFFD23:y$SexM:y$TempLow               NA       NA    
## y$PopulationFFD:y$GenotypeFFD29:y$SexM:y$TempLow          -1.767  0.07729 .  
## y$PopulationPC:y$GenotypeFFD29:y$SexM:y$TempLow               NA       NA    
## y$PopulationFFD:y$GenotypeFFD4:y$SexM:y$TempLow           -0.627  0.53098    
## y$PopulationPC:y$GenotypeFFD4:y$SexM:y$TempLow                NA       NA    
## y$PopulationFFD:y$GenotypeFFD6:y$SexM:y$TempLow           -0.968  0.33312    
## y$PopulationPC:y$GenotypeFFD6:y$SexM:y$TempLow                NA       NA    
## y$PopulationFFD:y$GenotypePC113:y$SexM:y$TempLow              NA       NA    
## y$PopulationPC:y$GenotypePC113:y$SexM:y$TempLow           -1.541  0.12333    
## y$PopulationFFD:y$GenotypePC114:y$SexM:y$TempLow              NA       NA    
## y$PopulationPC:y$GenotypePC114:y$SexM:y$TempLow            0.148  0.88199    
## y$PopulationFFD:y$GenotypePC12:y$SexM:y$TempLow               NA       NA    
## y$PopulationPC:y$GenotypePC12:y$SexM:y$TempLow             0.447  0.65455    
## y$PopulationFFD:y$GenotypePC136:y$SexM:y$TempLow              NA       NA    
## y$PopulationPC:y$GenotypePC136:y$SexM:y$TempLow           -0.672  0.50185    
## y$PopulationFFD:y$GenotypePC141:y$SexM:y$TempLow              NA       NA    
## y$PopulationPC:y$GenotypePC141:y$SexM:y$TempLow           -0.032  0.97442    
## y$PopulationFFD:y$GenotypePC155:y$SexM:y$TempLow              NA       NA    
## y$PopulationPC:y$GenotypePC155:y$SexM:y$TempLow           -0.211  0.83259    
## y$PopulationFFD:y$GenotypePC167:y$SexM:y$TempLow              NA       NA    
## y$PopulationPC:y$GenotypePC167:y$SexM:y$TempLow               NA       NA    
## y$PopulationFFD:y$GenotypePC189:y$SexM:y$TempLow              NA       NA    
## y$PopulationPC:y$GenotypePC189:y$SexM:y$TempLow               NA       NA    
## y$PopulationFFD:y$GenotypePC200:y$SexM:y$TempLow              NA       NA    
## y$PopulationPC:y$GenotypePC200:y$SexM:y$TempLow               NA       NA    
## y$PopulationFFD:y$GenotypePC85:y$SexM:y$TempLow               NA       NA    
## y$PopulationPC:y$GenotypePC85:y$SexM:y$TempLow                NA       NA    
## y$PopulationFFD:y$GenotypeFFD14:y$SexM:y$FoodQ             0.171  0.86429    
## y$PopulationPC:y$GenotypeFFD14:y$SexM:y$FoodQ                 NA       NA    
```

```
## y$PopulationFFD:y$GenotypeFFD16:y$SexM:y$FoodQ           -0.652  0.51416
## y$PopulationPC:y$GenotypeFFD16:y$SexM:y$FoodQ                NA       NA
## y$PopulationFFD:y$GenotypeFFD19:y$SexM:y$FoodQ            0.869  0.38465
## y$PopulationPC:y$GenotypeFFD19:y$SexM:y$FoodQ                NA       NA
## y$PopulationFFD:y$GenotypeFFD2:y$SexM:y$FoodQ            -0.004  0.99714
## y$PopulationPC:y$GenotypeFFD2:y$SexM:y$FoodQ                 NA       NA
## y$PopulationFFD:y$GenotypeFFD22:y$SexM:y$FoodQ           -0.057  0.95442
## y$PopulationPC:y$GenotypeFFD22:y$SexM:y$FoodQ                NA       NA
## y$PopulationFFD:y$GenotypeFFD23:y$SexM:y$FoodQ            0.592  0.55361
## y$PopulationPC:y$GenotypeFFD23:y$SexM:y$FoodQ                NA       NA
## y$PopulationFFD:y$GenotypeFFD29:y$SexM:y$FoodQ            0.985  0.32466
## y$PopulationPC:y$GenotypeFFD29:y$SexM:y$FoodQ                NA       NA
## y$PopulationFFD:y$GenotypeFFD4:y$SexM:y$FoodQ             0.573  0.56699
## y$PopulationPC:y$GenotypeFFD4:y$SexM:y$FoodQ                 NA       NA
## y$PopulationFFD:y$GenotypeFFD6:y$SexM:y$FoodQ            -0.160  0.87310
## y$PopulationPC:y$GenotypeFFD6:y$SexM:y$FoodQ                 NA       NA
## y$PopulationFFD:y$GenotypePC113:y$SexM:y$FoodQ               NA       NA
## y$PopulationPC:y$GenotypePC113:y$SexM:y$FoodQ            -5.617 2.10e-08 ***
## y$PopulationFFD:y$GenotypePC114:y$SexM:y$FoodQ               NA       NA
## y$PopulationPC:y$GenotypePC114:y$SexM:y$FoodQ            -0.038  0.96977
## y$PopulationFFD:y$GenotypePC12:y$SexM:y$FoodQ                NA       NA
## y$PopulationPC:y$GenotypePC12:y$SexM:y$FoodQ              1.417  0.15649
## y$PopulationFFD:y$GenotypePC136:y$SexM:y$FoodQ               NA       NA
## y$PopulationPC:y$GenotypePC136:y$SexM:y$FoodQ            -0.409  0.68258
## y$PopulationFFD:y$GenotypePC141:y$SexM:y$FoodQ               NA       NA
## y$PopulationPC:y$GenotypePC141:y$SexM:y$FoodQ             0.082  0.93492
## y$PopulationFFD:y$GenotypePC155:y$SexM:y$FoodQ               NA       NA
## y$PopulationPC:y$GenotypePC155:y$SexM:y$FoodQ            -1.021  0.30737
## y$PopulationFFD:y$GenotypePC167:y$SexM:y$FoodQ               NA       NA
## y$PopulationPC:y$GenotypePC167:y$SexM:y$FoodQ             0.151  0.87989
## y$PopulationFFD:y$GenotypePC189:y$SexM:y$FoodQ               NA       NA
## y$PopulationPC:y$GenotypePC189:y$SexM:y$FoodQ                NA       NA
## y$PopulationFFD:y$GenotypePC200:y$SexM:y$FoodQ               NA       NA
## y$PopulationPC:y$GenotypePC200:y$SexM:y$FoodQ                NA       NA
## y$PopulationFFD:y$GenotypePC85:y$SexM:y$FoodQ                NA       NA
## y$PopulationPC:y$GenotypePC85:y$SexM:y$FoodQ                 NA       NA
## y$PopulationFFD:y$GenotypeFFD14:y$TempLow:y$FoodQ        -2.747  0.00604 **
## y$PopulationPC:y$GenotypeFFD14:y$TempLow:y$FoodQ             NA       NA
## y$PopulationFFD:y$GenotypeFFD16:y$TempLow:y$FoodQ        -0.629  0.52923
## y$PopulationPC:y$GenotypeFFD16:y$TempLow:y$FoodQ             NA       NA
## y$PopulationFFD:y$GenotypeFFD19:y$TempLow:y$FoodQ        -0.433  0.66529
## y$PopulationPC:y$GenotypeFFD19:y$TempLow:y$FoodQ             NA       NA
## y$PopulationFFD:y$GenotypeFFD2:y$TempLow:y$FoodQ         -5.376 8.10e-08 ***
## y$PopulationPC:y$GenotypeFFD2:y$TempLow:y$FoodQ              NA       NA
## y$PopulationFFD:y$GenotypeFFD22:y$TempLow:y$FoodQ        -3.027  0.00249 **
## y$PopulationPC:y$GenotypeFFD22:y$TempLow:y$FoodQ             NA       NA
## y$PopulationFFD:y$GenotypeFFD23:y$TempLow:y$FoodQ         1.541  0.12345
## y$PopulationPC:y$GenotypeFFD23:y$TempLow:y$FoodQ             NA       NA
## y$PopulationFFD:y$GenotypeFFD29:y$TempLow:y$FoodQ         2.584  0.00981 **
## y$PopulationPC:y$GenotypeFFD29:y$TempLow:y$FoodQ             NA       NA
## y$PopulationFFD:y$GenotypeFFD4:y$TempLow:y$FoodQ             NA       NA
## y$PopulationPC:y$GenotypeFFD4:y$TempLow:y$FoodQ              NA       NA
## y$PopulationFFD:y$GenotypeFFD6:y$TempLow:y$FoodQ          1.071  0.28439
## y$PopulationPC:y$GenotypeFFD6:y$TempLow:y$FoodQ              NA       NA
```

```
## y$PopulationFFD:y$GenotypePC113:y$TempLow:y$FoodQ            NA        NA
## y$PopulationPC:y$GenotypePC113:y$TempLow:y$FoodQ             NA        NA
## y$PopulationFFD:y$GenotypePC114:y$TempLow:y$FoodQ            NA        NA
## y$PopulationPC:y$GenotypePC114:y$TempLow:y$FoodQ         -0.111   0.91131
## y$PopulationFFD:y$GenotypePC12:y$TempLow:y$FoodQ             NA        NA
## y$PopulationPC:y$GenotypePC12:y$TempLow:y$FoodQ           0.931   0.35217
## y$PopulationFFD:y$GenotypePC136:y$TempLow:y$FoodQ            NA        NA
## y$PopulationPC:y$GenotypePC136:y$TempLow:y$FoodQ         -0.111   0.91195
## y$PopulationFFD:y$GenotypePC141:y$TempLow:y$FoodQ            NA        NA
## y$PopulationPC:y$GenotypePC141:y$TempLow:y$FoodQ         -0.081   0.93530
## y$PopulationFFD:y$GenotypePC155:y$TempLow:y$FoodQ            NA        NA
## y$PopulationPC:y$GenotypePC155:y$TempLow:y$FoodQ          2.237   0.02533 *
## y$PopulationFFD:y$GenotypePC167:y$TempLow:y$FoodQ            NA        NA
## y$PopulationPC:y$GenotypePC167:y$TempLow:y$FoodQ          1.238   0.21572
## y$PopulationFFD:y$GenotypePC189:y$TempLow:y$FoodQ            NA        NA
## y$PopulationPC:y$GenotypePC189:y$TempLow:y$FoodQ          0.648   0.51721
## y$PopulationFFD:y$GenotypePC200:y$TempLow:y$FoodQ            NA        NA
## y$PopulationPC:y$GenotypePC200:y$TempLow:y$FoodQ             NA        NA
## y$PopulationFFD:y$GenotypePC85:y$TempLow:y$FoodQ             NA        NA
## y$PopulationPC:y$GenotypePC85:y$TempLow:y$FoodQ              NA        NA
## y$PopulationPC:y$SexM:y$TempLow:y$FoodQ                  -0.211   0.83325
## y$PopulationFFD:y$GenotypeFFD14:y$SexM:y$TempLow:y$FoodQ     NA        NA
## y$PopulationPC:y$GenotypeFFD14:y$SexM:y$TempLow:y$FoodQ      NA        NA
## y$PopulationFFD:y$GenotypeFFD16:y$SexM:y$TempLow:y$FoodQ  0.857   0.39135
## y$PopulationPC:y$GenotypeFFD16:y$SexM:y$TempLow:y$FoodQ      NA        NA
## y$PopulationFFD:y$GenotypeFFD19:y$SexM:y$TempLow:y$FoodQ     NA        NA
## y$PopulationPC:y$GenotypeFFD19:y$SexM:y$TempLow:y$FoodQ      NA        NA
## y$PopulationFFD:y$GenotypeFFD2:y$SexM:y$TempLow:y$FoodQ      NA        NA
## y$PopulationPC:y$GenotypeFFD2:y$SexM:y$TempLow:y$FoodQ       NA        NA
## y$PopulationFFD:y$GenotypeFFD22:y$SexM:y$TempLow:y$FoodQ -0.920   0.35762
## y$PopulationPC:y$GenotypeFFD22:y$SexM:y$TempLow:y$FoodQ      NA        NA
## y$PopulationFFD:y$GenotypeFFD23:y$SexM:y$TempLow:y$FoodQ     NA        NA
## y$PopulationPC:y$GenotypeFFD23:y$SexM:y$TempLow:y$FoodQ      NA        NA
## y$PopulationFFD:y$GenotypeFFD29:y$SexM:y$TempLow:y$FoodQ     NA        NA
## y$PopulationPC:y$GenotypeFFD29:y$SexM:y$TempLow:y$FoodQ      NA        NA
## y$PopulationFFD:y$GenotypeFFD4:y$SexM:y$TempLow:y$FoodQ      NA        NA
## y$PopulationPC:y$GenotypeFFD4:y$SexM:y$TempLow:y$FoodQ       NA        NA
## y$PopulationFFD:y$GenotypeFFD6:y$SexM:y$TempLow:y$FoodQ   0.346   0.72921
## y$PopulationPC:y$GenotypeFFD6:y$SexM:y$TempLow:y$FoodQ       NA        NA
## y$PopulationFFD:y$GenotypePC113:y$SexM:y$TempLow:y$FoodQ     NA        NA
## y$PopulationPC:y$GenotypePC113:y$SexM:y$TempLow:y$FoodQ      NA        NA
## y$PopulationFFD:y$GenotypePC114:y$SexM:y$TempLow:y$FoodQ     NA        NA
## y$PopulationPC:y$GenotypePC114:y$SexM:y$TempLow:y$FoodQ  -1.173   0.24088
## y$PopulationFFD:y$GenotypePC12:y$SexM:y$TempLow:y$FoodQ      NA        NA
## y$PopulationPC:y$GenotypePC12:y$SexM:y$TempLow:y$FoodQ   -0.946   0.34422
## y$PopulationFFD:y$GenotypePC136:y$SexM:y$TempLow:y$FoodQ     NA        NA
## y$PopulationPC:y$GenotypePC136:y$SexM:y$TempLow:y$FoodQ      NA        NA
## y$PopulationFFD:y$GenotypePC141:y$SexM:y$TempLow:y$FoodQ     NA        NA
## y$PopulationPC:y$GenotypePC141:y$SexM:y$TempLow:y$FoodQ  -0.833   0.40463
## y$PopulationFFD:y$GenotypePC155:y$SexM:y$TempLow:y$FoodQ     NA        NA
## y$PopulationPC:y$GenotypePC155:y$SexM:y$TempLow:y$FoodQ   0.005   0.99620
## y$PopulationFFD:y$GenotypePC167:y$SexM:y$TempLow:y$FoodQ     NA        NA
## y$PopulationPC:y$GenotypePC167:y$SexM:y$TempLow:y$FoodQ      NA        NA
## y$PopulationFFD:y$GenotypePC189:y$SexM:y$TempLow:y$FoodQ     NA        NA
```

```
## y$PopulationPC:y$GenotypePC189:y$SexM:y$TempLow:y$FoodQ        NA        NA
## y$PopulationFFD:y$GenotypePC200:y$SexM:y$TempLow:y$FoodQ       NA        NA
## y$PopulationPC:y$GenotypePC200:y$SexM:y$TempLow:y$FoodQ        NA        NA
## y$PopulationFFD:y$GenotypePC85:y$SexM:y$TempLow:y$FoodQ        NA        NA
## y$PopulationPC:y$GenotypePC85:y$SexM:y$TempLow:y$FoodQ         NA        NA
## ---
## Signif. codes:  0 '***' 0.001 '**' 0.01 '*' 0.05 '.' 0.1 ' ' 1
##
## Residual standard error: 0.05203 on 3464 degrees of freedom
## Multiple R-squared:  0.5923, Adjusted R-squared:  0.576
## F-statistic: 36.46 on 138 and 3464 DF,  p-value: < 2.2e-16
```
